# Supplementary material for: Tandem ketone reduction in pepstatin biosynthesis reveals an F420H2–dependent statine pathway
Source: Nat Commun. 2025 May 15;16:4531. doi: 10.1038/s41467-025-59785-0 (PMC12081711; doi:10.1038/s41467-025-59785-0)
Supplement: Supplementary file 1 — Supplementary Information [file 41467_2025_59785_MOESM1_ESM.pdf]

Supplementary Information

Tandem ketone reduction in pepstatin biosynthesis reveals an $F_{420}H_2$–dependent statine pathway

Jingjun Mo[1,2]†, Asfandyar Sikandar[1]†, Haowen Zhao[1,2]†, Ghader Bashiri[3], Liujie Huo[4], Martin Empting[1,5,6], Rolf Müller[1,2,5,6], Chengzhang Fu[1,2]*

[1]Helmholtz Institute for Pharmaceutical Research Saarland (HIPS), Helmholtz Centre for Infection Research (HZI), 66123 Saarbrücken, Germany

[2]Helmholtz International Lab for Anti-Infectives, Helmholtz Center for Infection Research, 38124 Braunschweig, Germany

[3]Laboratory of Microbial Biochemistry and Biotechnology, School of Biological Sciences, University of Auckland, Private Bag 92019, Auckland, New Zealand

[4]State Key Laboratory of Microbial Technology, Helmholtz International Lab for Anti-Infectives, Shandong University, Qingdao 266237, China

[5]German Centre for Infection Research (DZIF), 38124 Braunschweig, Germany

[6]Department of Pharmacy, Saarland University, 66123 Saarbrücken, Germany

†These authors contributed equally to this work.

*Corresponding author. Email: chengzhang.fu@helmholtz-hips.de

Contents

Supplementary Figures **4**

Supplementary Fig. 1. HPLC-MS/MS fragmentation analysis of compounds **1-4**. 4

Supplementary Fig. 2. Theoretical NRPS assembly line for pepstatin based on colinearity principles. 5

Supplementary Fig. 3. Plasmid pQS9-△*pepD* and mutant *S. catenulae* -△*pepD* verification. 5

Supplementary Fig. 4. Plasmid p15A-int-*pep* and mutant *S. albus* Del14-*pep* verification. 6

Supplementary Fig. 5. PCR verification of *pepJ* gene deletion mutant. 6

Supplementary Fig. 6. PCR verification of *pepJ* gene activation mutant. 7

Supplementary Fig. 7. Pepstatin yield in different producers. 7

Supplementary Fig. 8. PCR verification of *S. albus* Del14-*pep*-△*pepI*. 8

Supplementary Fig. 9. HPLC-MS/MS fragmentation analysis of compounds **5-8**. 8

Supplementary Fig. 10. HPLC-MS/MS fragmentation analysis of compounds **9-12**. 9

Supplementary Fig. 11. PepI and PepI homologues. 9

Supplementary Fig. 12. HPLC-MS/MS fragmentation analysis of compounds **17-20**. 10

Supplementary Fig. 13. HPLC-MS analysis of PepI *in vitro* reactions. 10

Supplementary Fig. 14. HPLC-MS analysis of kvPepI *in vitro* reactions. 11

Supplementary Fig. 15. HPLC–MS analysis of PepI reaction using Del14-Δ*pepI* fermentation broth crude extracts as substrates. 12

Supplementary Fig. 16. HPLC-MS/MS fragmentation analysis of compounds **13-16**. 12

Supplementary Fig. 17. SDS-PAGE analysis of PepI, kvPepI and kvPepI mutants. 13

Supplementary Fig. 18. HPLC-MS analysis of statine using the advanced Marfey's method[4] 14

Supplementary Fig. 19. Structural analysis of kvPepI crystal structure.. 15

Supplementary Fig. 20. Structural analysis of kvPepI – F_{420} co-crystal structure.. 16

Supplementary Fig. 21. Structural analysis of kvPepI – F_{420} cocrystal structure and kvPepI^{H62A}. 17

Supplementary Fig. 22. Binding poses of compound **9** in kvPepI. 18

Supplementary Fig. 23. Structural analysis of kvPepI – F_{420} cocrystal structure with **9** and kvPepI^{Y122A}–F_{420} complex. 19

Supplementary Fig. 24. Evaluation of the activity of kvPepI and its mutants on two reduction steps was conducted using HPLC-MS analysis to determine the compound ratio between substrate **9** and products **13** and **17**. 21

Supplementary Fig. 25. Initial orientation of β-keto amide motif of PreSta3 (green carbons) of compound **9** (white carbons) in the active site in the modelling process. 22

NMR spectra **23**

Supplementary Fig. 26. ^{1}H-NMR spectrum of compound **1** 23

Supplementary Fig. 27. ^{13}C -NMR spectrum of compound **1** 23

Supplementary Fig. 28. ^{1}H,^{13}C-HSQC spectrum of compound **1** 24

Supplementary Fig. 29. ^{1}H,^{13}C-HMBC spectrum of compound **1** 24

Supplementary Fig. 30. ^{1}H-NMR spectrum of compound **2** 25

Supplementary Fig. 31. ^{13}C-NMR spectrum of compound **2** 25
Supplementary Fig. 32. ^{1}H,^{13}C-HSQC spectrum of compound **2** 26
Supplementary Fig. 33. ^{1}H,^{1}H-TCOSY spectrum of compound **2** 26
Supplementary Fig. 34. ^{1}H-NMR spectrum of compound **3** 27
Supplementary Fig. 35. ^{13}C-NMR spectrum of compound **3** 27
Supplementary Fig. 36. ^{1}H,^{13}C-HSQC spectrum of compound **3** 28
Supplementary Fig. 37. ^{1}H,^{1}H-HMBC spectrum of compound **3** 28
Supplementary Fig. 38. ^{1}H,^{1}H-TCOSY spectrum of compound **3** 29
Supplementary Fig. 39. ^{1}H-NMR spectrum of compound **4** 29
Supplementary Fig. 40. ^{13}C-NMR spectrum of compound **4** 30
Supplementary Fig. 41. ^{1}H,^{13}C-HSQC spectrum of compound **4** 30
Supplementary Fig. 42. ^{1}H,^{1}H-TCOSY spectrum of compound **4** 31
Supplementary Fig. 43. ^{1}H-NMR spectrum of compound **9** 32
Supplementary Fig. 44. ^{13}C-NMR spectrum of compound **9** 32
Supplementary Fig. 45. ^{1}H,^{13}C-HSQC spectrum of compound **9** 33
Supplementary Fig. 46. ^{1}H,^{13}C-HMBC spectrum of compound **9** 33
Supplementary Fig. 47. ^{1}H,^{1}H-COSY spectrum of compound **9** 34
Supplementary Fig. 48. ^{1}H-NMR spectrum of compound **10** 35
Supplementary Fig. 49. ^{13}C-NMR spectrum of compound **10** 35
Supplementary Fig. 50. ^{1}H,^{13}C-HSQC spectrum of compound **10** 36
Supplementary Fig. 51. ^{1}H,^{13}C-HMBC spectrum of compound **10** 36
Supplementary Fig. 52. COSY spectrum of compound **10** 37
Supplementary Fig. 53. ^{1}H-NMR spectrum of compound **11** 38
Supplementary Fig. 54. ^{13}C-NMR spectrum of compound **11** 38
Supplementary Fig. 55. ^{1}H,^{13}C-HSQC spectrum of compound **11** 39
Supplementary Fig. 56. ^{1}H,^{13}C-HMBC spectrum of compound **11** 39
Supplementary Fig. 57. ^{1}H,^{1}H-COSY spectrum of compound **11** 40
Supplementary Fig. 58. ^{1}H-NMR spectrum of compound **12** 41
Supplementary Fig. 59. ^{13}C-NMR spectrum of compound **12** 41
Supplementary Fig. 60. ^{1}H,^{13}C-HSQC spectrum of compound **12** 42
Supplementary Fig. 61. ^{1}H,^{13}C-HMBC spectrum of compound **12** 42
Supplementary Fig. 62. ^{1}H,^{1}H-COSY spectrum of compound **12** 43
Supplementary Fig. 63. ^{1}H-NMR spectrum of compound **13** 44
Supplementary Fig. 64. ^{1}H,^{13}C-HSQC spectrum of compound **13** 44
Supplementary Fig. 65. ^{1}H,^{13}C-HMBC spectrum of compound **13** 45
Supplementary Fig. 66. ^{1}H,^{1}H-COSY spectrum of compound **13** 45
Supplementary Fig. 67. ^{1}H,^{1}H-TOCSY spectrum of compound **13** 46
Supplementary Fig. 68. ^{1}H NMR spectrum of compound **17** 47

Supplementary Fig. 69. ^{1}H,^{13}C-HSQC spectrum of compound **17** 47
Supplementary Fig. 70. ^{1}H,^{13}C-HMBC spectrum of compound **17** 48
Supplementary Fig. 71. ^{1}H,^{1}H-COSY spectrum of compound **17** 48
Supplementary Fig. 72. ^{1}H,^{1}H-TOCSY spectrum of compound **17** 49
Supplementary Fig. 73. ^{1}H NMR spectrum of compound **13**-d_1 50
Supplementary Fig. 74. ^{1}H NMR spectrum of compound **17**-d_2 50
Supplementary Tables 51
Supplementary Table 1. Bacterial strains used in this study. 51
Supplementary Table 2. Plasmids used in this study 52
Supplementary Table 3. Primers used in this study. Restriction sites are underlined. 53
Supplementary Table 4. Gene annotation of *pep* BGC 55
Supplementary Table 5. Substrate specificity analysis of the three A domains from *pep* NRPSs. 56
Supplementary Table 6. Classification of *pep*-like BGCs. 57
Supplementary Table 7. X-ray data collection and refinement statistics 58
NMR Tables 60
Supplementary Table 8. NMR spectroscopic data of compounds **1-4** (methanol-d_4) 60
Supplementary Table 9. NMR spectroscopic data of compounds **9-12** 62
Supplementary Table 10. NMR spectroscopic data of compounds **13**, **13**-d_1, **17** and **17**-d_2 64
Supplementary References 66

Supplementary Figures

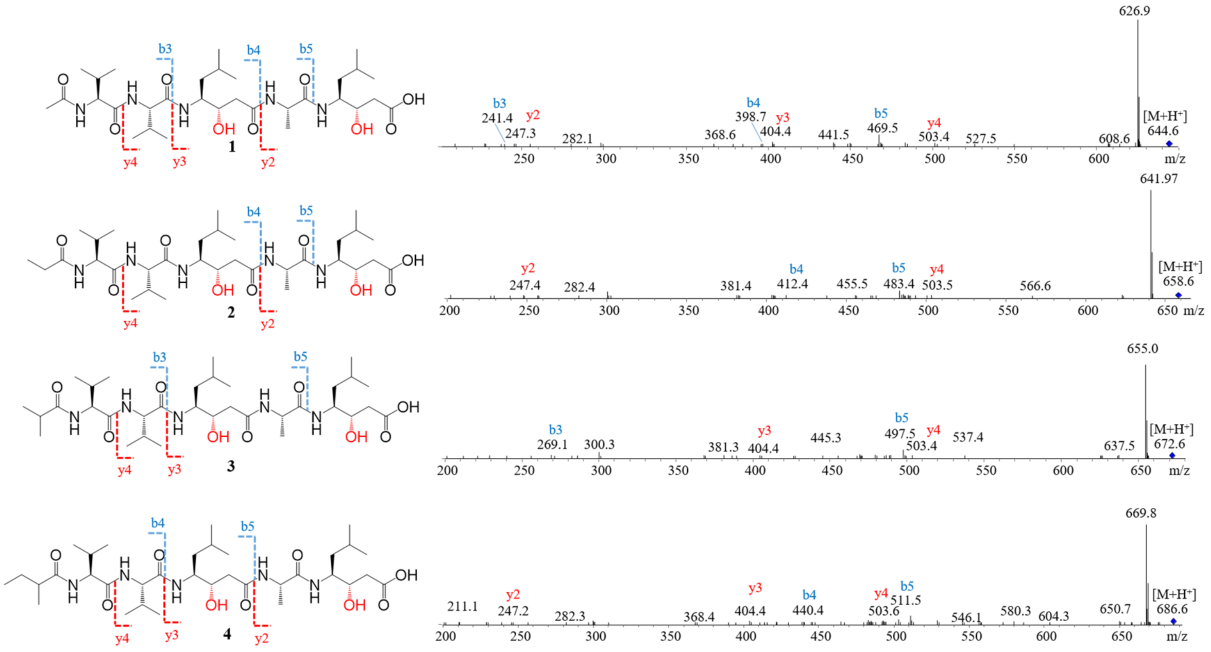

Supplementary Fig. 1. HPLC-MS/MS fragmentation analysis of compounds **1-4**.

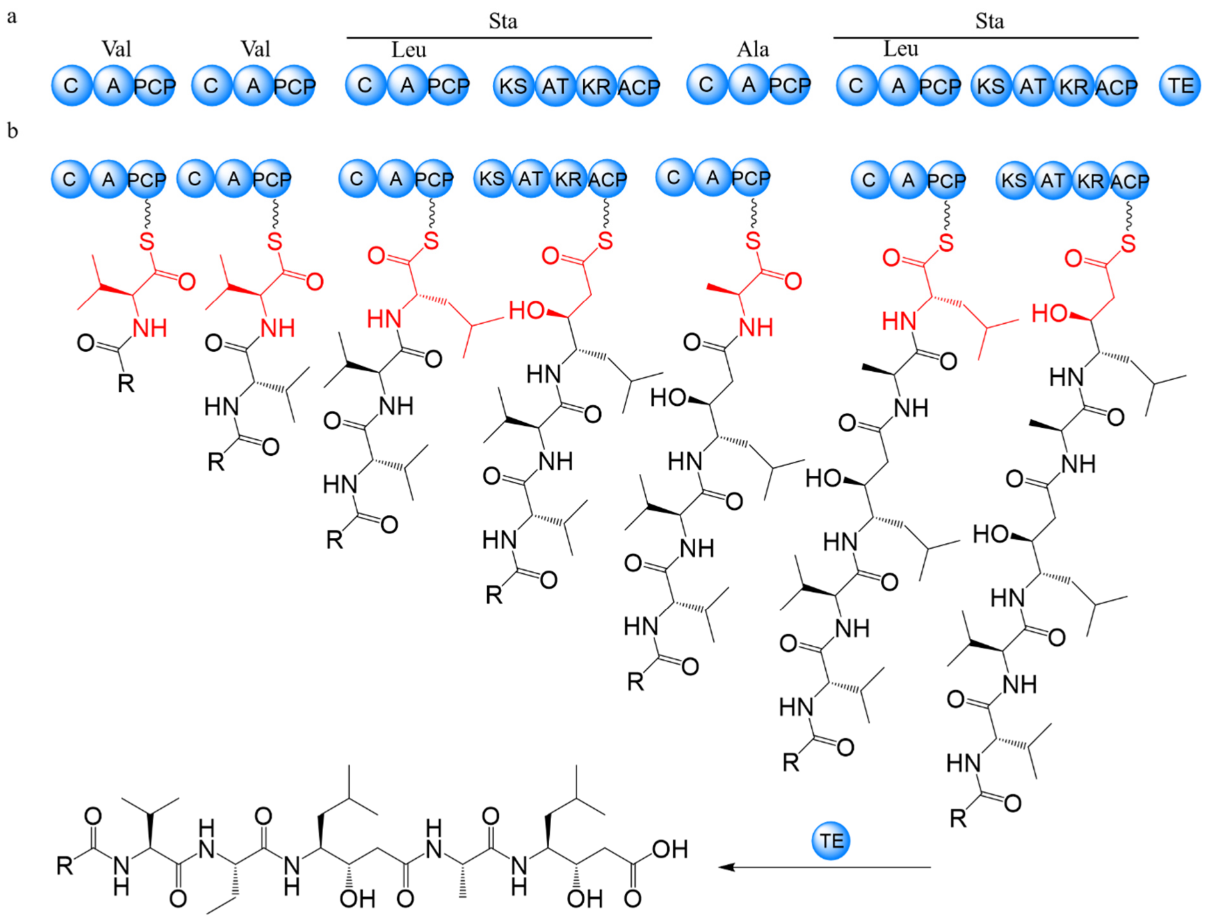

Supplementary Fig. 2. Theoretical NRPS assembly line for pepstatin based on colinearity principles. **a.** Theoretical pepstatin biosynthesis NRPS BGC schematic diagram; **b.** Theoretical pepstatin biosynthesis pathway. C: condensation domain; A: adenylation domain; PCP: peptidyl carrier protein; KS: ketosynthase; AT: acyltransferase; KR: ketoreductase; ACP: acyl carrier protein; TE: thioesterase.

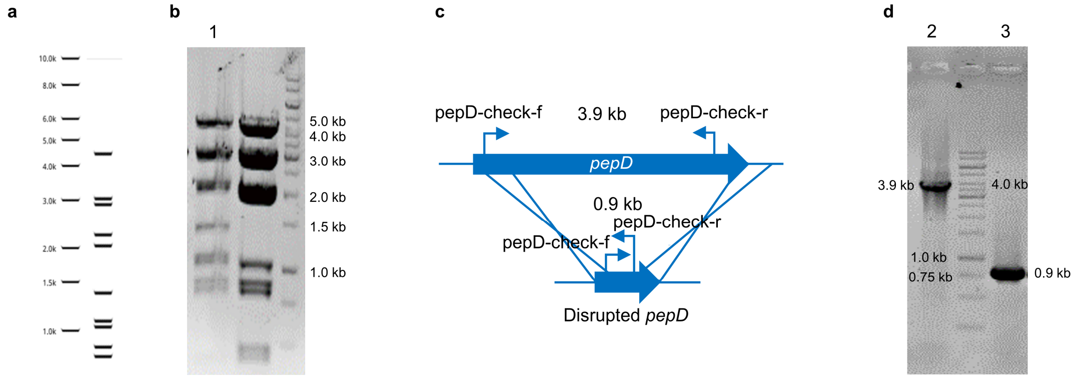

Supplementary Fig. 3. Plasmid pQS9-△*pepD* and mutant *S. catenulae* -△*pepD* verification. **a.** *In silico* digestion of pQS9-△*pepD* with *Sal*I; **b.** 1. pQS9-△*pepD* digested with *Sal*I; **c.** *pepD* gene disruption schematic diagram; **d.** 2-3. PCR verification with primers pepD-check-f/ pepD-check-r 2. *S. catenulae*; 3. *S. catenulae*-△*pepD*.

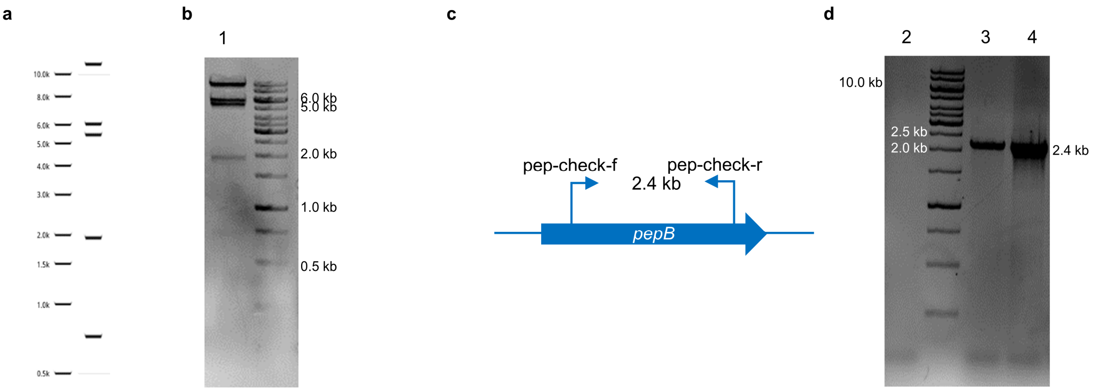

Supplementary Fig. 4. Plasmid p15A-int-*pep* and mutant *S. albus* Del14-*pep* verification. **a.** *In silico* digestion of p15A-int-*pep* with *Nco*I; **b.** 1. p15A-int-*pep* digested with *Nco*I; **c.** BGC *pep* PCR verification schematic diagram; **d.** 2-4. PCR verification with primers pep-check-f/ pep-check-r 2. *S. albus* Del14; 3. *S. catenulae*; 4. *S. albus* Del14-*pep*.

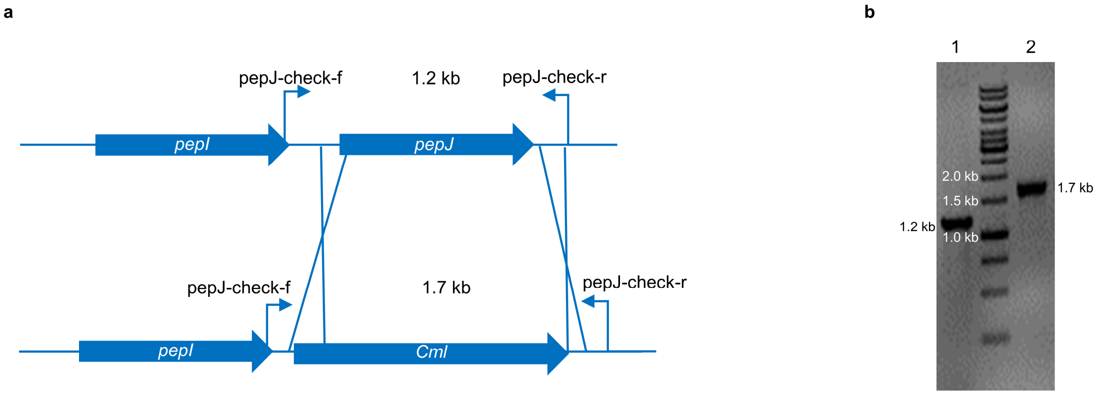

Supplementary Fig. 5. PCR verification of *pepJ* gene deletion mutant. **a.** *pepJ* gene deletion schematic diagram; **b.** *pepJ* gene deletion mutant PCR verification 1. *S. albus* Del14-*pep*; 2. *S. albus* Del14-*pep*-△*pepJ*.

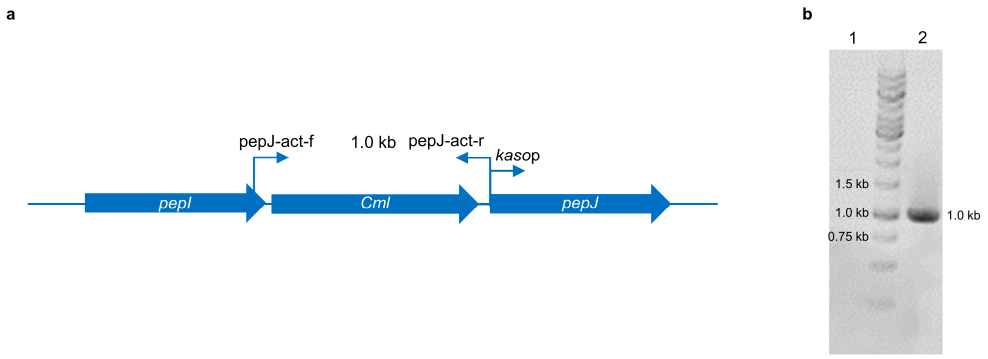

Supplementary Fig. 6. PCR verification of *pepJ* gene activation mutant. **a.** *pepJ* gene activation schematic diagram; **b.** *pepJ* gene activation mutant PCR verification 1. *S. albus* Del14-*pep*; 2. *S. albus* Del14-*pep*-*pepJ*-act.

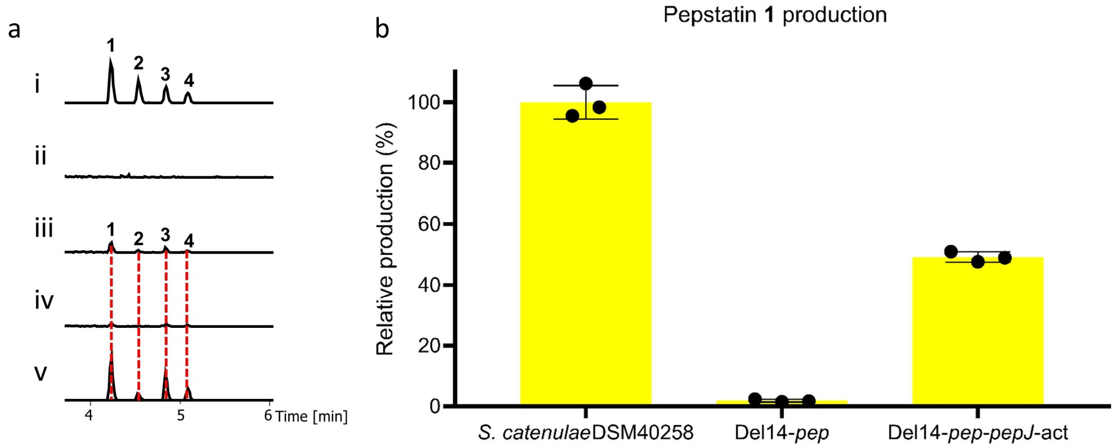

Supplementary Fig. 7. Pepstatin yield in different producers. **a.** UPLC-HRMS analysis (extracted ion chromatogram (EIC)) of Pepstatin congeners (**1-4**: **1**, $[M + H]^+ = 644.42$; **2**, $[M + H]^+ = 658.44$; **3**, $[M + H]^+ = 672.45$ and **4**, $[M + H]^+ = 686.47$) produced by *Streptomyces catenulae* DSM40258 (i); Knockingout *pepD* abolished pepstatin **1-4** production (ii); Pepstatin congeners (**1-4**) produced by heterologous expression of BGC *pep* in Del14 (Del14-*pep*) (iii); The production of **1-4** decreased by *pepJ* deletion in Del14-*pep*-△*pepJ* (iv); The production of **1-4** increased in Del14-*pep*-*pepJ*-act by promoter exchange of *pepJ* (v). **b.**The production of pepstatin **1** in the *pepJ* activation mutants *S. albus* Del14-*pep*-*pepJ*-act (right) was enhanced by 25-fold on average compared to *S. albus* Del14-*pep* (middle) but still lower than in the WT strain *S. catenulae* DSM40258 (left). The production in each culture was determined by calculating the sum of the peak area, while the yield in the WT strain was denoted as 100±5.5%. The yield from heterologously expressed mutant *S. albus* Del14-*pep* is 2±0.5%. By placing regulator gene *pepJ* under the control of a constitutive promoter in the heterologously expressed mutant, the yield of *S. albus* Del14-*pep*-*pepJ*-act is 49±1.7%. Three biological replicates (n=3 independent experiments) were performed. All error bars represent SEM. Source data are provided as a Source Data file.

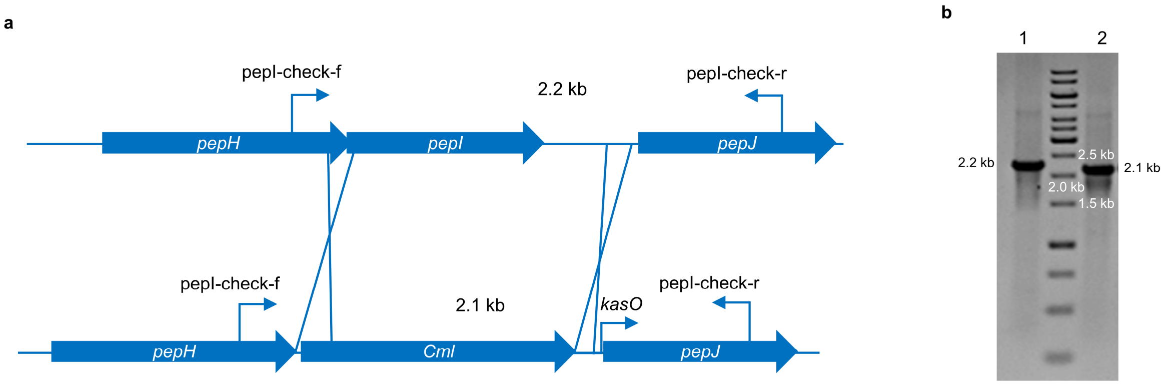

Supplementary Fig. 8. PCR verification of *S. albus* Del14-*pep*-△*pepI*. **a.** *pepI* gene deletion schematic diagram; **b.** *pepI* gene deletion mutant PCR verification 1. *S. albus* Del14-*pep*; 2. *S. albus* Del14-*pep*-△*pepI*.

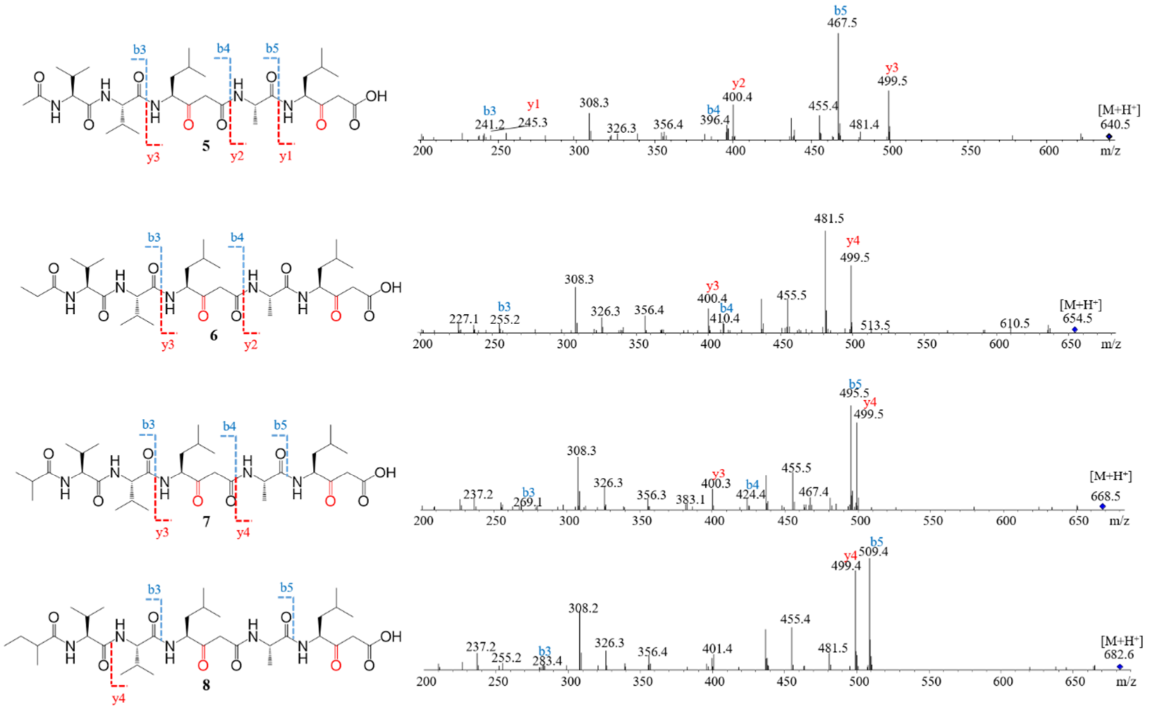

Supplementary Fig. 9. HPLC-MS/MS fragmentation analysis of compounds **5-8.**

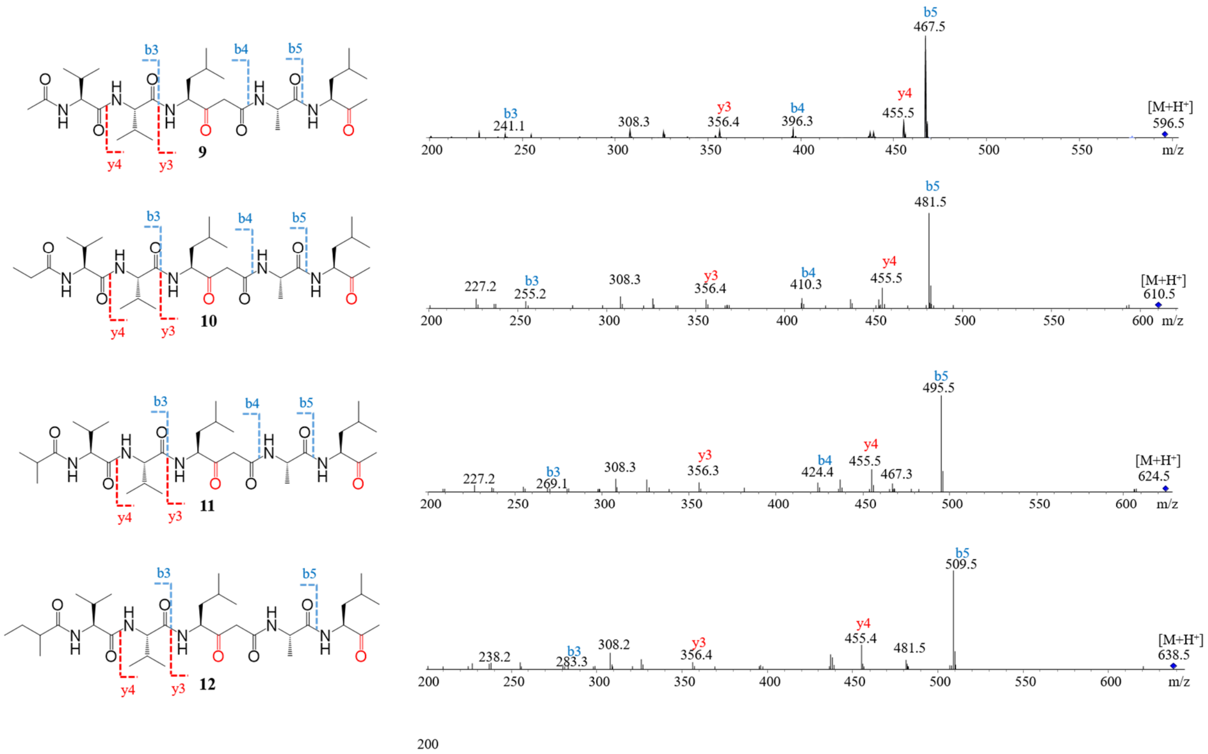

Supplementary Fig. 10. HPLC-MS/MS fragmentation analysis of compounds **9-12**.

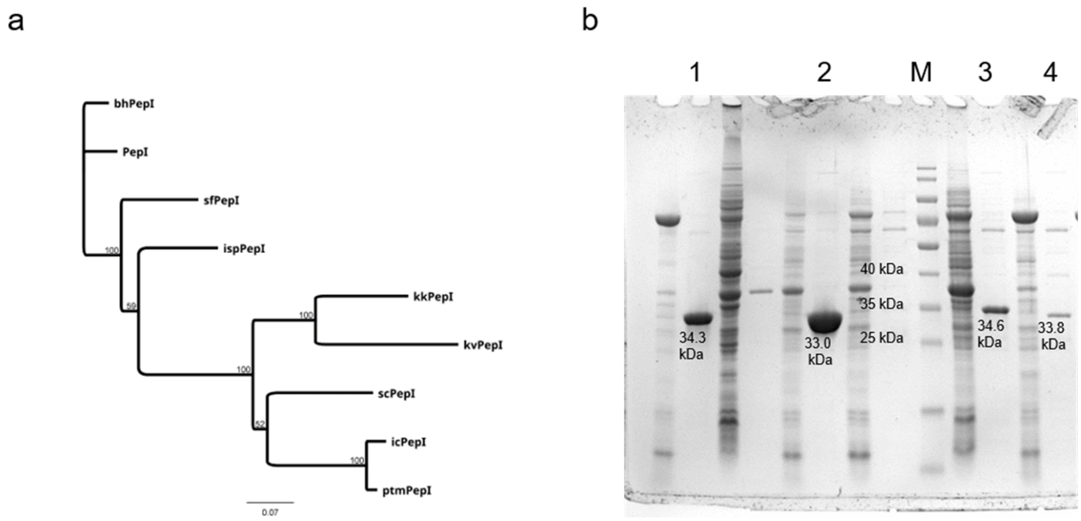

Supplementary Fig. 11. PepI and PepI homologues. a. Alignment consensus phylogenetic tree of PepI and analogs (bhPepI from *Streptomyces* sp. BHT-5-2; sfPepI from *Streptomyces* sp. SF28; ispPepI from *Streptomyces varsoviensis* NRRL ISP-5346; kkPepI from *Kitasatospora kifunensis*; kvPepI from *Kitasatospora viridis*; scPepI from *Streptomyces cattleya*; icPepI from *Streptomyces* sp. ICBB 8177; ptmPepI from *Streptomyces* sp. PTM05;) from *pep*-like BGCs. Genetic distance model: Jukes-Cantor, Tree build method: Neighbor-Joining, Resampling method: Bootstrap, Random seed: 443,376, Scale bar: evolutionary distance of amino acid residue per position in the sequence. **b.** SDS-PAGE analysis of

PepI and PepI homologues 1. sfPepI Scientific™ PageRuler™ Prestained Protein Ladder, 10 to 180 kDa. 3. svPepI 34.6 kDa (from *Streptomyces varsoviensis*[3]); 4. PepI 33.8 kDa.

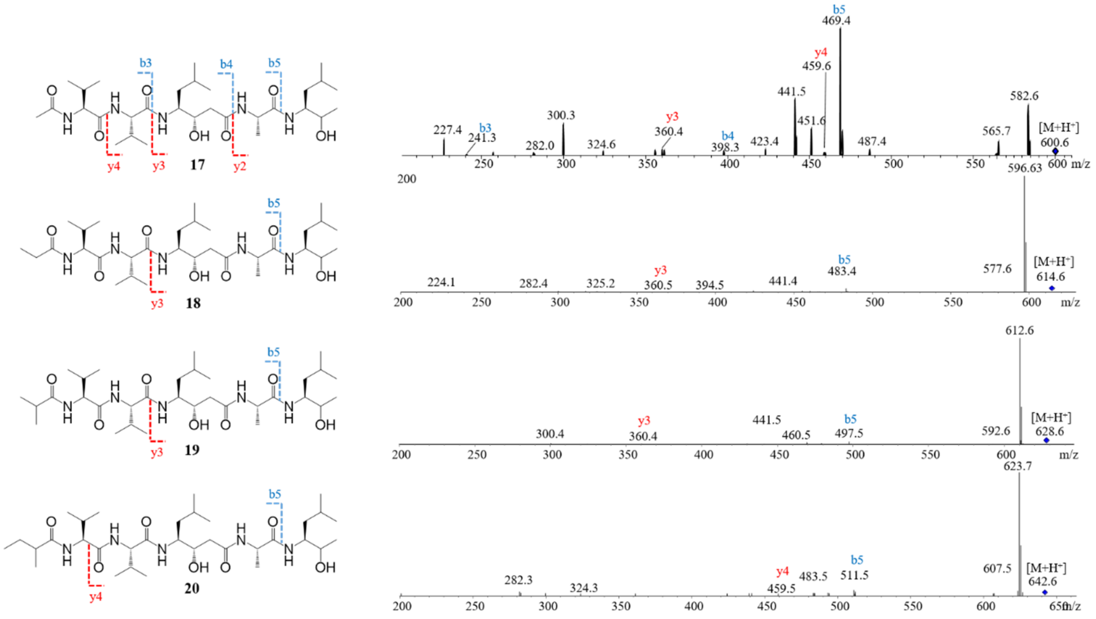

Supplementary Fig. 12. HPLC-MS/MS fragmentation analysis of compounds **17-20.**

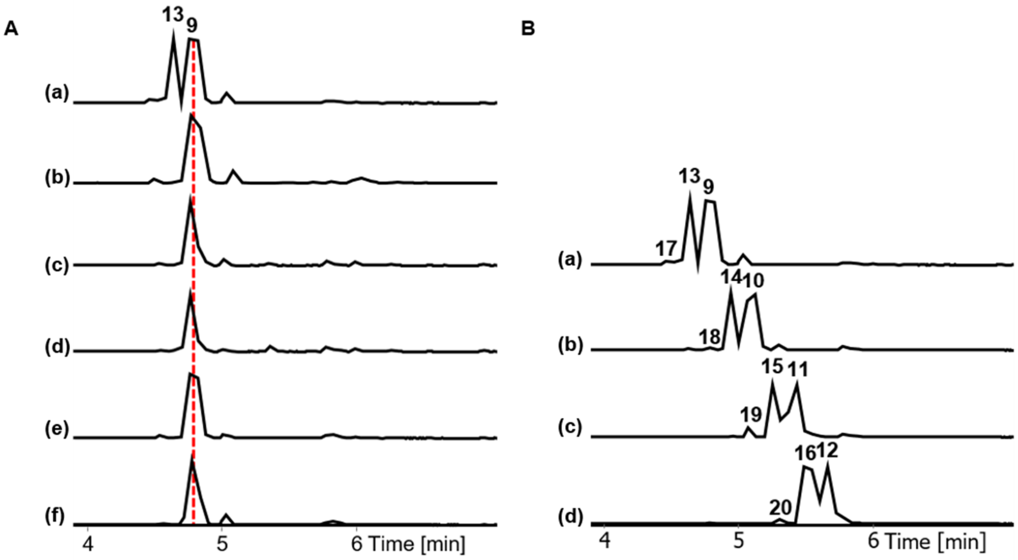

Supplementary Fig. 13. HPLC-MS analysis of PepI *in vitro* reactions. **A** Flavin cofactor preference and F_{420} recycle. (**a**) PepI+**9**+F_{420}+FGD+G6P; (**b**) PepI+**9**+FGD+G6P; (**c**) PepI+**9**+FMN+NADH; (**d**) PepI+**9**+FAD+NADH; (**e**) PepI+**9**+F_{420}+FGD; (**f**) PepI+**9**+F_{420}+G6P. **B.** $F_{420}H_2$-dependent PepI *in vitro* ketoreduction with different substrates (**a**) **9** transformed to **13**, **17;** (**b**) **10** transformed to **14**, **18;** (**c**) **11** transformed to **15**, **19;** (**d**) **12** transformed to **16**, **20**. Base peak chromatograms (BPC) were shown. **9**, $[M + H]^+$ = 596.40; **10**, $[M + H]^+$ = 610.42; **11**, $[M + H]^+$ = 624.44; **12**, $[M + H]^+$ = 638.45; **13**, [M +

H]$^+$ = 598.42; **14**, [M + H]$^+$ = 612.44; **15**, [M + H]$^+$ = 626.45; **16**, [M + H]$^+$ = 640.47; **17**, [M + H]$^+$ = 600.43; **18**, [M + H]$^+$ = 614.45; **19**, [M + H]$^+$ = 628.46 and **20**, [M + H]$^+$ = 642.48 were analyzed.

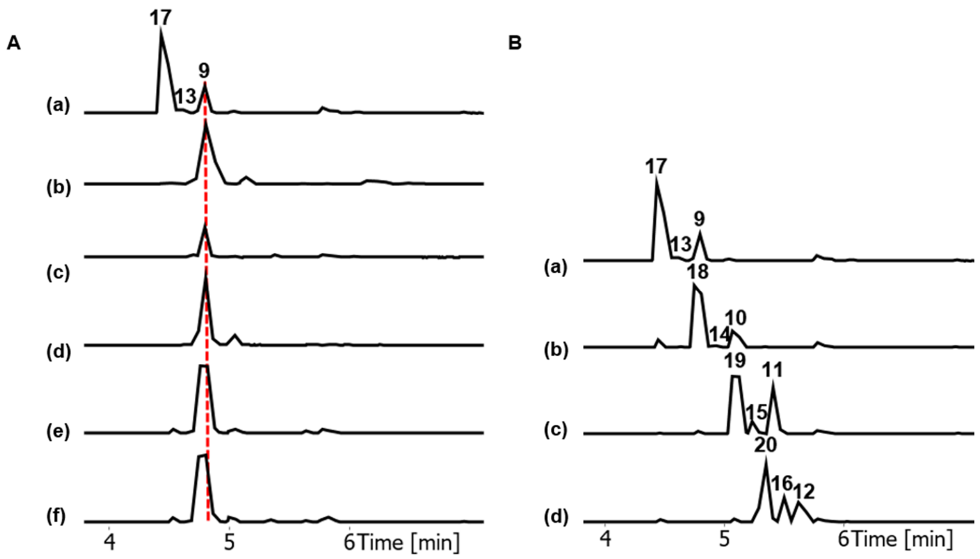

Supplementary Fig. 14. HPLC-MS analysis of kvPepI *in vitro* reactions. **A** Flavin cofactor preference and F_{420} recycle. (**a**) kvPepI+**9**+F_{420}+FGD+G6P; (**b**) kvPepI+**9**+FGD+G6P; (**c**) kvPepI+**9**+FMN+NADH; (**d**) kvPepI+**9**+FAD+NADH; (**e**) kvPepI+**9**+F_{420}+FGD; (**f**) kvPepI+**9**+F_{420}+G6P; **B** $F_{420}H_2$-dependent kvPepI *in vitro* ketoreduction with different substrates (**a**) **9** transformed to **13**, **17;** (**b**) **10** transformed to **14**, **18;** (**c**) **11** transformed to **15**, **19;** (**d**) **12** transformed to **16**, **20**. Base peak chromatograms (BPC) were shown. **9**, [M + H]$^+$ = 596.40; **10**, [M + H]$^+$ = 610.42; **11**, [M + H]$^+$ = 624.44; **12**, [M + H]$^+$ = 638.45; **13**, [M + H]$^+$ = 598.42; **14**, [M + H]$^+$ = 612.44; **15**, [M + H]$^+$ = 626.45; **16**, [M + H]$^+$ = 640.47; **17**, [M + H]$^+$ = 600.43; **18**, [M + H]$^+$ = 614.45; **19**, [M + H]$^+$ = 628.46 and **20**, [M + H]$^+$ = 642.48 were analyzed.

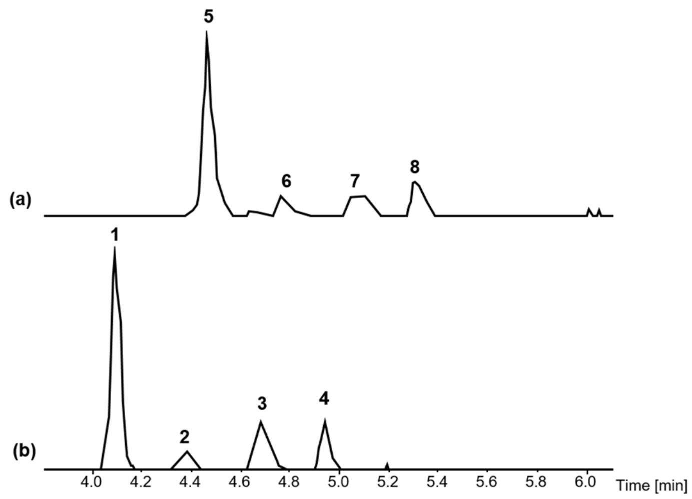

Supplementary Fig. 15. HPLC–MS analysis of PepI reaction using Del14-Δ*pepI* fermentation broth crude extracts as substrates. **(a)**. Del14-Δ*pepI* crude extract+boiled PepI and cofactor system **(b)**. Del14-Δ*pepI* crude extract+PepI and cofactor system. Extracted ion chromatograms (EIC): **5**, $[M + H]^+$ = 640.39; **6**, $[M + H]^+$ = 654.40; **7**, $[M + H]^+$ = 668.42; **8**, $[M + H]^+$ = 682.44; **1**, $[M + H]^+$ = 644.42; **2**, $[M + H]^+$ = 658.44; **3**, $[M + H]^+$ = 672.45 and **4**, $[M + H]^+$ = 686.47; were analyzed.

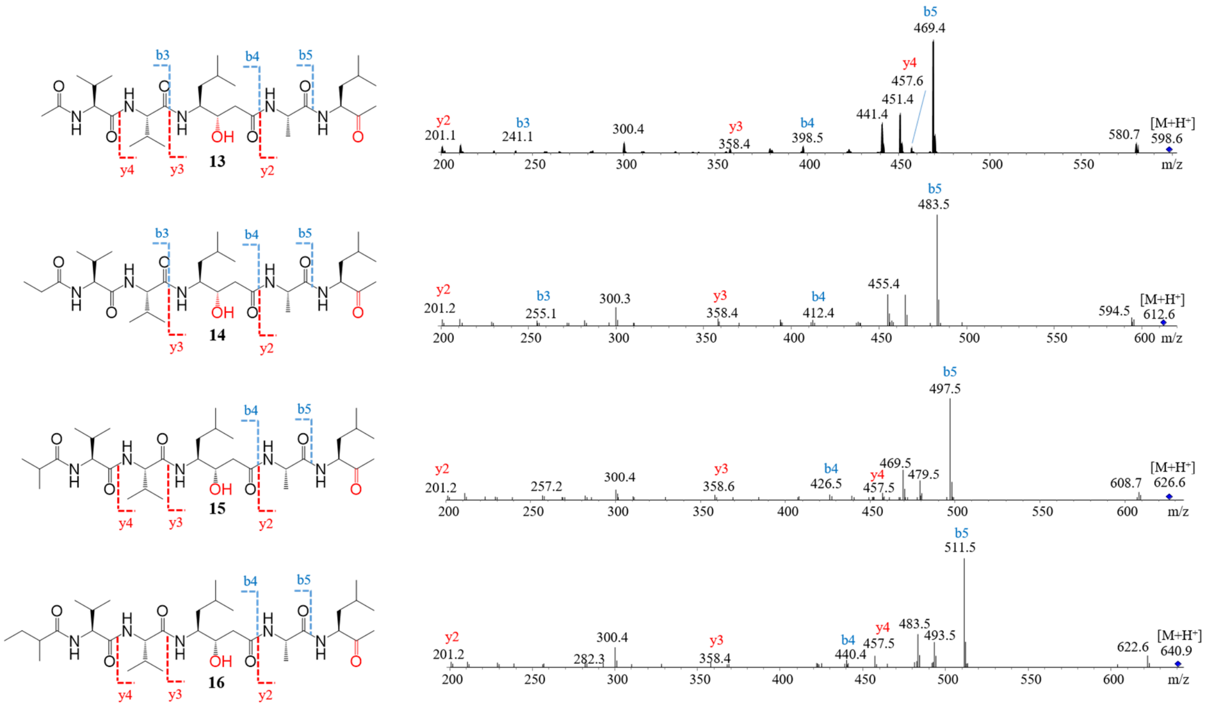

Supplementary Fig. 16. HPLC-MS/MS fragmentation analysis of compounds **13-16.**

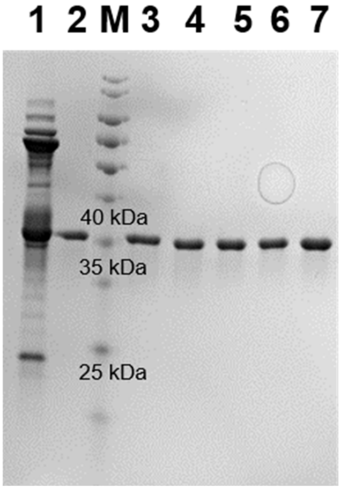

Supplementary Fig. 17. SDS-PAGE analysis of PepI, kvPepI and kvPepI mutants. 1. PepI 33.8 kDa; 2. kvPepI 33.0 kDa; 3. kvPepI^{H62A} 32.7 kDa; 4. kvPepI^{Y122A} 32.6 kDa; 5. kvPepI^{Y122F} 32.7 kDa; 6. kvPepI^{Q229A} 32.6 kDa; 7. kvPepI^{Q289A} 32.9 kDa. M. Thermo Scientific™ PageRuler™ Prestained Protein Ladder, 10 to 180 kDa).

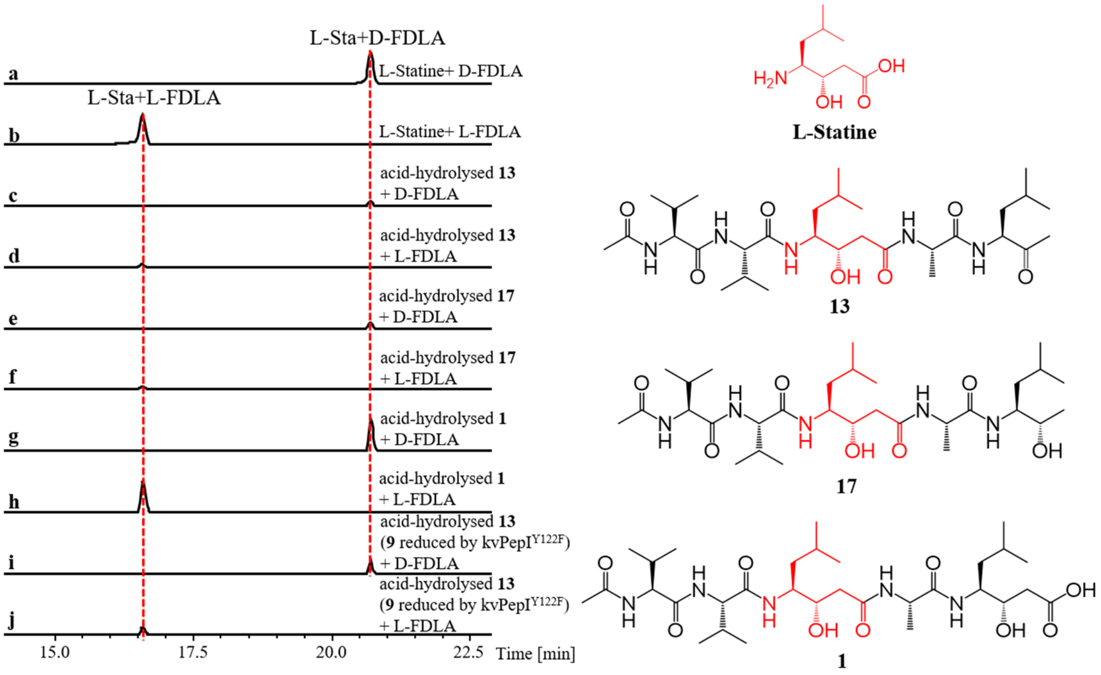

Supplementary Fig. 18. HPLC-MS analysis of statine using the advanced Marfey's method[4]. EIC $[M+H]^+ = 470.22$ **a.** L-statine+D-FDLA; **b.** L-statine+L-FDLA; **c.** acid-hydrolysed **13** (**9** reduced by kvPepI)+ D-FDLA; **d.** acid-hydrolysed **13** (**9** reduced by kvPepI)+L-FDLA; **e.** acid-hydrolysed **17**+D-FDLA; **f.** acid-hydrolysed **17**+L-FDLA; **g.** acid-hydrolysed **1**+D-FDLA; **h.** acid-hydrolysed **1**+L-FDLA; **i.** **13** (**9** reduced by kvPepI^{Y122F})+D-FDLA; **j.** **13** (**9** reduced by kvPepI^{Y122F})+L-FDLA.

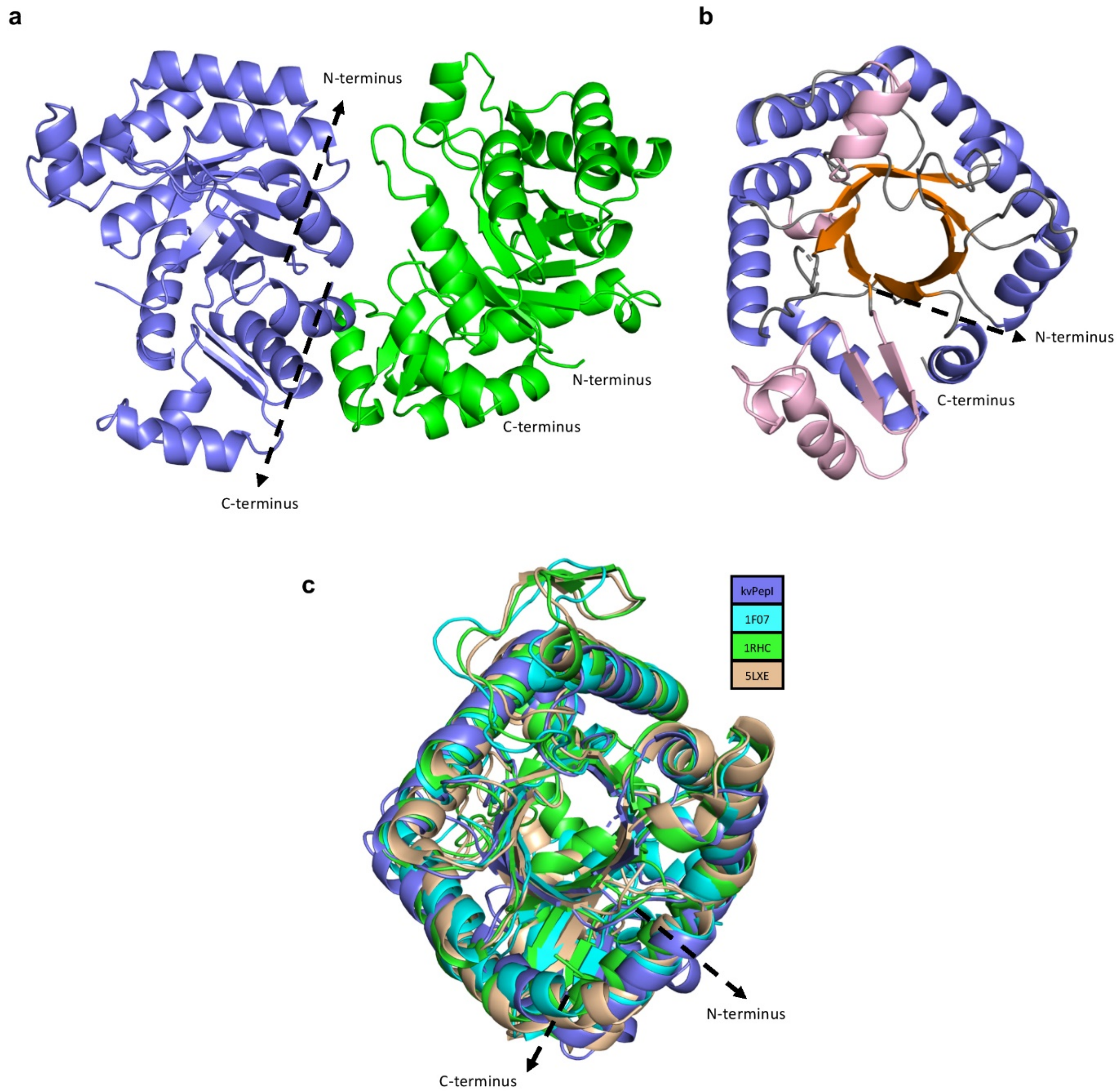

Supplementary Fig. 19. Structural analysis of kvPepI crystal structure. **a**, A dimer comprising the asymmetric unit is shown as ribbon model with the monomers coloured green and slate. **b**, Cartoon representation of kvPepI monomer showing the TIM-barrel fold (colour scheme: α-helices, orange; β-strands, slate; and loops, grey) and the additional structural inserts (pink). **c**, Superposition of kvPepI structure (cyan) with other members of Class I F_{420} – dependent enzymes (PDB IDs: 1F07 (cyan), 1RHC (green) and 5LXE (wheat)) . Cα RMSDs were approx. 2.9 Å (1F04) 2.3 Å (1RHC) and 3.2 Å (5LXE) over the entire length of the protein. The N- and C-terminus are indicated.

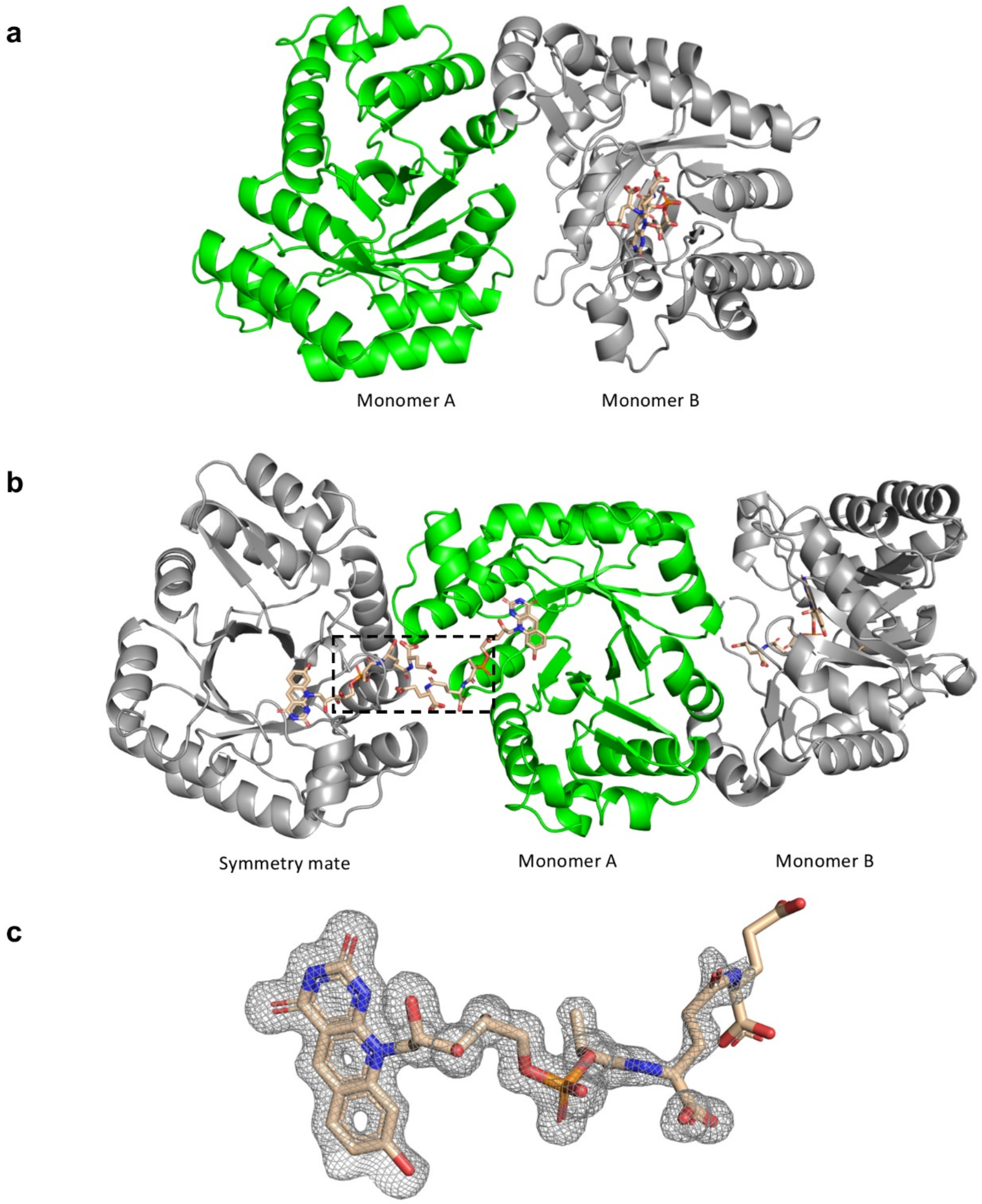

Supplementary Fig. 20. Structural analysis of kvPepI – F_{420} co-crystal structure. **a**, A dimer comprising the asymmetric unit is shown as ribbon model with the monomers (A and B) coloured green and grey. F_{420} (shown as sticks) is only observed in one of the monomers in the asymmetric unit due to crystallization artifact. **b**, Crystallization artifact preventing F_{420} binding. The bound F_{420} in the flanking symmetry related monomer likely leads to a clash with F_{420} (dotted box) modelled in monomer A by superposition of monomer B onto monomer A. **c**, Polder map (grey isomesh) of F_{420} contoured at 3.5σ.

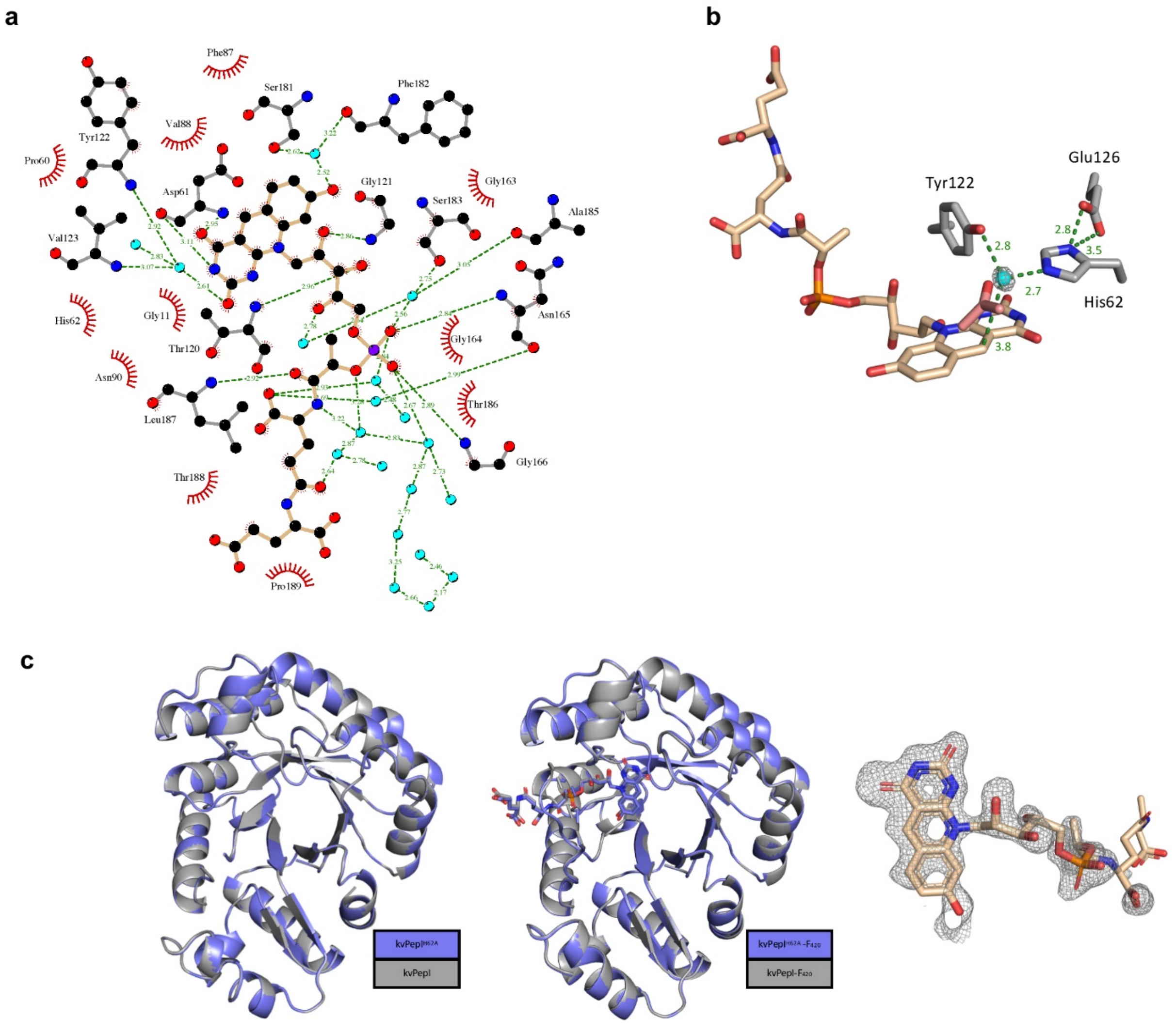

Supplementary Fig. 21. Structural analysis of kvPepI – F_{420} cocrystal structure and kvPepI^{H62A}. **a**, LigPlot diagram of the interactions between kvPepI (grey) and F_{420} (wheat). Water molecules and atoms are represented as circles (carbon black, oxygen red, nitrogen blue, and water cyan). Hydrogen bonds are shown as dashed green lines with distances given in Å. Hydrophobic interactions are depicted as red spoked arcs. **b**, The ordered water molecule (HOH570, cyan; $2F_O$-F_C electron density map shown as grey isomesh contoured at 2σ) found in close proximity to F_{420}, His62 and Tyr122. Glu126 likely help stabilise the positive charge on His62. Adf bound F_{420}-acetone adduct (PDB ID: 1RHC) was superposed to the kvPepI – F_{420} structure. The carbonyl of acetone adduct lies next to the ordered water molecule observed in kvPepI – F_{420} complex structure. For clarity only acetone is shown as stick (pink). **c**, Structural analysis of kvPepI^{H62A} and kvPepI^{H62A} – F_{420} cocrystal structure. Superposition of kvPepI and KvPepI – F_{420} with kvPepI^{H62A} and KvPepI^{H62A} – F_{420} complex structure. Cα RMSD were approx. 0.14 Å (kvPepI^{H62A} vs. kvPepI; left) and 0.1 Å (kvPepI^{H62A} – F_{420} vs. kvPepI - F_{420}; middle) over the entire length of the protein. Polder map (grey isomesh; contoured at 3σ) of F_{420} bound to kvPepI^{H62A} is shown on the right.

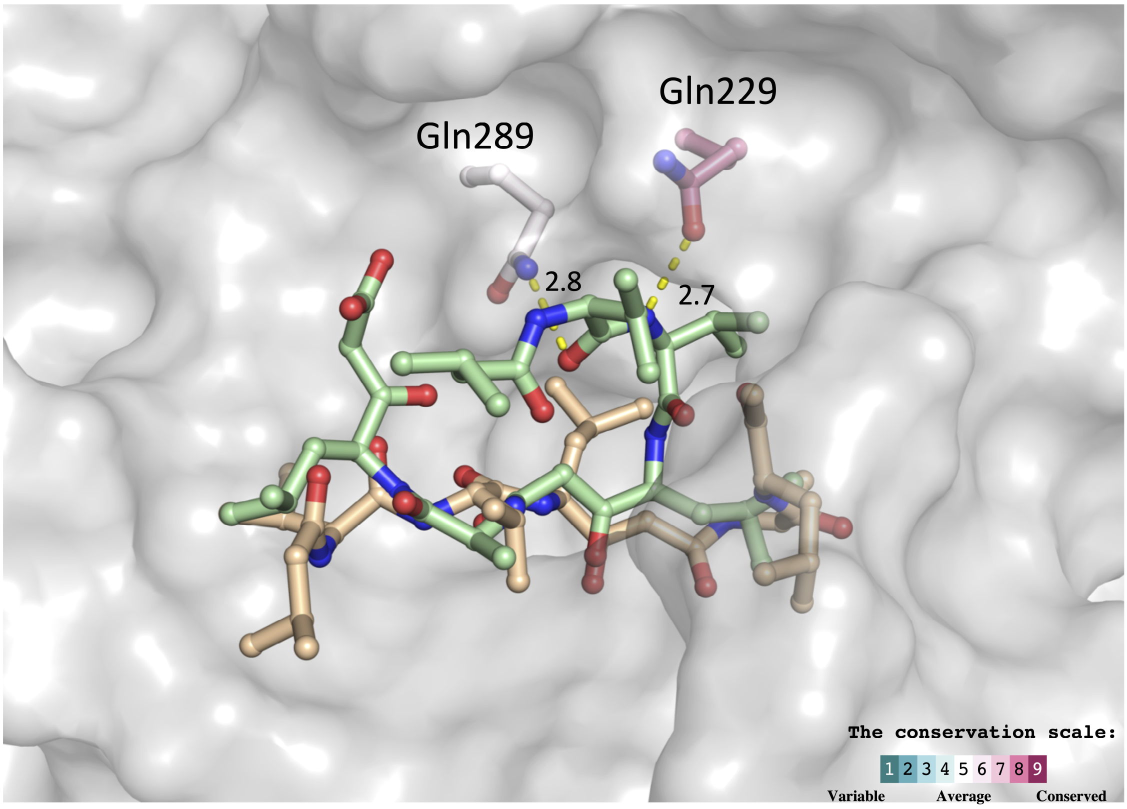

Supplementary Fig. 22. Binding poses of compound **9** in kvPepI. **9** is shown as sticks (conformation: U-shaped, pale green; linear, wheat) while kvPepI substrate binding pocket is shown as surface. The residues (Gln229 and Gln289) involved in H-bond interactions with the amide bond of Val1 of U-shaped **9** are shown as sticks. Hydrogen bond interactions are depicted with dotted yellow lines with distances given in Å. The residues are colored according to the conservation score calculated using the ConSurf server.

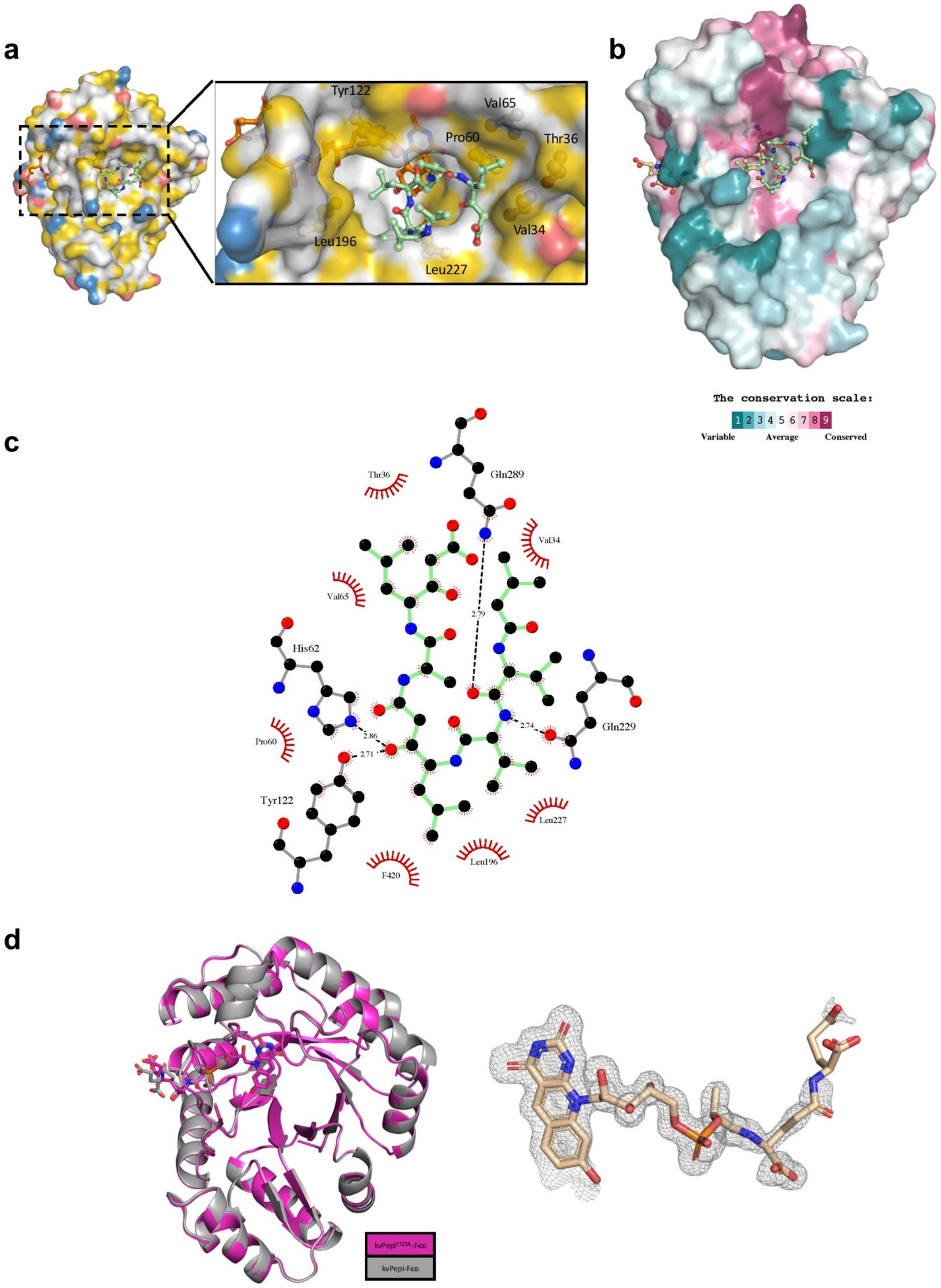

Supplementary Fig. 23. Structural analysis of kvPepI – F_{420} cocrystal structure with **9** and kvPepI^{Y122A}–F_{420} complex. **a**, Surface representation of kvPepI– F_{420} – **9** model coloured to highlight hydrophobicity and charge using the YRB scheme (hydrocarbons groups without polar substitutions, yellow; negatively charged oxygens of glutamate and aspartate, red; nitrogens of positively charged functional groups of lysine and arginine, blue; all remaining atoms including the polar backbone, white). The close-up view of the substrate binding pocket illustrating the hydrophobicity along with the hydrophobic residues (Val34, Thr36, Pro60, Val65, Tyr122, Leu196 and Leu227) in direct contact with **9** (stick, pale green). **b**, ConSurf[45,46] map showing the conservation of residues around the kvPepI active site. The calculation was performed on a sample of 150 homologous sequences selected using default settings. F_{420} (orange) along with the modelled **9** (pale green) are shown as sticks. **c**, LigPlot^{+} diagram

of the interactions between the modelled **9** and kvPepI – F_{420}. The dashed lines show distances in Å. **d**, Structural analysis of kvPepI^{Y122A} – F_{420} cocrystal structure. Superposition of kvPepI – F_{420} with kvPepI^{Y122A} – F_{420} complex structure. Cα RMSD was approx. 0.12 Å (kvPepI^{Y122A} – F_{420} vs. kvPepI - F_{420}; left) over the entire length of the protein. Polder map (grey isomesh; contoured at 3σ) of F_{420} bound to kvPepI^{Y122A} is shown on the right.

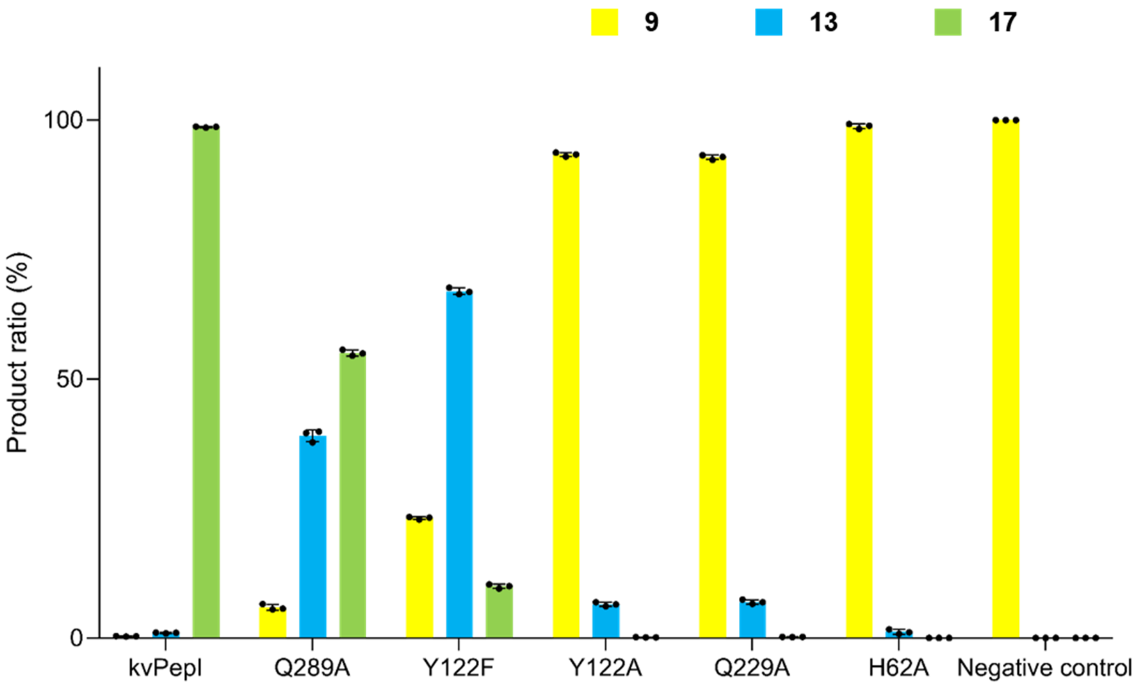

Supplementary Fig. 24. Evaluation of the activity of kvPepI and its mutants on two reduction steps was conducted using HPLC-MS analysis to determine the compound ratio between substrate **9** and products **13** and **17**. Reactions with heat-inactivated enzymes served as controls. Bar heights represent the average compound ratios from three independent reactions, with all data points presented. Three biological replicates (n=3 independent experiments) were performed. All error bars represent the standard error of the mean (SEM). Source data are provided as a Source Data file.

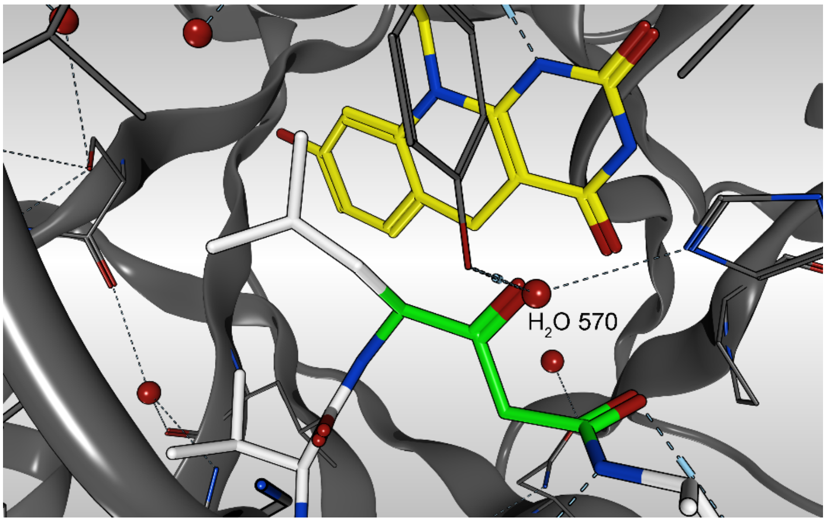

Supplementary Fig. 25. Initial orientation of β-keto amide motif of PreSta3 (green carbons) of compound **9** (white carbons) in the active site in the modelling process. Carbon atoms of cofactor F_{420} are shown in yellow. Ribbon and sidechain atoms of kvPepI protein are shown in dark grey. Oxygen: red. Nitrogen: blue. Hydron is left out for clarity. Dotted lines indicate hydrogen bonds.

NMR spectra

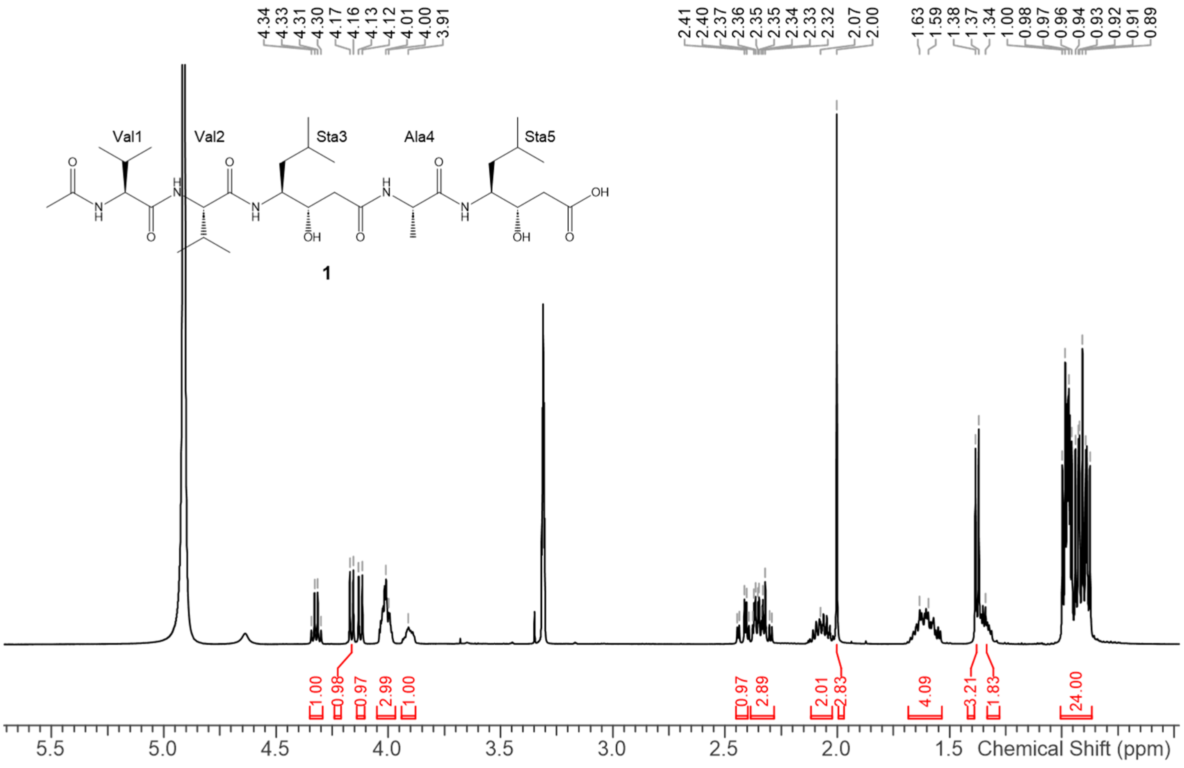

Supplementary Fig. 26. ^{1}H-NMR spectrum of compound **1** (500 MHz, methanol-d_4).

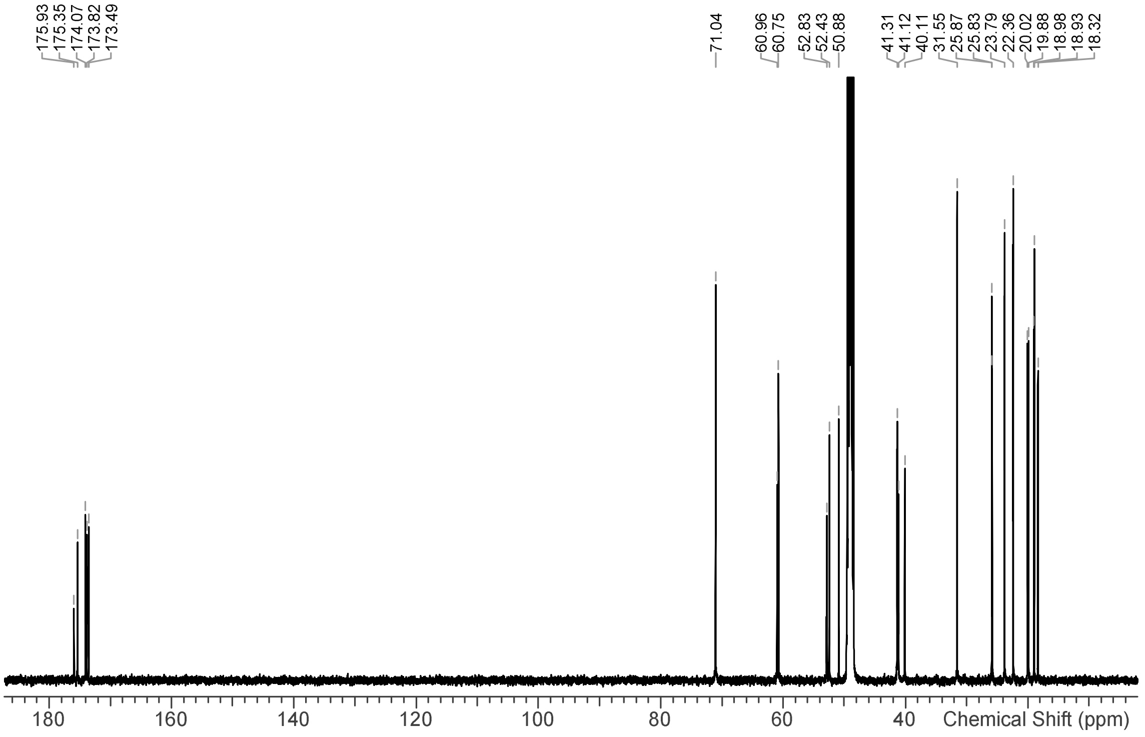

Supplementary Fig. 27. ^{13}C -NMR spectrum of compound **1** (125 MHz, methanol-d_4).

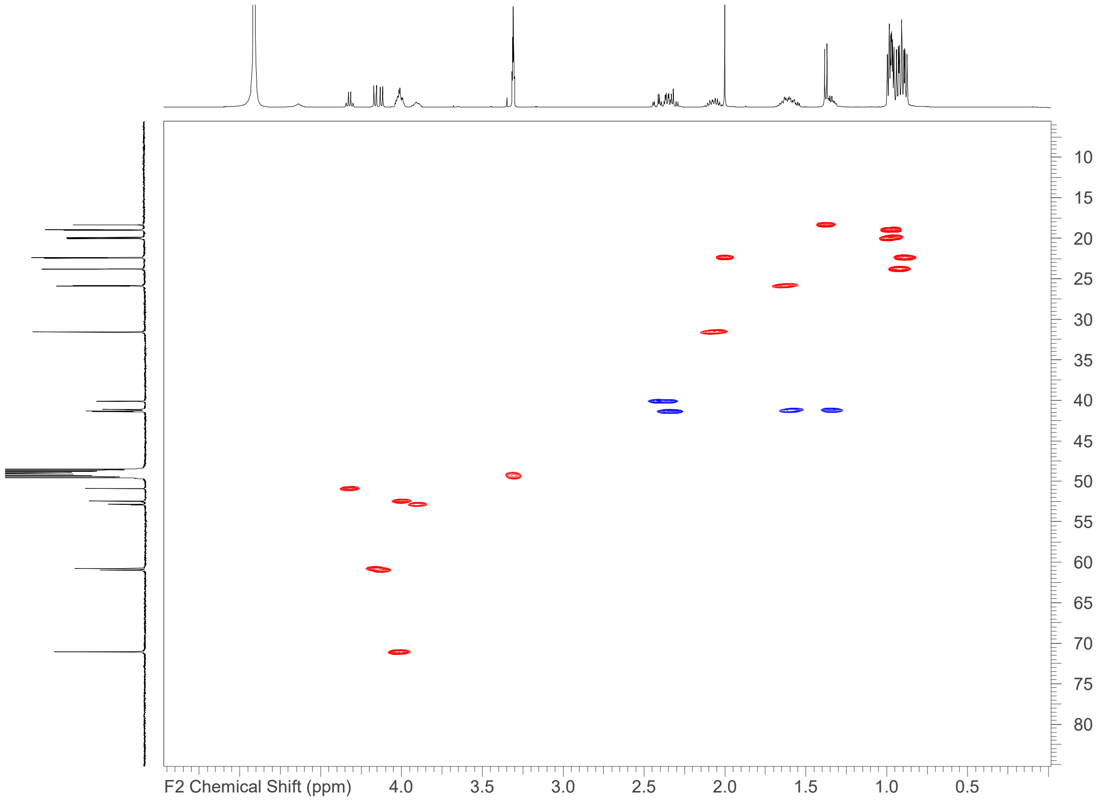

Supplementary Fig. 28. ^{1}H,^{13}C-HSQC spectrum of compound **1** (^{1}H:500 MHz, ^{13}C:125 MHz, methanol-d_4).

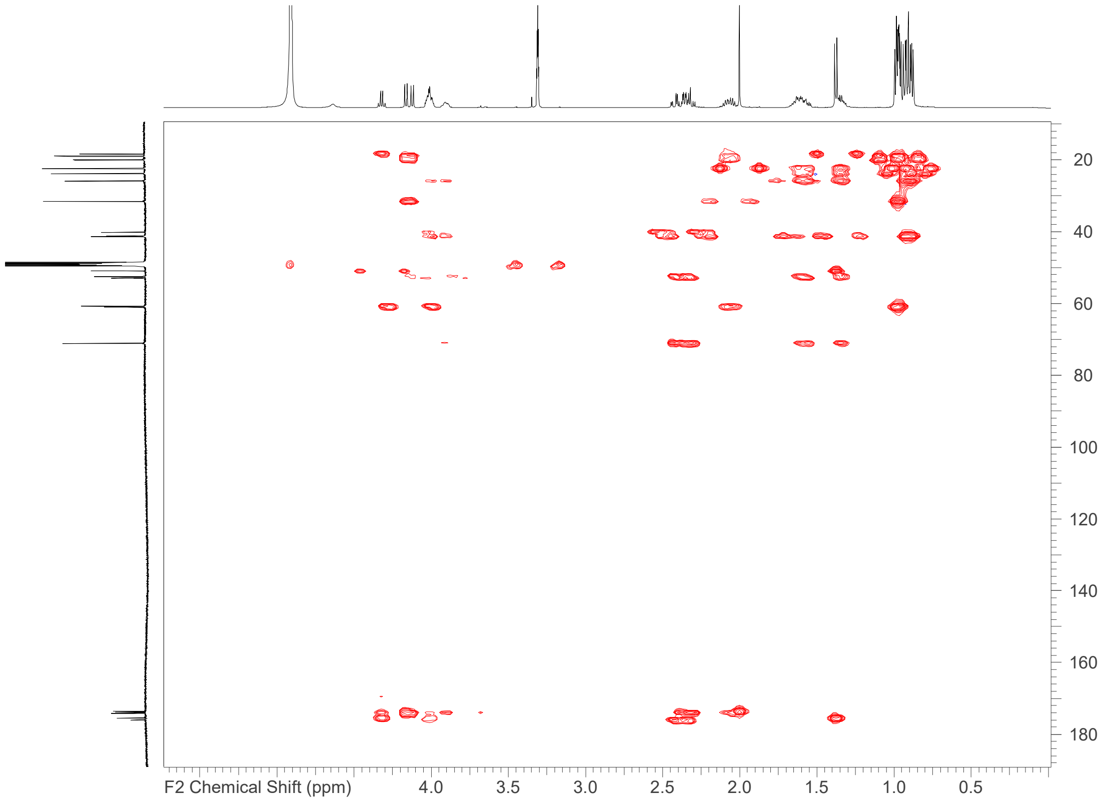

Supplementary Fig. 29. ^{1}H,^{13}C-HMBC spectrum of compound **1** (^{1}H:500 MHz, ^{13}C:125 MHz, methanol-d_4).

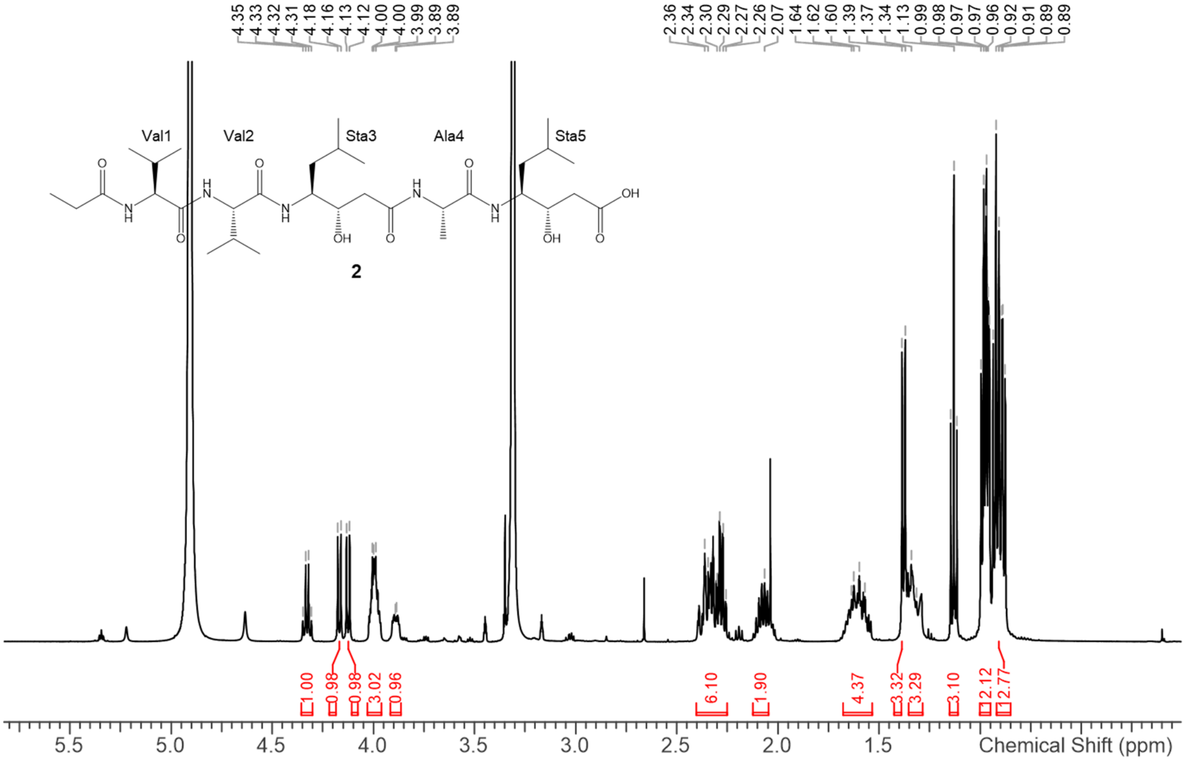

Supplementary Fig. 30. ^{1}H-NMR spectrum of compound **2** (500 MHz, methanol-d_4).

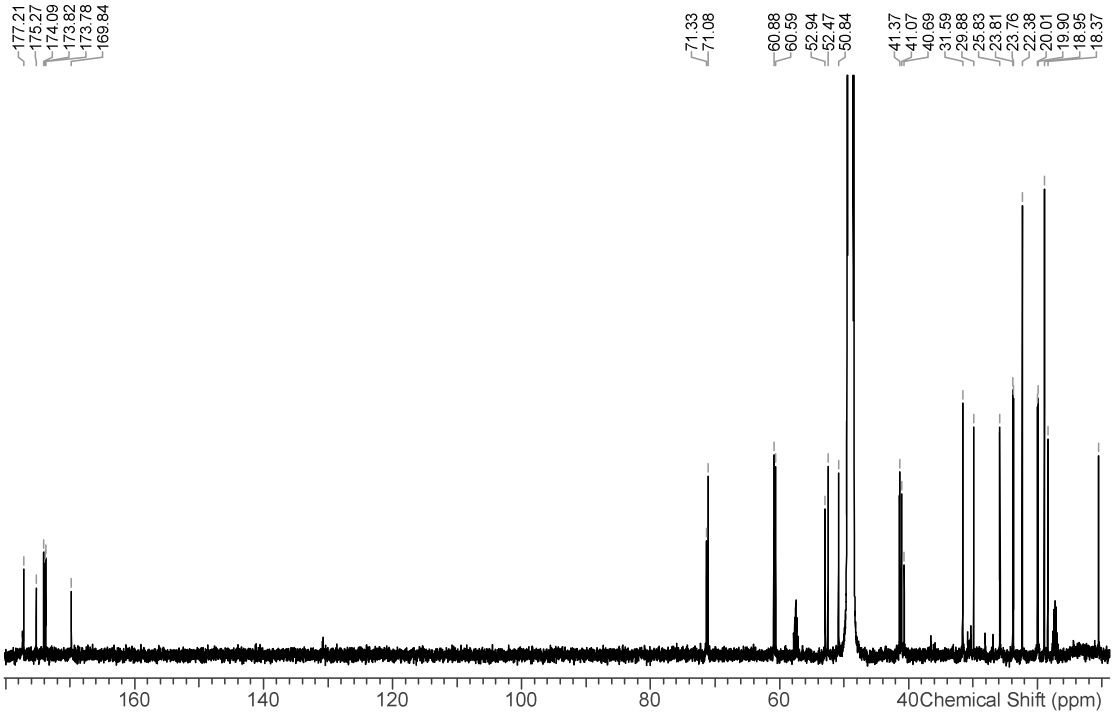

Supplementary Fig. 31. ^{13}C-NMR spectrum of compound **2** (125 MHz, methanol-d_4).

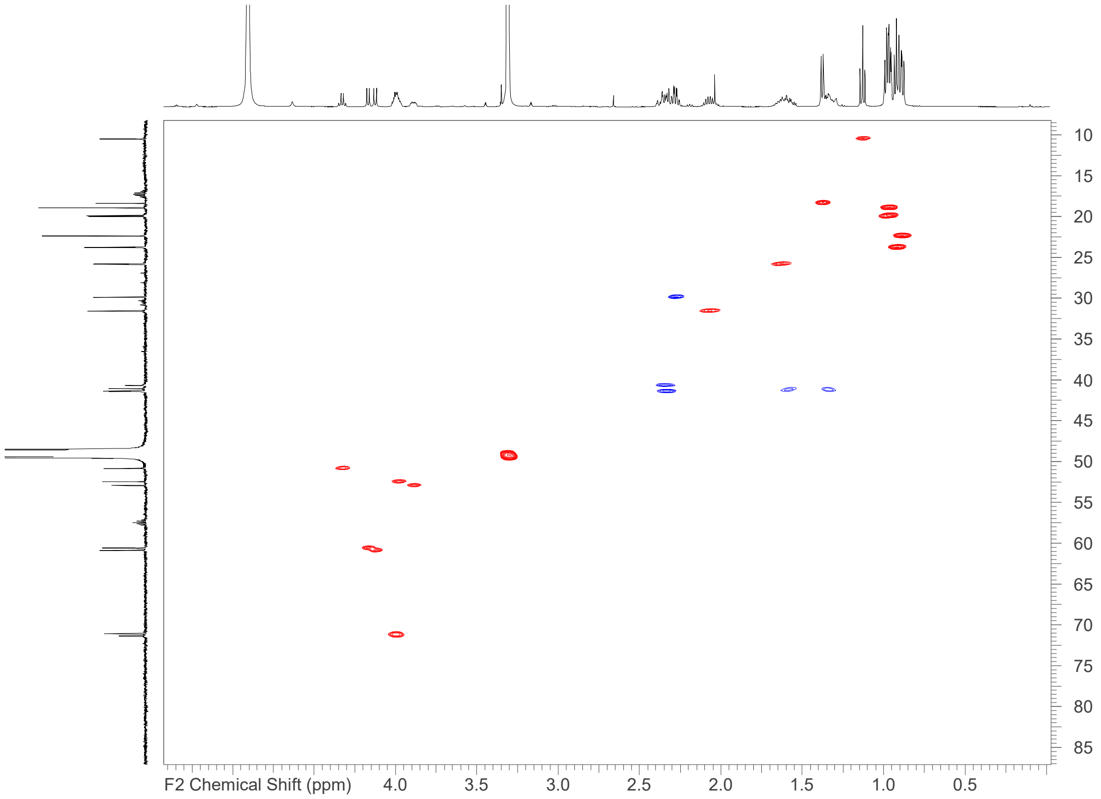

Supplementary Fig. 32. $^{1}H,^{13}C$-HSQC spectrum of compound **2** (^{1}H:500 MHz, ^{13}C:125 MHz, methanol-d_4).

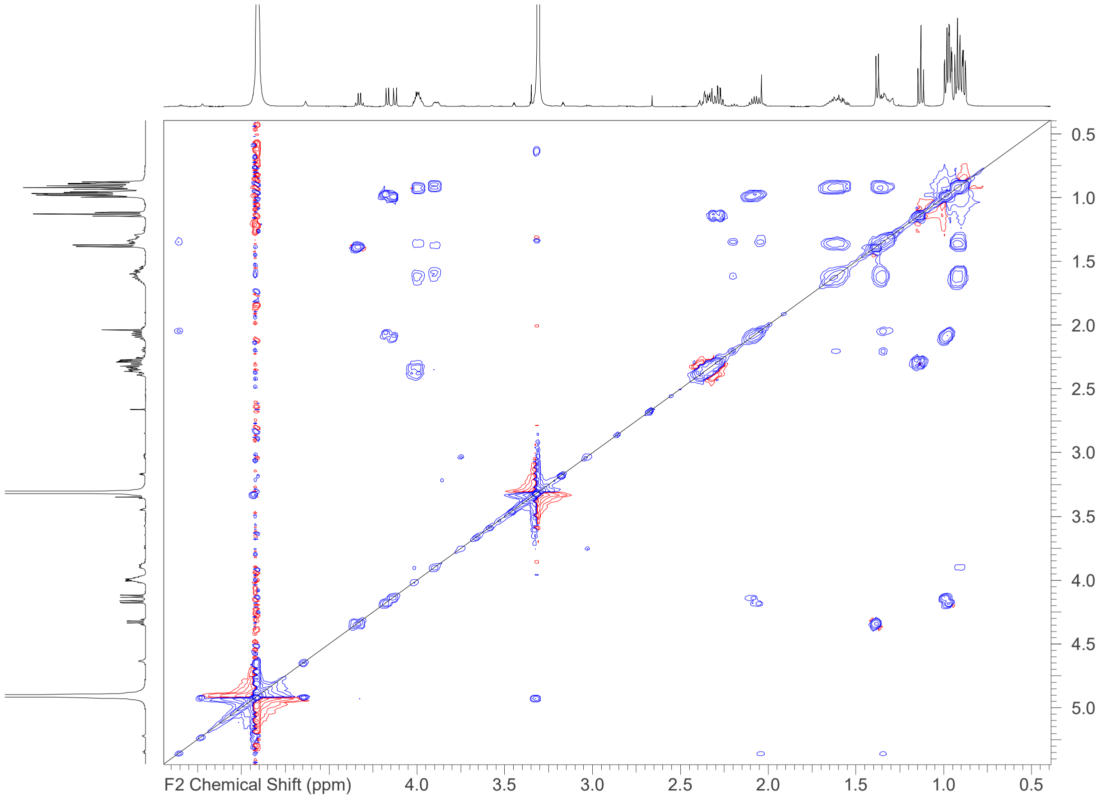

Supplementary Fig. 33. $^{1}H,^{1}H$-TCOSY spectrum of compound **2** (500 MHz, methanol- d_4).

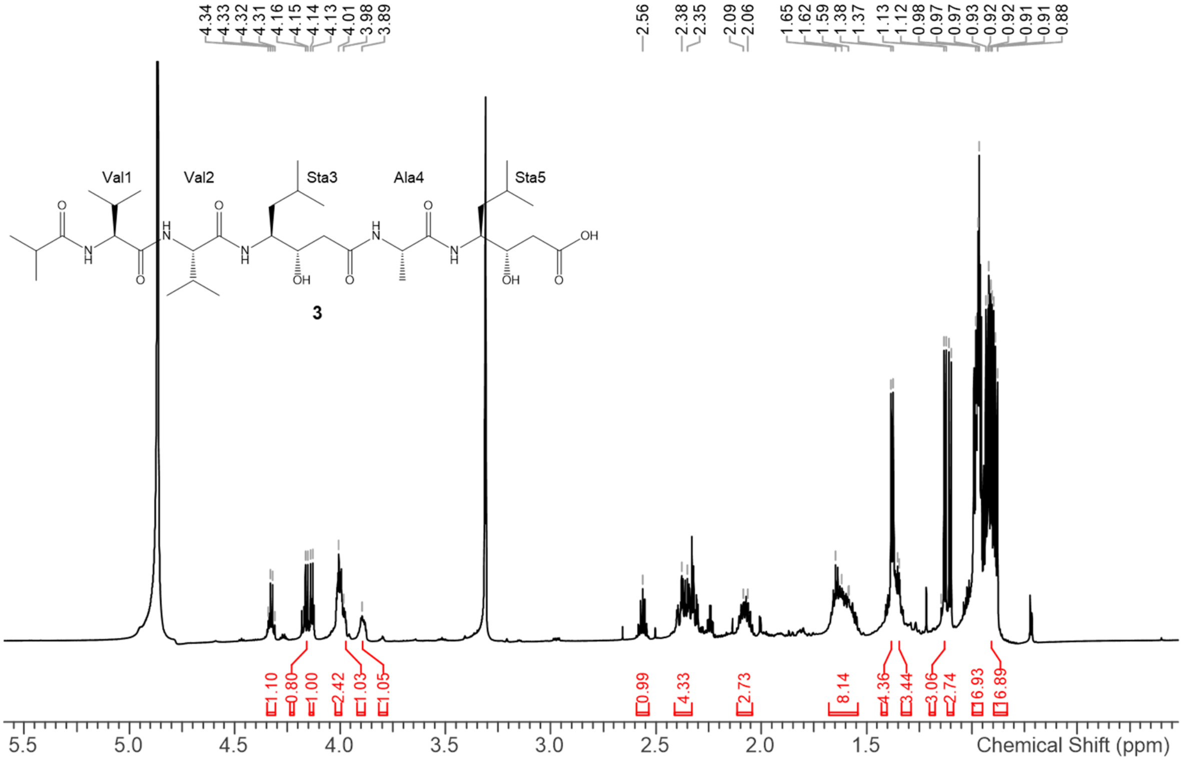

Supplementary Fig. 34. ^{1}H-NMR spectrum of compound **3** (700 MHz, methanol-d_4).

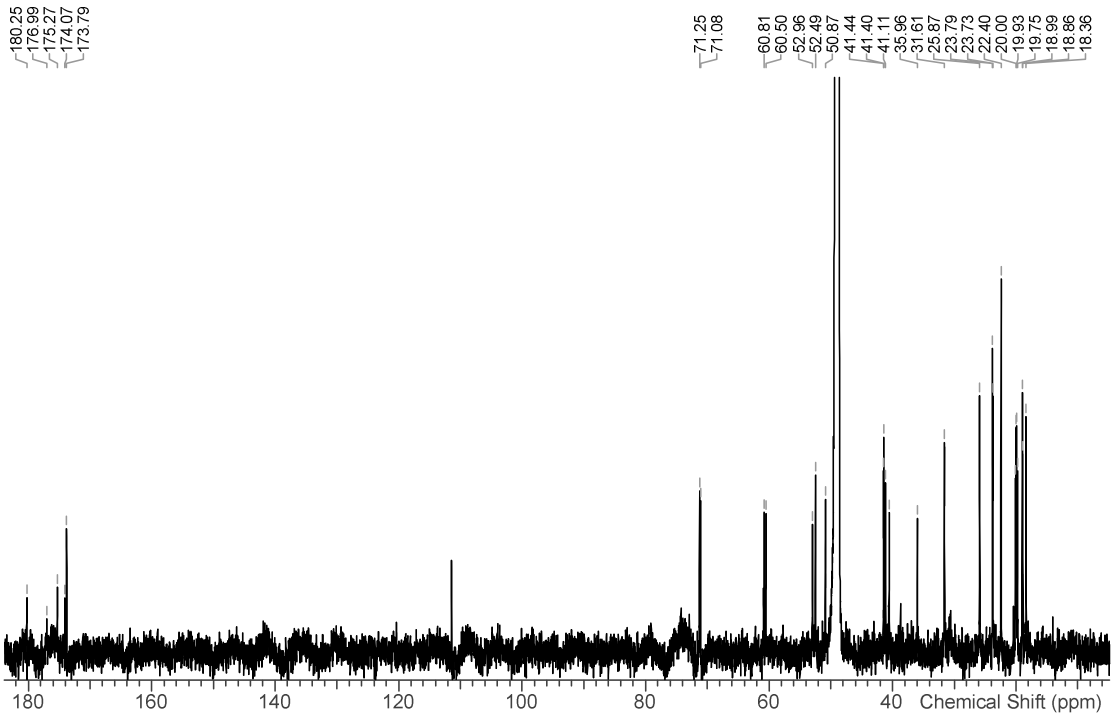

Supplementary Fig. 35. ^{13}C-NMR spectrum of compound **3** (175 MHz, methanol-d_4).

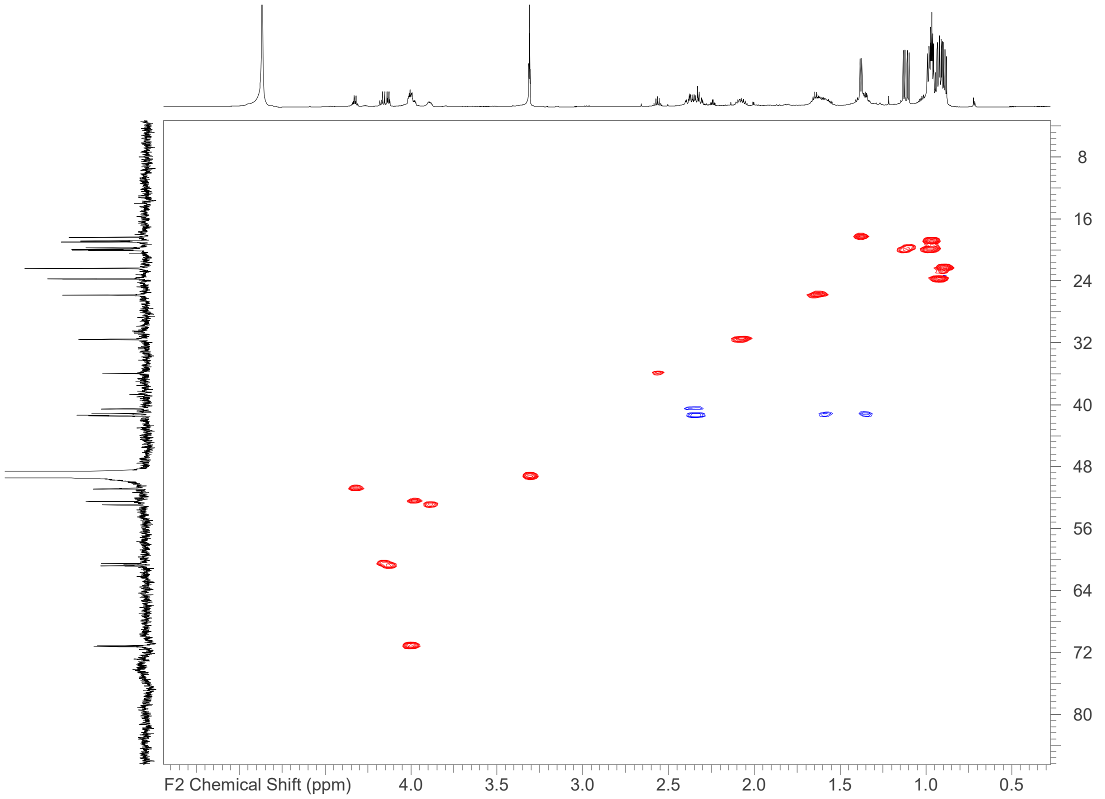

Supplementary Fig. 36. ^{1}H,^{13}C-HSQC spectrum of compound **3** (^{1}H:700 MHz, ^{13}C:175 MHz, methanol-d_4).

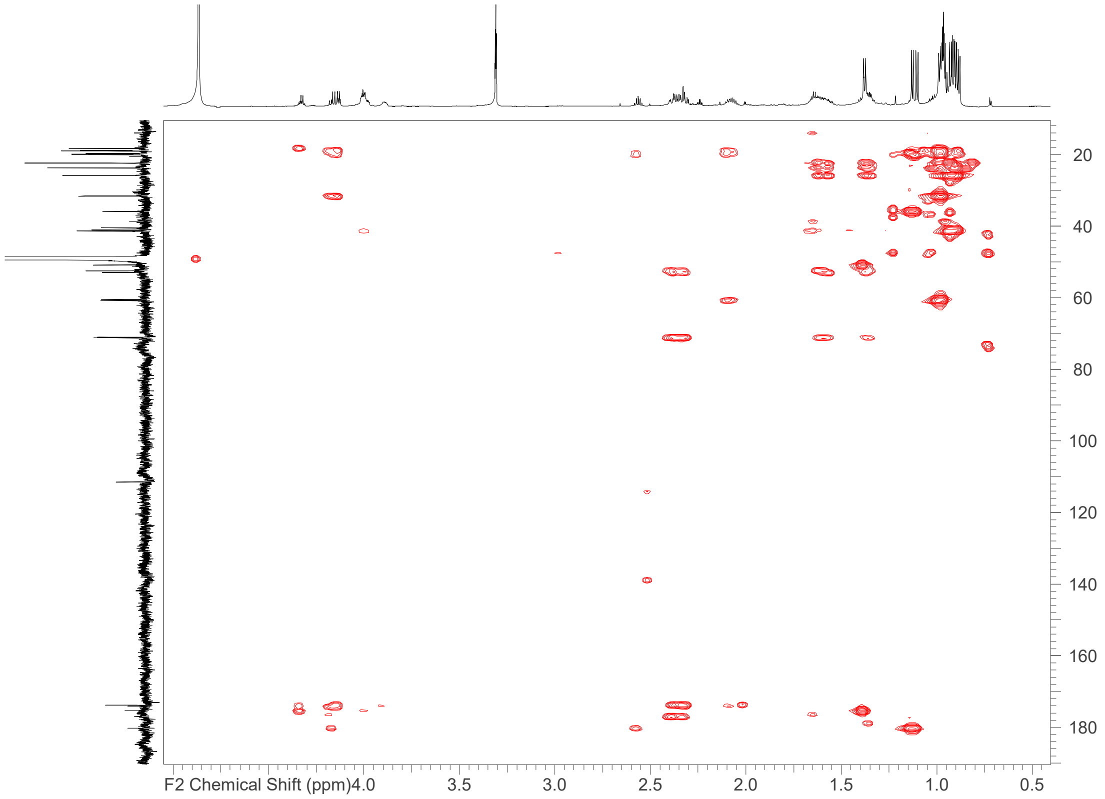

Supplementary Fig. 37. ^{1}H,^{1}H-HMBC spectrum of compound **3** (^{1}H:700 MHz, ^{13}C:175 MHz, methanol-d_4).

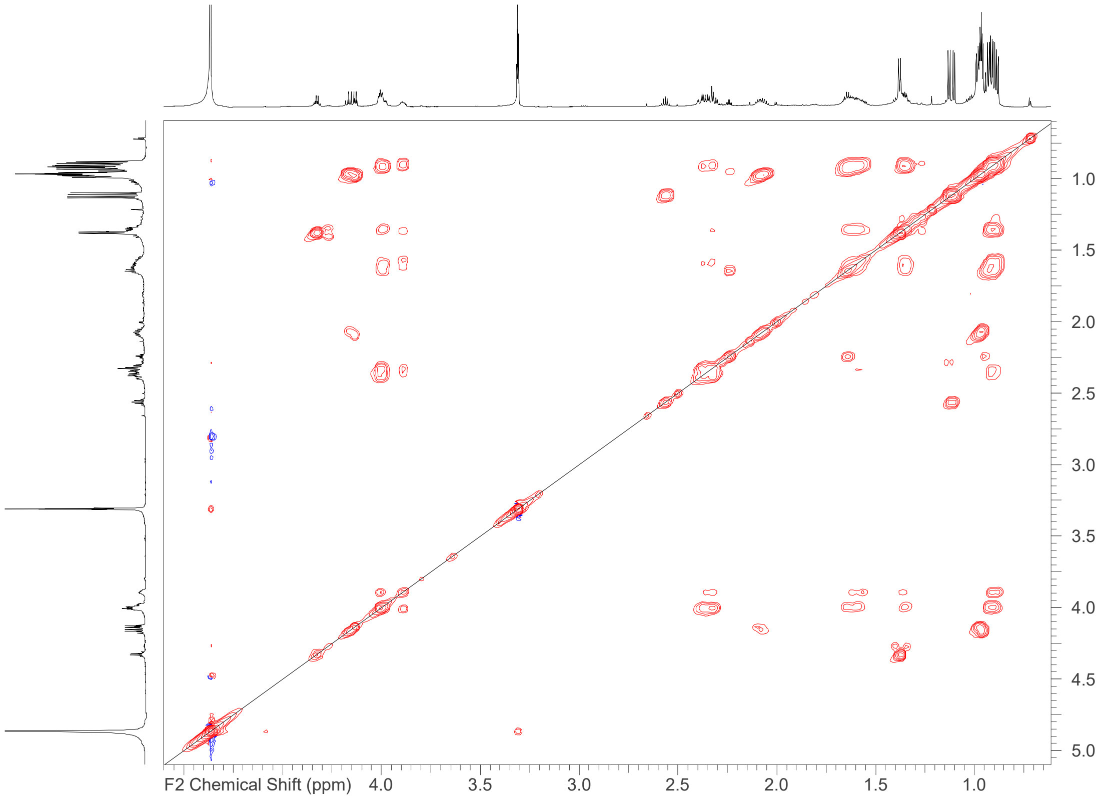

Supplementary Fig. 38. ^{1}H,^{1}H-TCOSY spectrum of compound **3** (700 MHz, methanol-d_4).

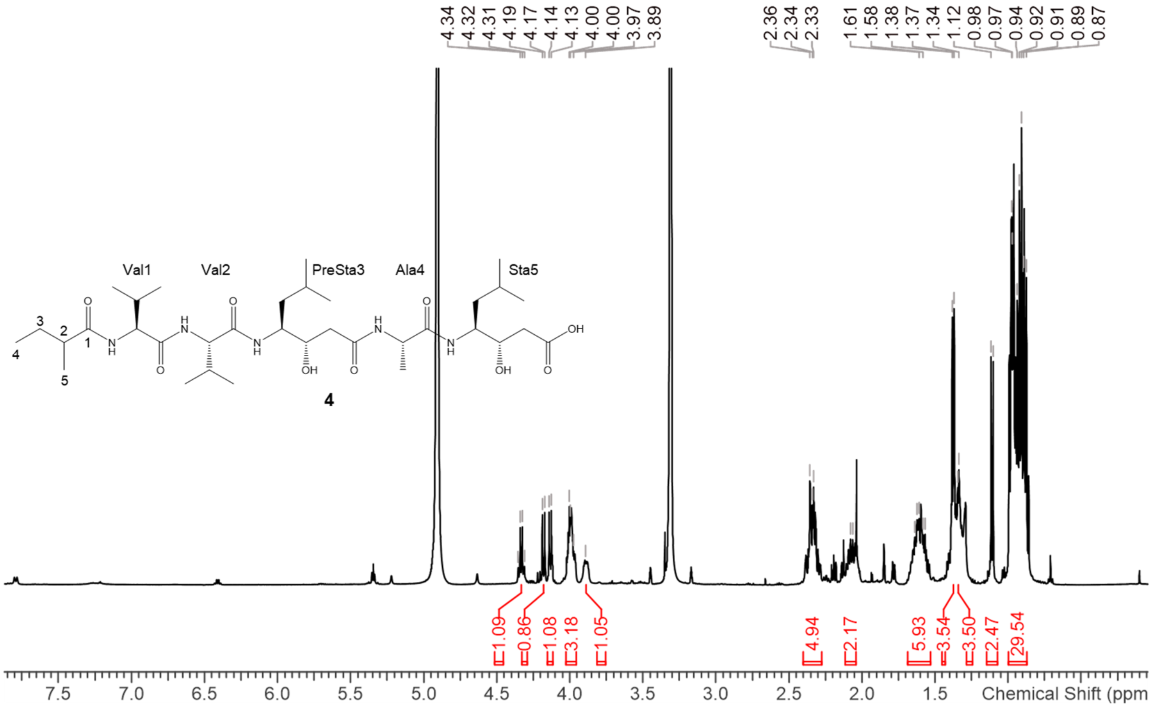

Supplementary Fig. 39. ^{1}H-NMR spectrum of compound **4** (500 MHz, methanol-d_4).

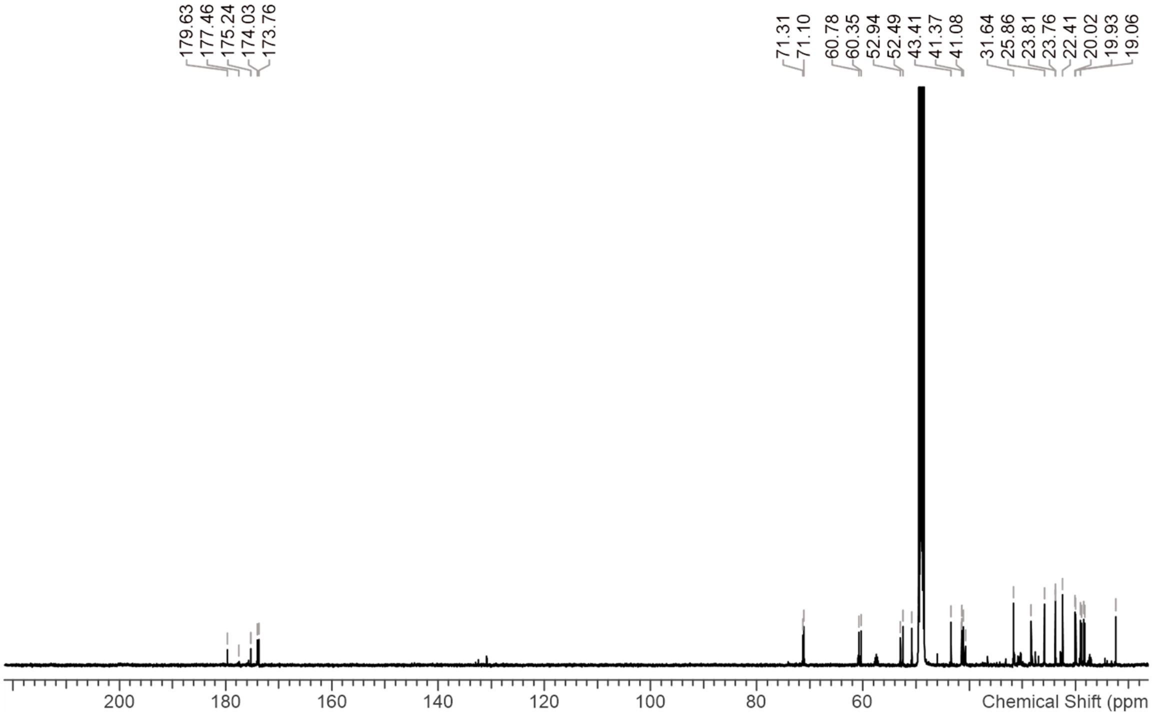

Supplementary Fig. 40. ^{13}C-NMR spectrum of compound **4** (125 MHz, methanol-d_4).

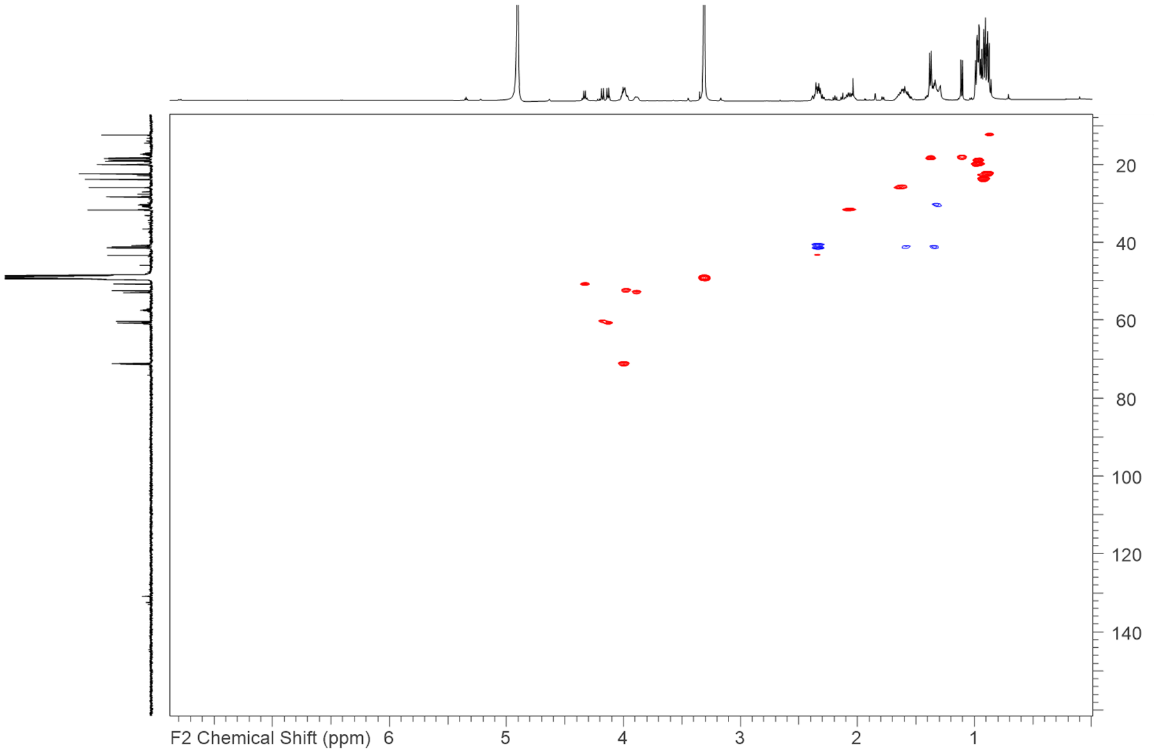

Supplementary Fig. 41. ^{1}H,^{13}C-HSQC spectrum of compound **4** (^{1}H:500 MHz, ^{13}C:125 MHz, methanol-d_4).

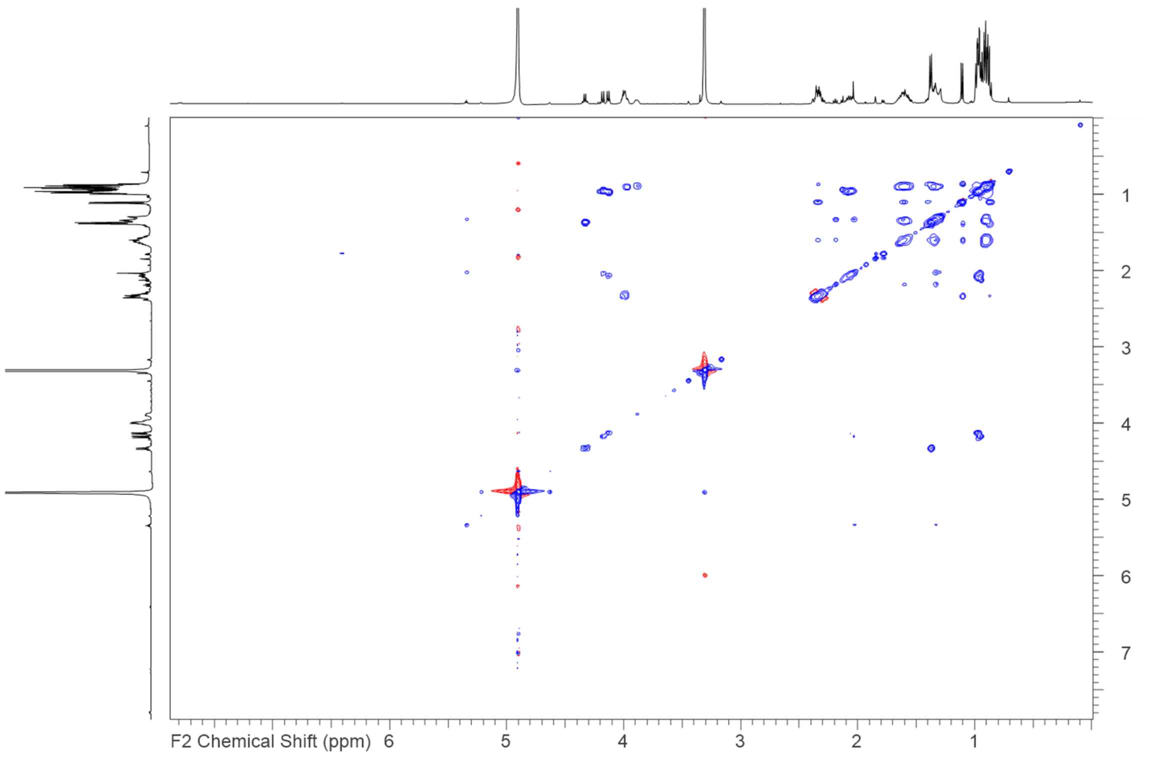

Supplementary Fig. 42. $^{1}H,^{1}H$-TCOSY spectrum of compound **4** (500 MHz, methanol-d_4).

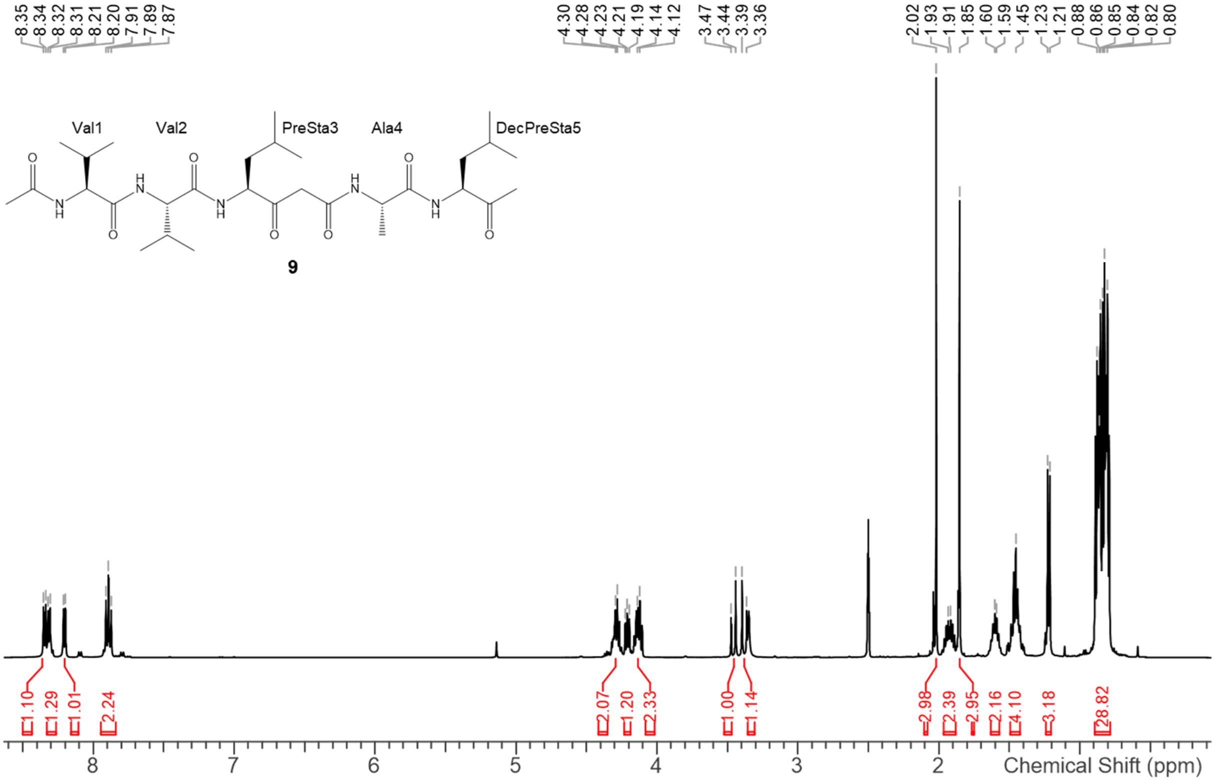

Supplementary Fig. 43. ^{1}H-NMR spectrum of compound **9** (500 MHz, DMSO-d_6).

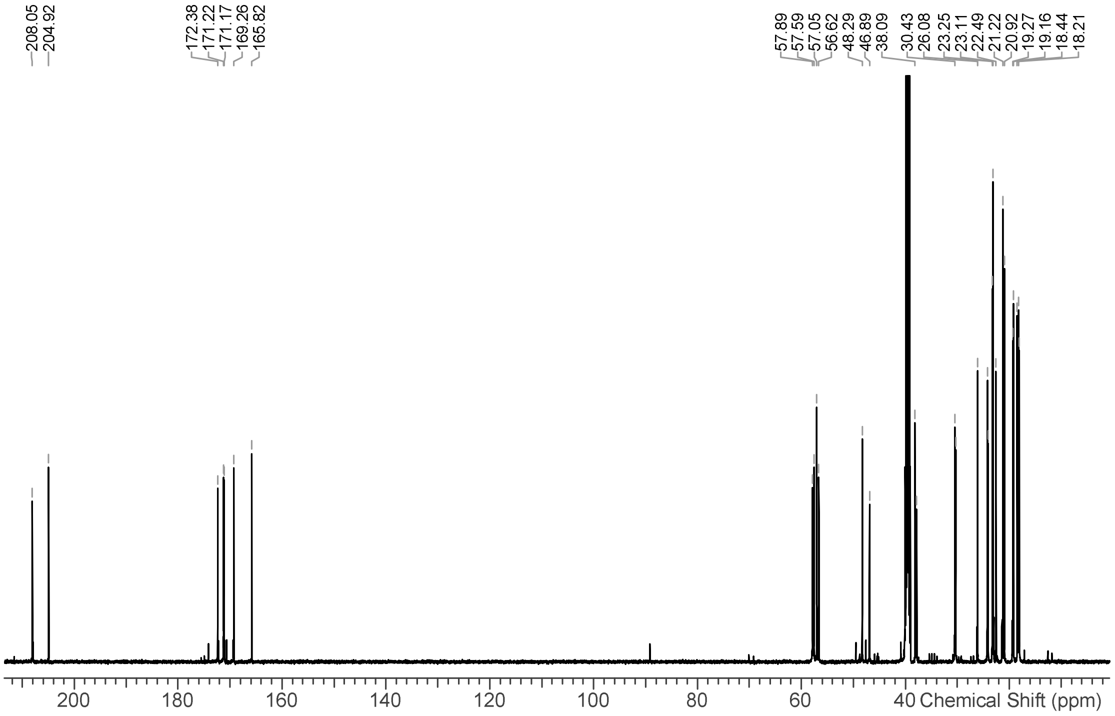

Supplementary Fig. 44. ^{13}C-NMR spectrum of compound **9** (125 MHz, DMSO-d_6).

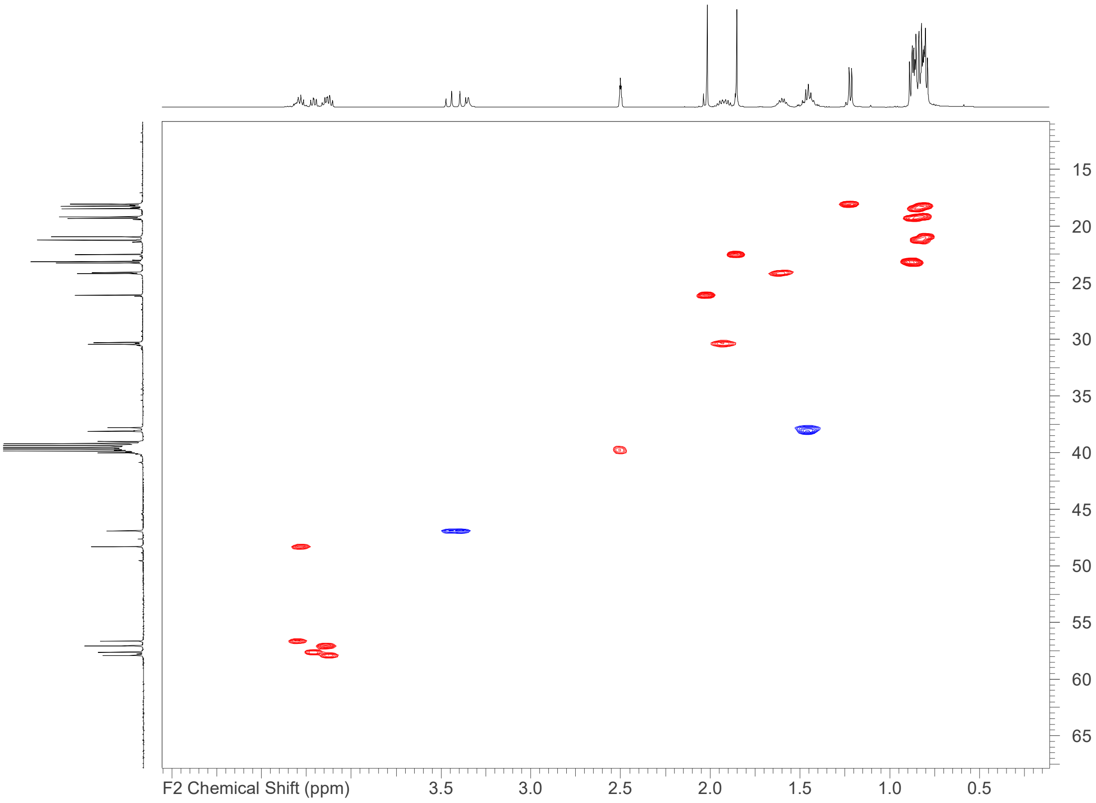

Supplementary Fig. 45. ^{1}H,^{13}C-HSQC spectrum of compound **9** (^{1}H:500 MHz, ^{13}C:125 MHz, DMSO-d_6).

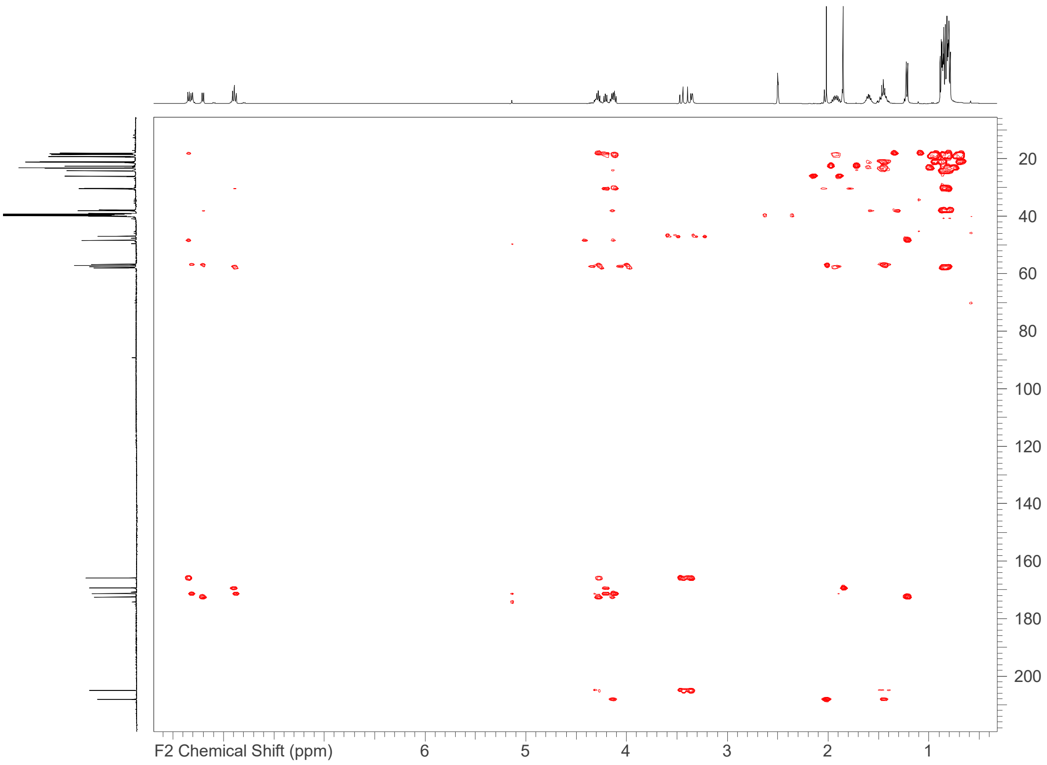

Supplementary Fig. 46. ^{1}H,^{13}C-HMBC spectrum of compound **9** (^{1}H:500 MHz, ^{13}C:125 MHz, DMSO-d_6).

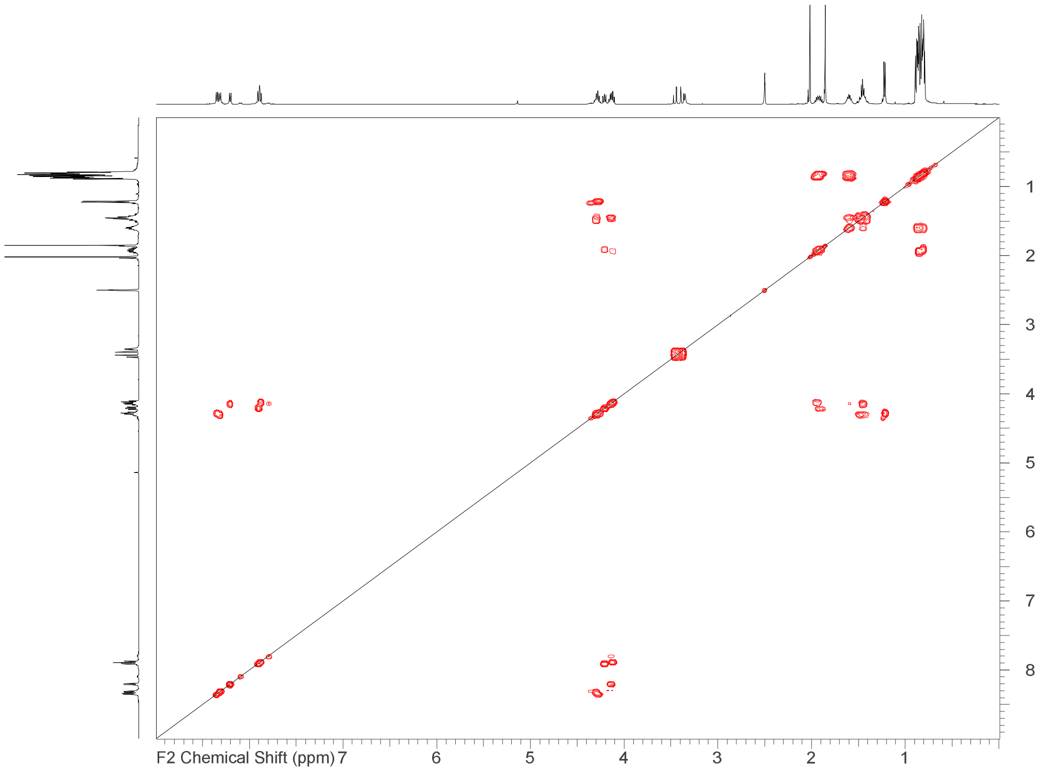

Supplementary Fig. 47. ^{1}H,^{1}H-COSY spectrum of compound **9** (500 MHz, DMSO-d_6).

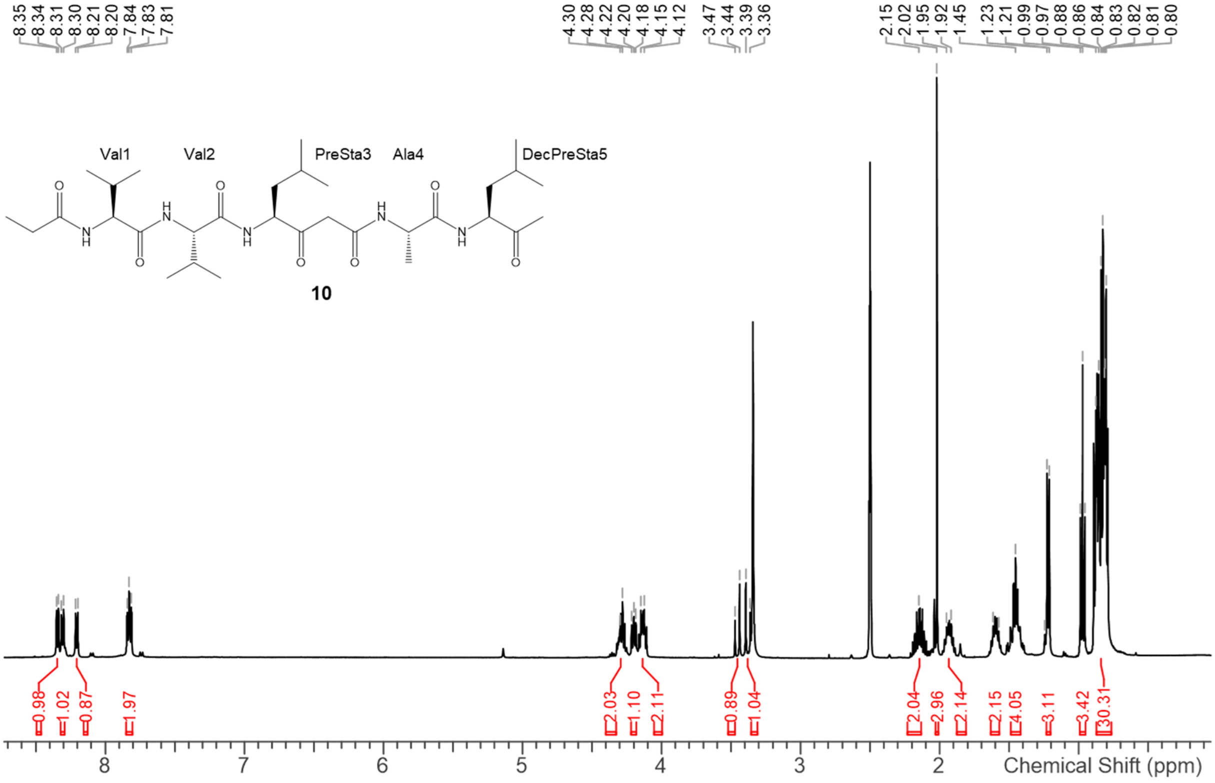

Supplementary Fig. 48. ^{1}H-NMR spectrum of compound **10** (500 MHz, DMSO-d_6).

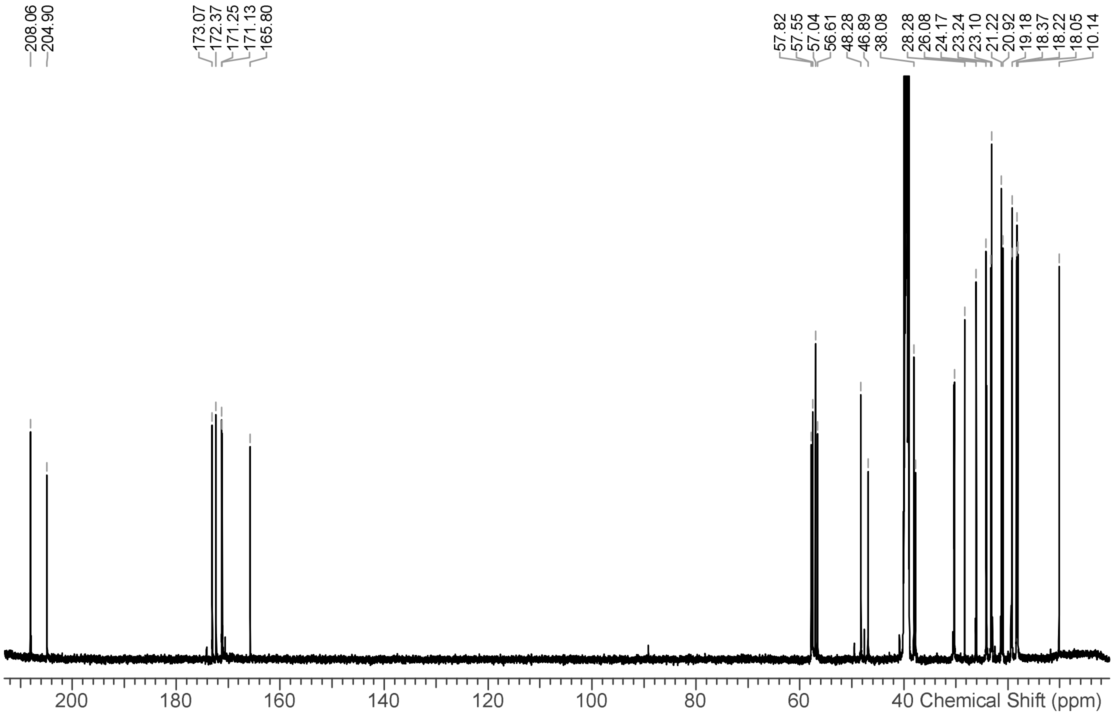

Supplementary Fig. 49. ^{13}C-NMR spectrum of compound **10** (125 MHz, DMSO-d_6).

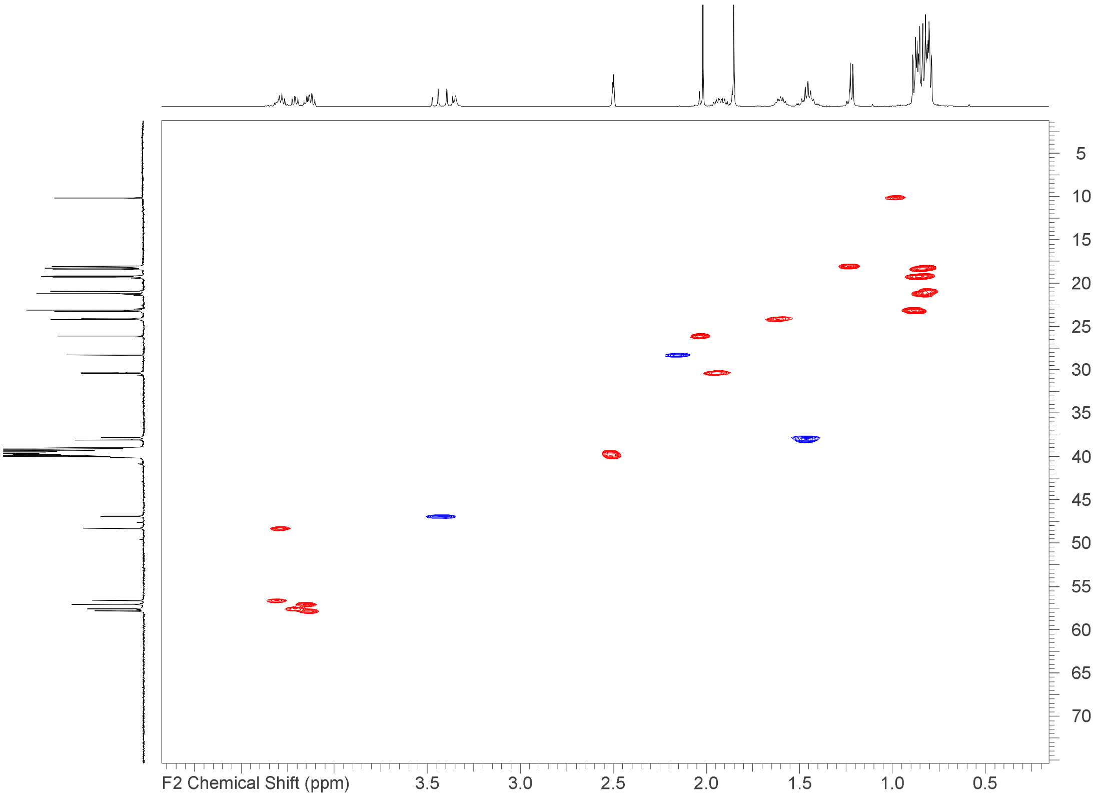

Supplementary Fig. 50. ^{1}H,^{13}C-HSQC spectrum of compound **10** (^{1}H:500 MHz, ^{13}C:125 MHz, DMSO-d_6).

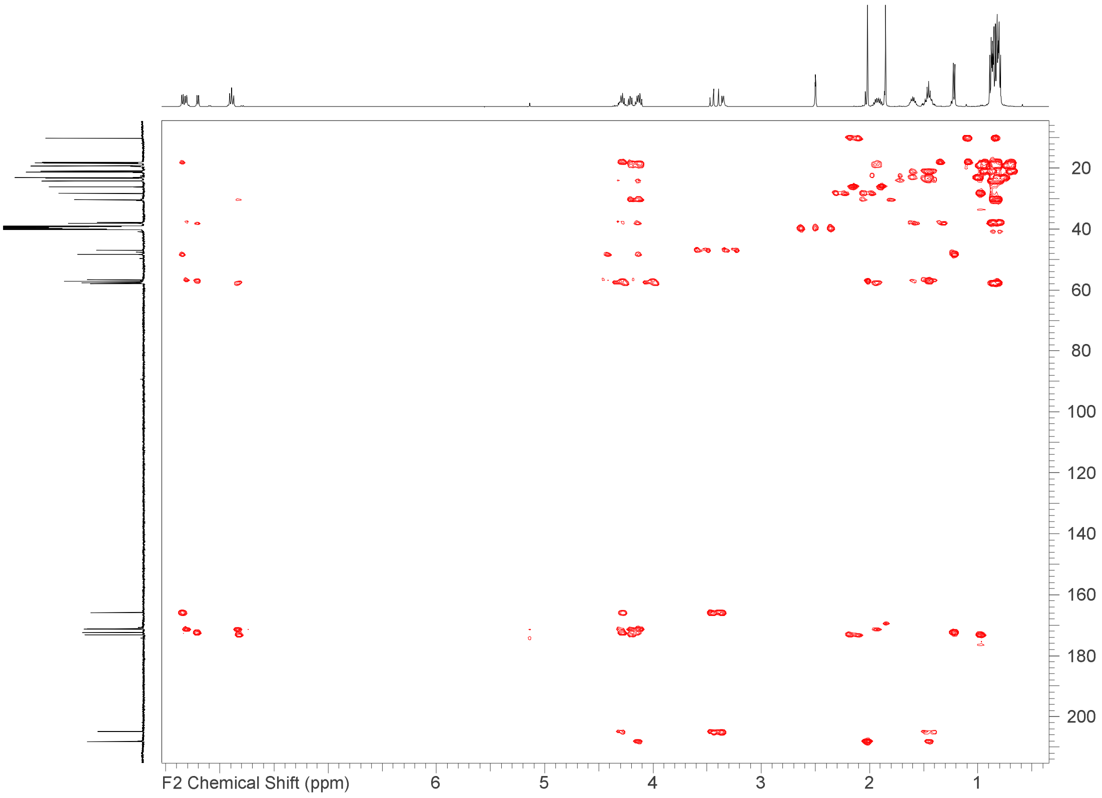

Supplementary Fig. 51. ^{1}H,^{13}C-HMBC spectrum of compound **10** (^{1}H:500 MHz, ^{13}C:125 MHz, DMSO-d_6).

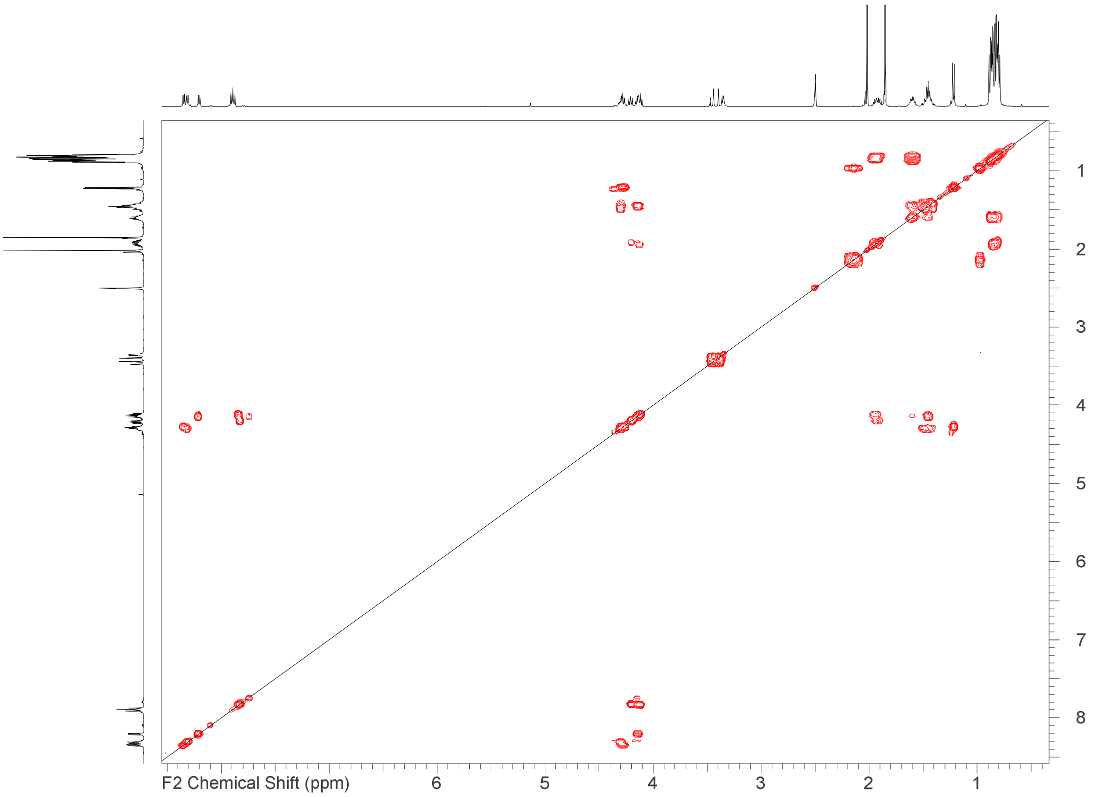

Supplementary Fig. 52. COSY spectrum of compound **10** (500 MHz, DMSO-d_6).

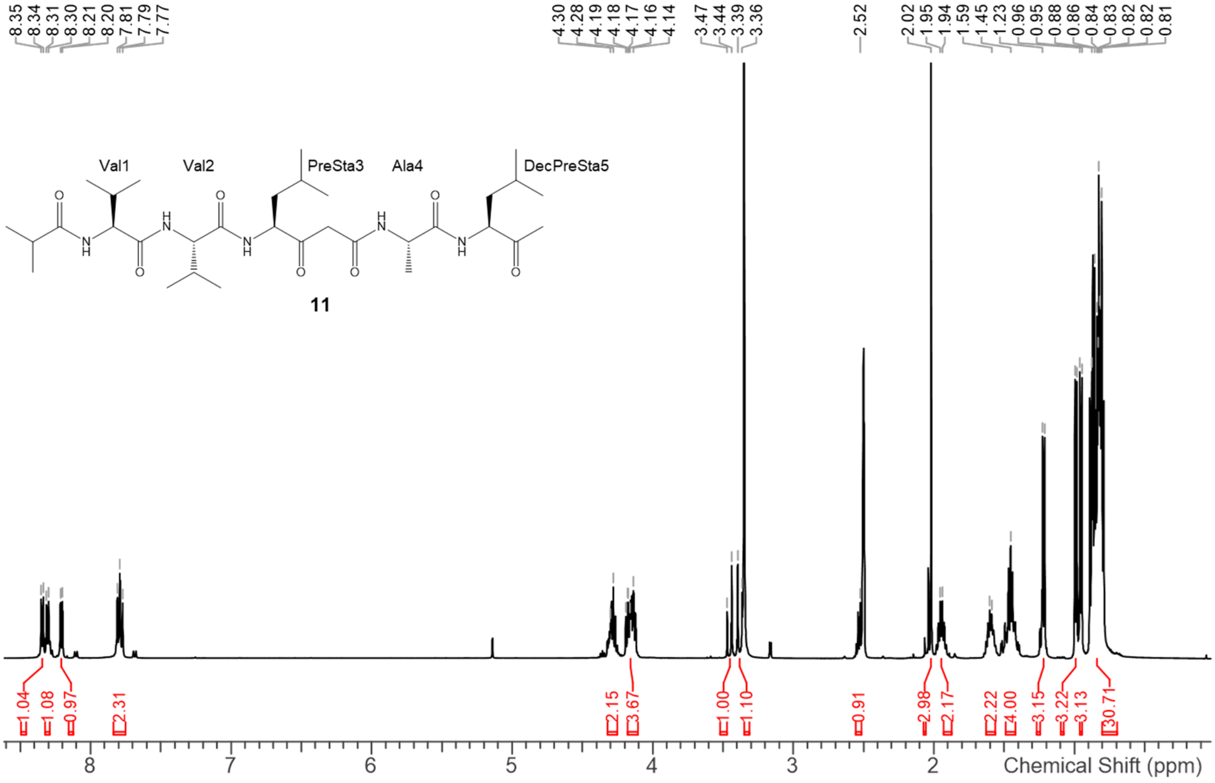

Supplementary Fig. 53. ^{1}H-NMR spectrum of compound **11** (500 MHz, DMSO-d_6).

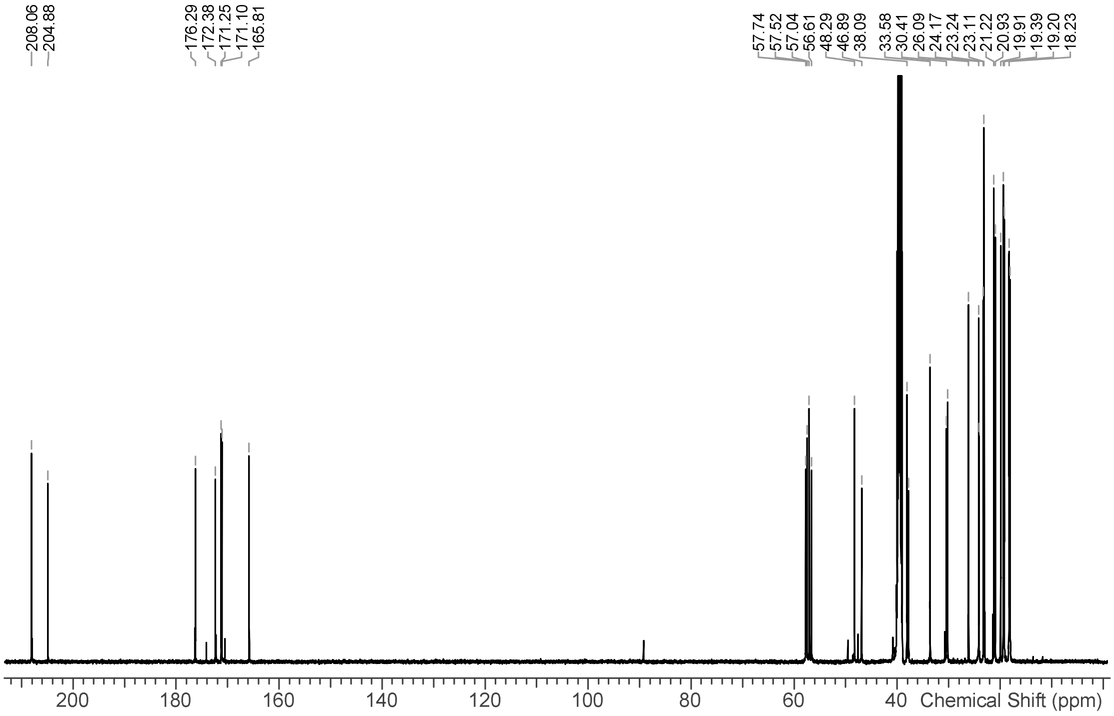

Supplementary Fig. 54. ^{13}C-NMR spectrum of compound **11** (125 MHz, DMSO-d_6).

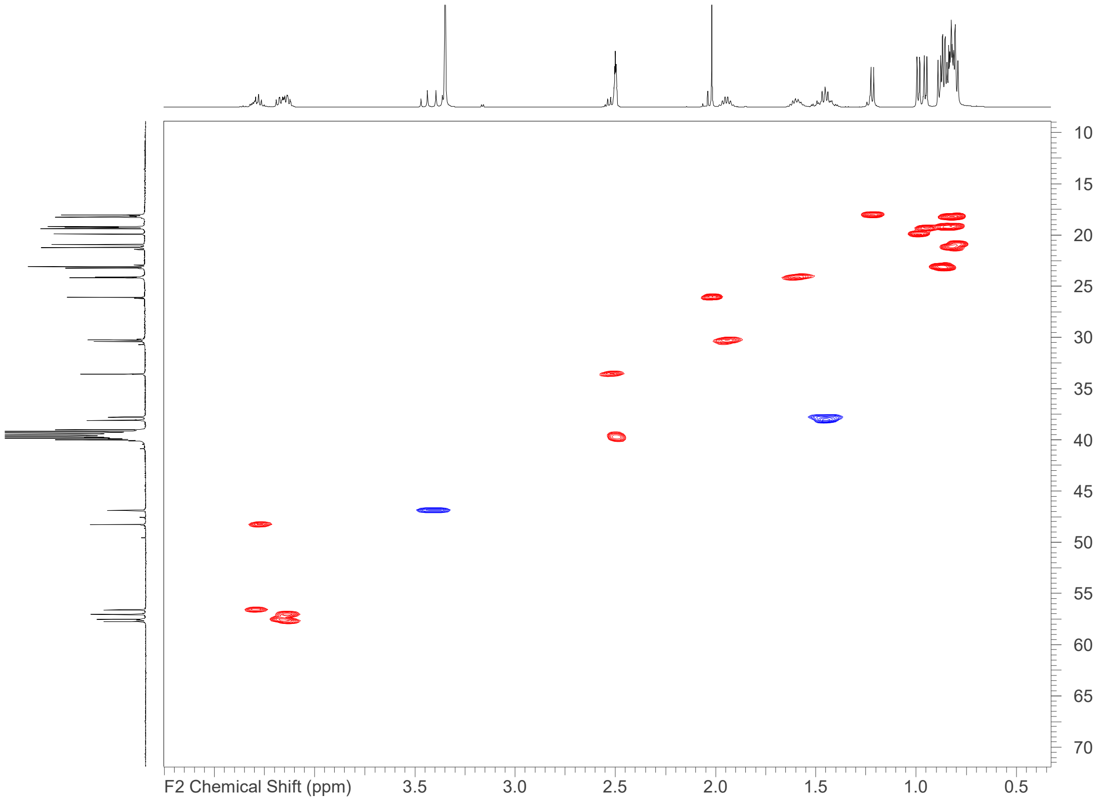

Supplementary Fig. 55. $^{1}H,^{13}C$-HSQC spectrum of compound **11** (^{1}H:500 MHz, ^{13}C:125 MHz, DMSO-d_6).

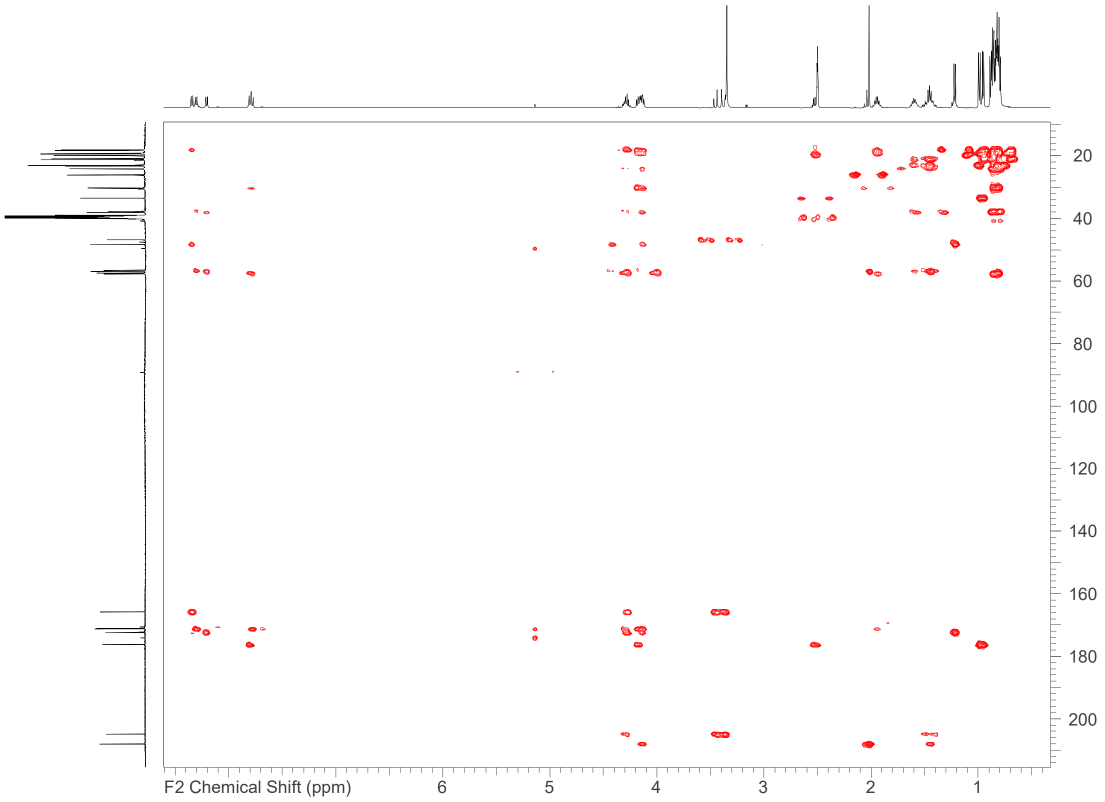

Supplementary Fig. 56. $^{1}H,^{13}C$-HMBC spectrum of compound **11** (^{1}H:500 MHz, ^{13}C:125 MHz, DMSO-d_6).

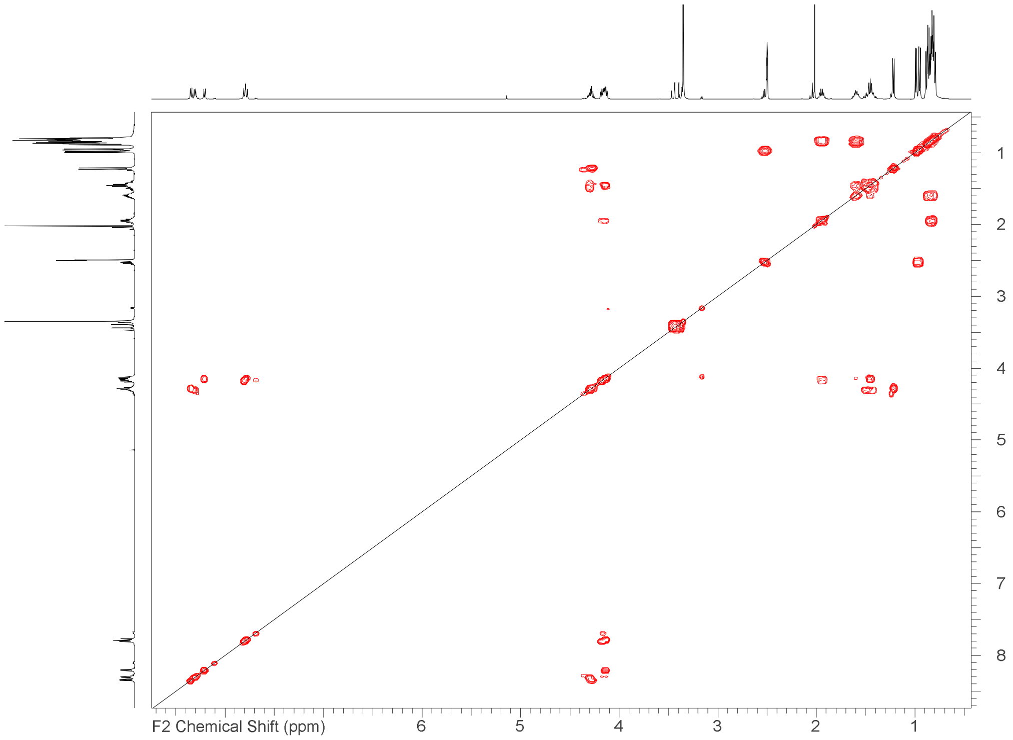

Supplementary Fig. 57. ^{1}H,^{1}H-COSY spectrum of compound **11** (500 MHz, DMSO-d_6).

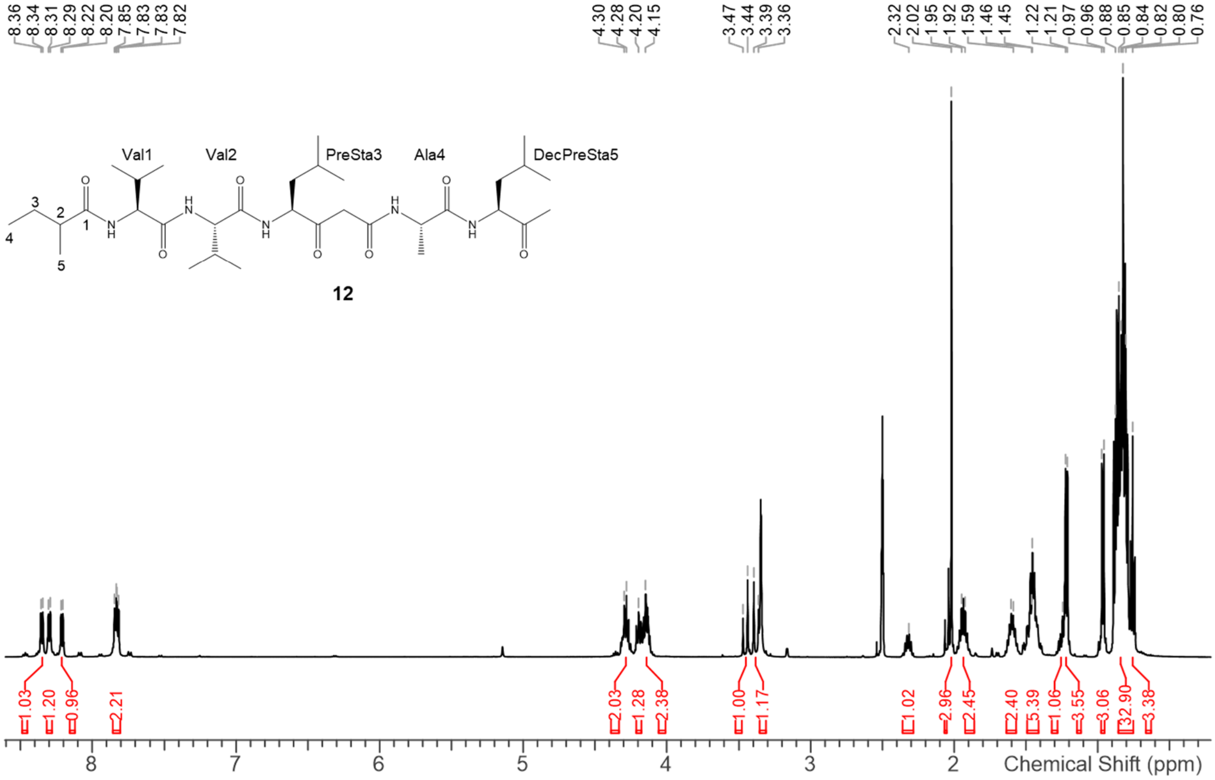

Supplementary Fig. 58. ^{1}H-NMR spectrum of compound **12** (500 MHz, DMSO-d_6).

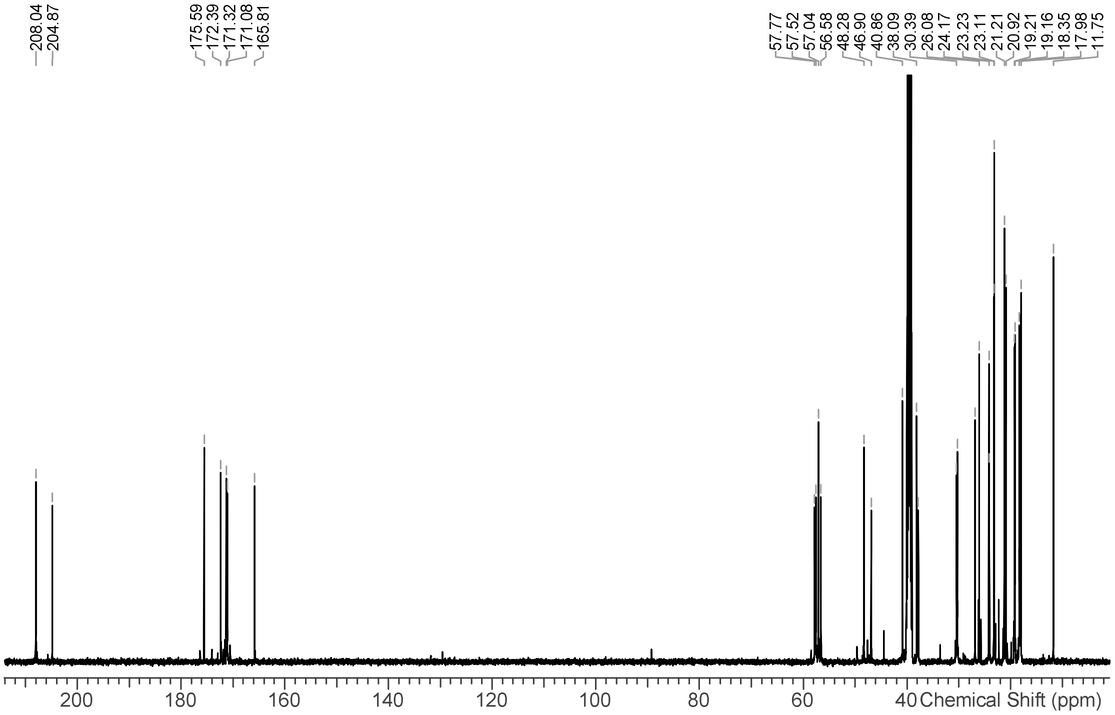

Supplementary Fig. 59. ^{13}C-NMR spectrum of compound **12** (125 MHz, DMSO-d_6).

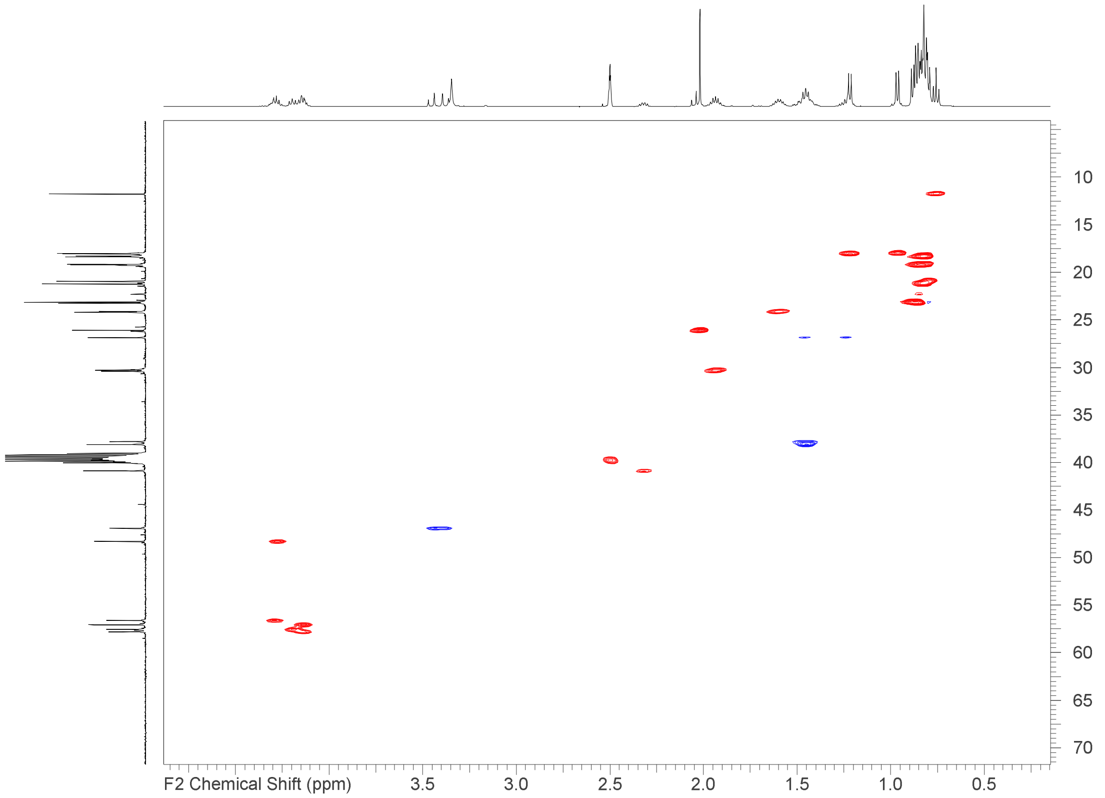

Supplementary Fig. 60. $^{1}H,^{13}C$-HSQC spectrum of compound **12** (^{1}H:500 MHz, ^{13}C:125 MHz, DMSO-d_6).

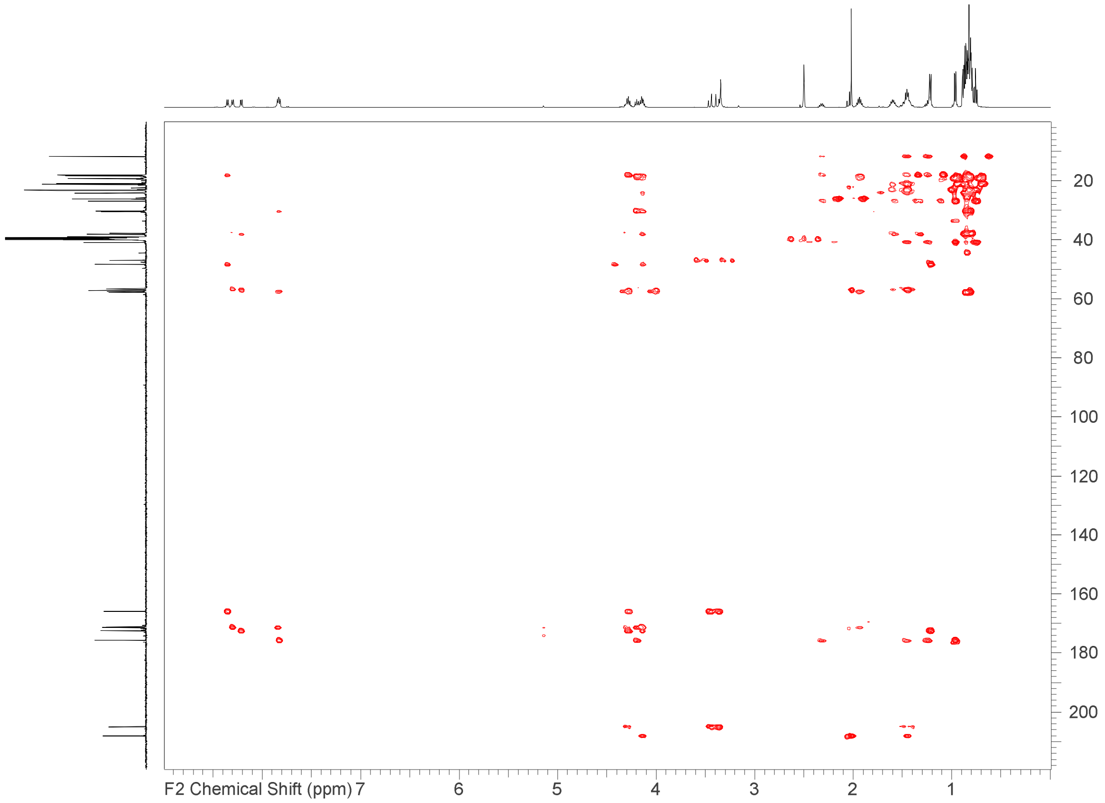

Supplementary Fig. 61. $^{1}H,^{13}C$-HMBC spectrum of compound **12** (^{1}H:500 MHz, ^{13}C:125 MHz, DMSO-d_6).

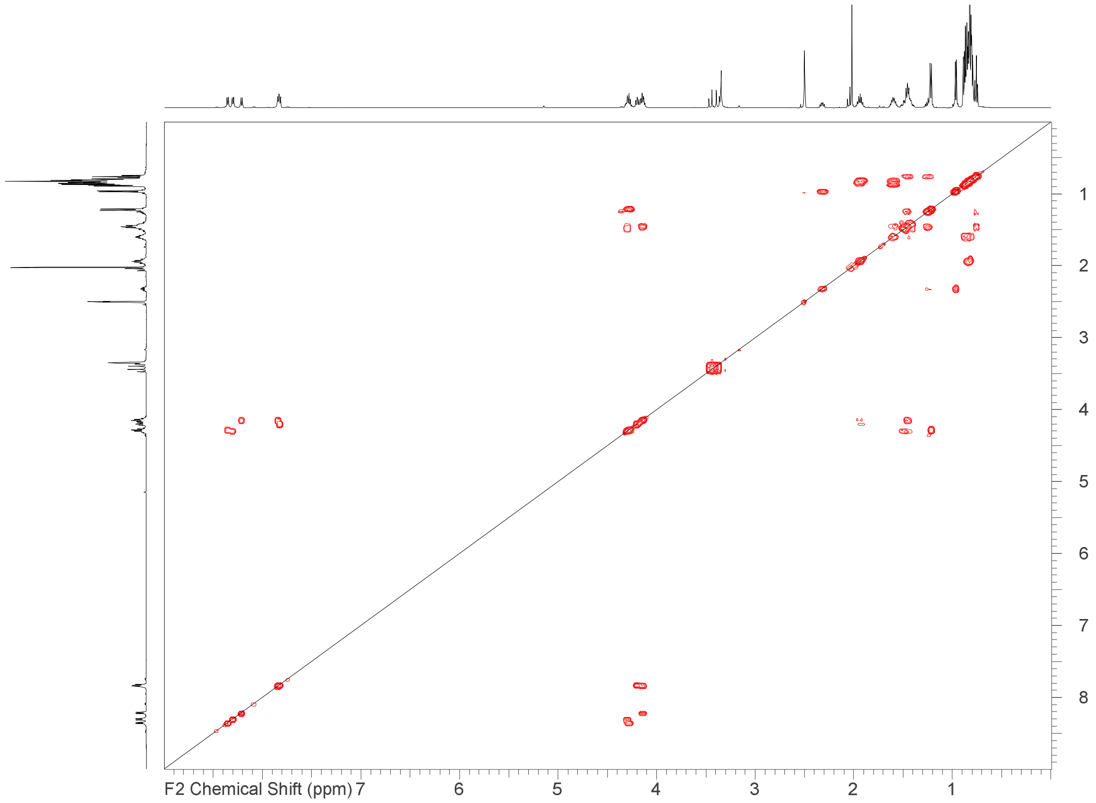

Supplementary Fig. 62. $^{1}H,^{1}H$-COSY spectrum of compound **12** (500 MHz, DMSO-d_6).

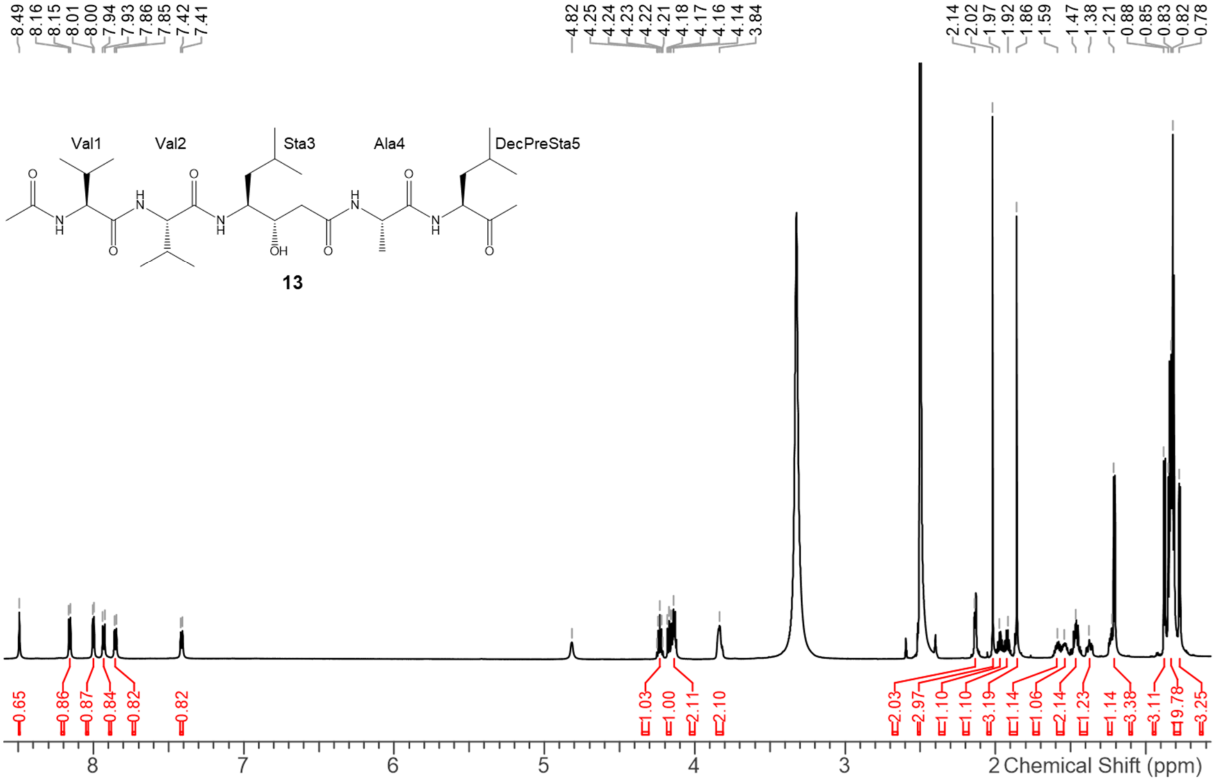

Supplementary Fig. 63. ^{1}H-NMR spectrum of compound **13** (700 MHz, DMSO-d_6).

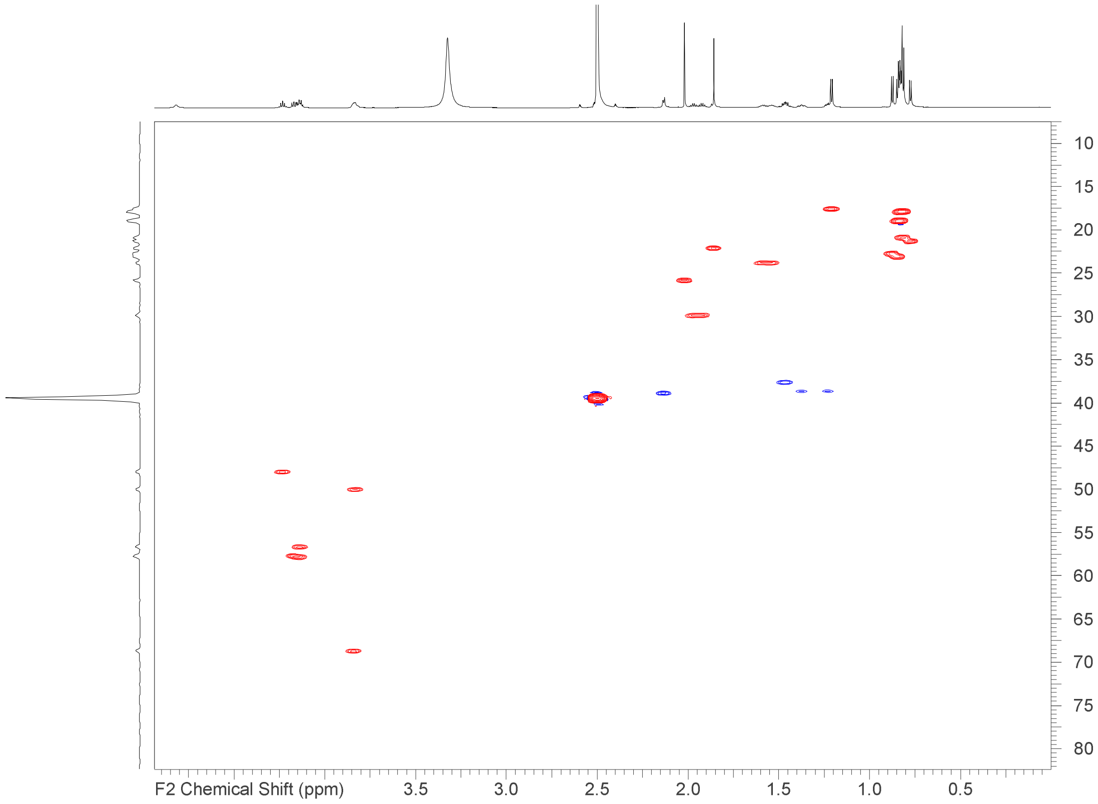

Supplementary Fig. 64. ^{1}H,^{13}C-HSQC spectrum of compound **13** (^{1}H:700 MHz, ^{13}C:175 MHz, DMSO-d_6).

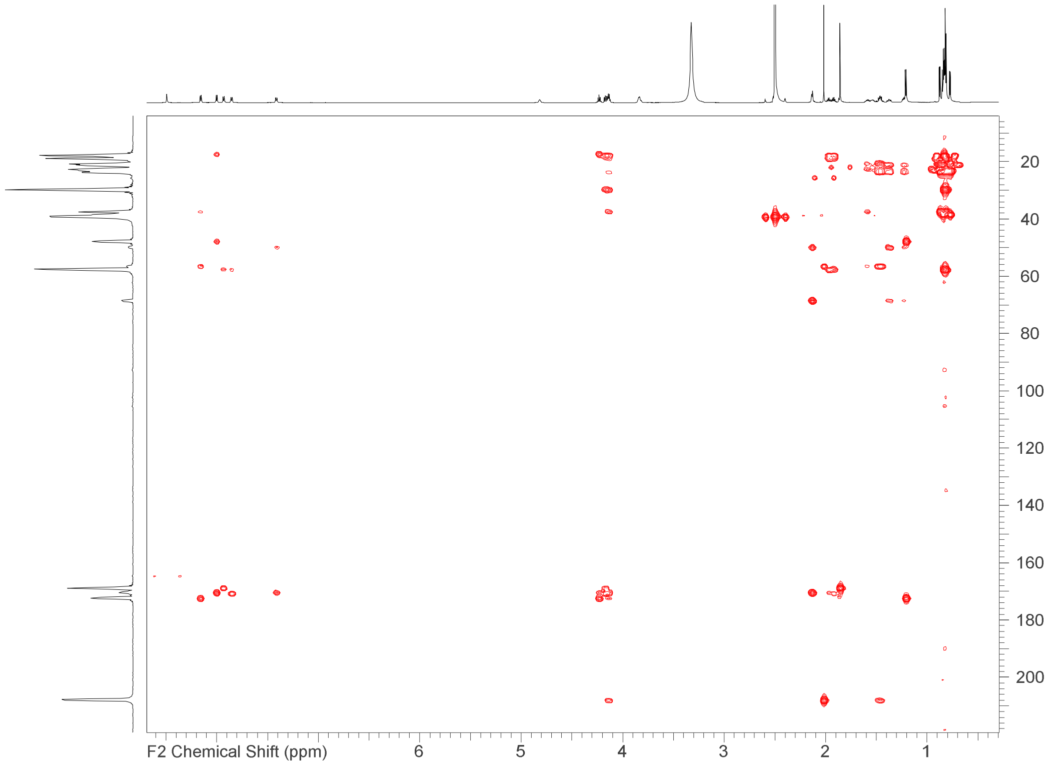

Supplementary Fig. 65. ^{1}H,^{13}C-HMBC spectrum of compound **13** (^{1}H:700 MHz, ^{13}C:175 MHz, DMSO-d_6).

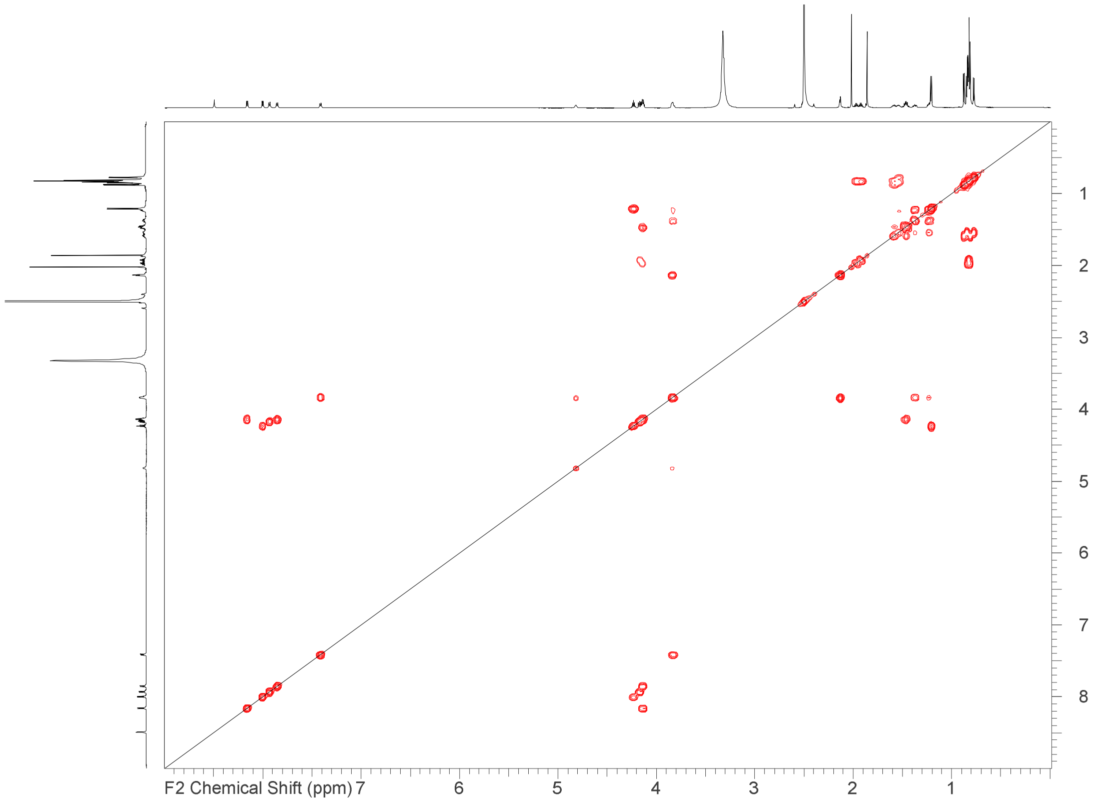

Supplementary Fig. 66. ^{1}H,^{1}H-COSY spectrum of compound **13** (700 MHz, DMSO-d_6).

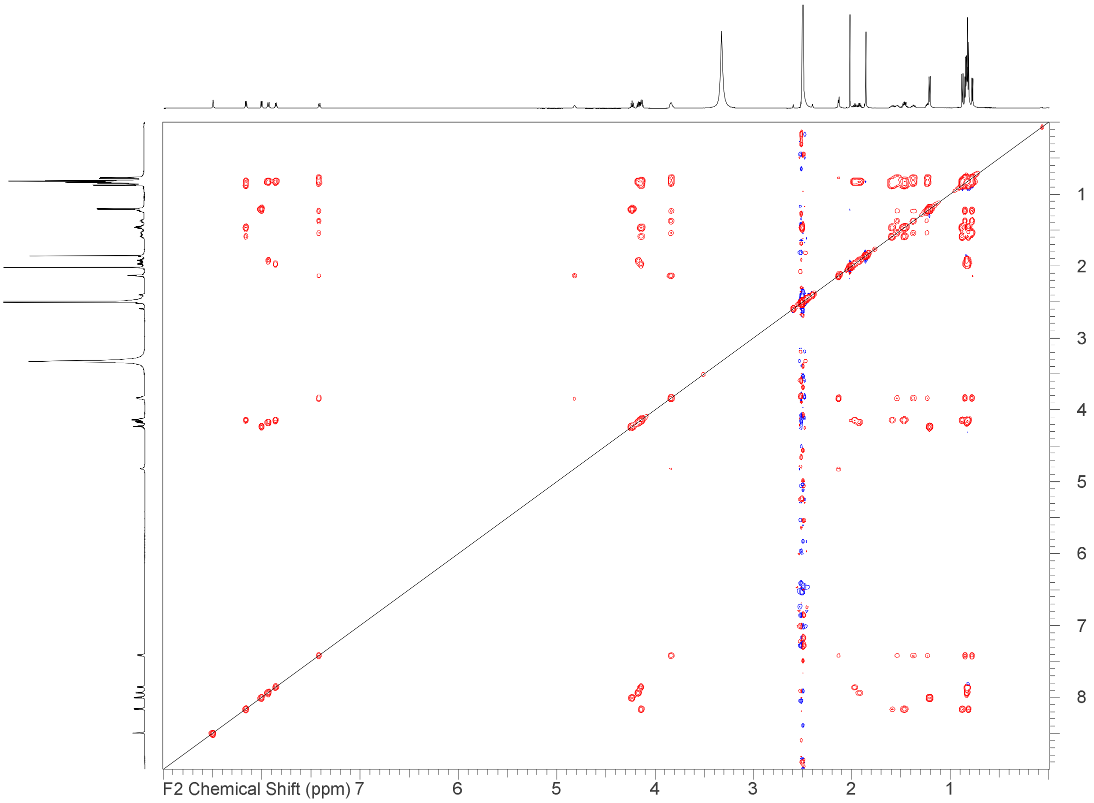

Supplementary Fig. 67. ^{1}H,^{1}H-TOCSY spectrum of compound **13** (700 MHz, DMSO-d_6).

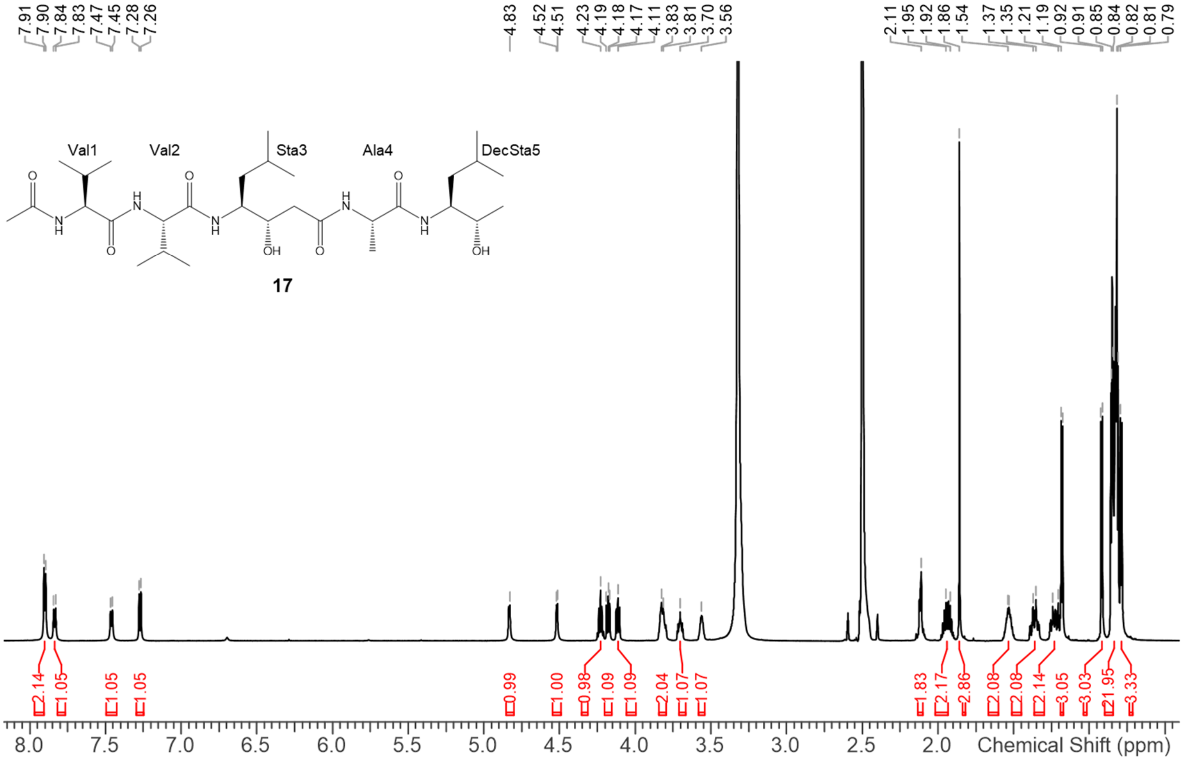

Supplementary Fig. 68. ^{1}H NMR spectrum of compound **17** (700 MHz, DMSO-d_6).

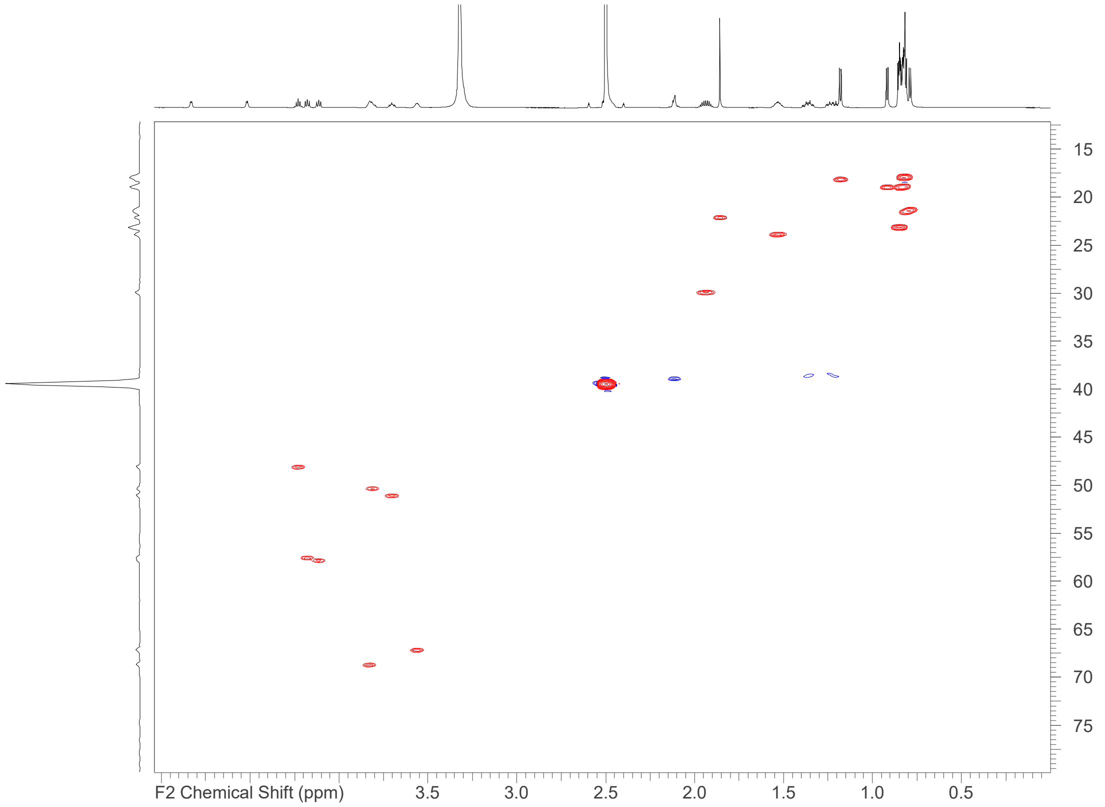

Supplementary Fig. 69. ^{1}H,^{13}C-HSQC spectrum of compound **17** (^{1}H:700 MHz, ^{13}C:175 MHz, DMSO-d_6).

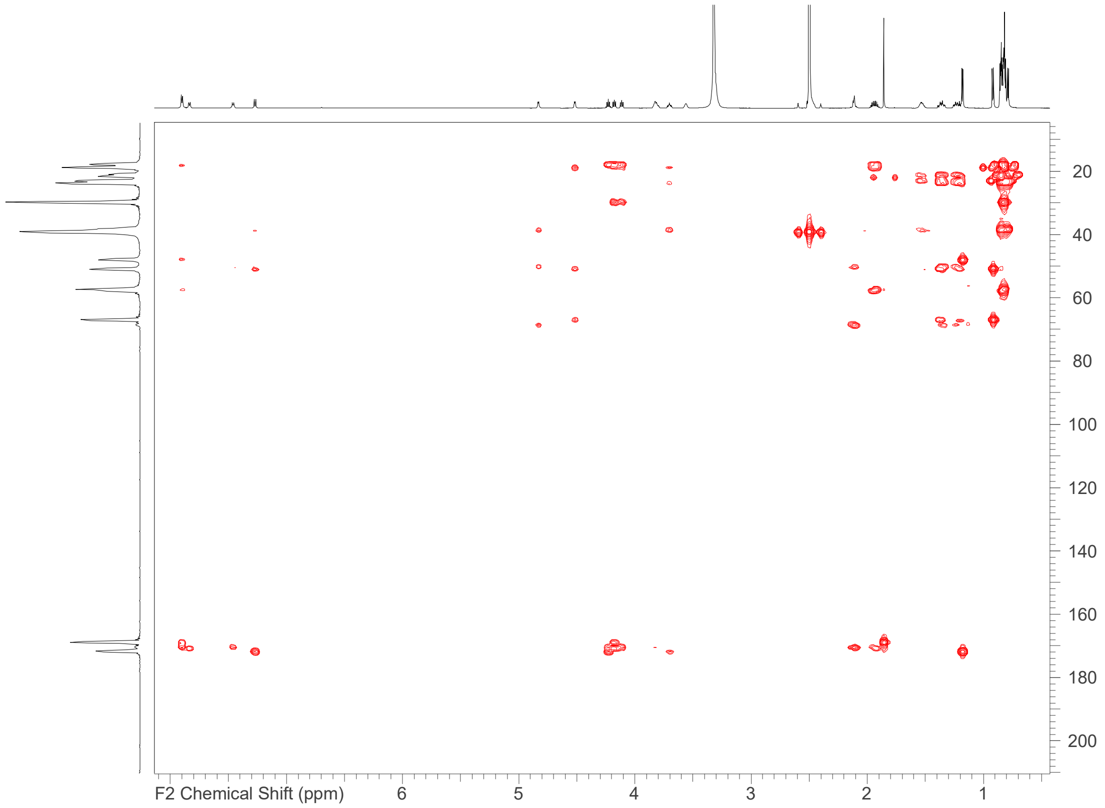

Supplementary Fig. 70. ^{1}H,^{13}C-HMBC spectrum of compound **17** (^{1}H:700 MHz, ^{13}C:175 MHz, DMSO-d_6).

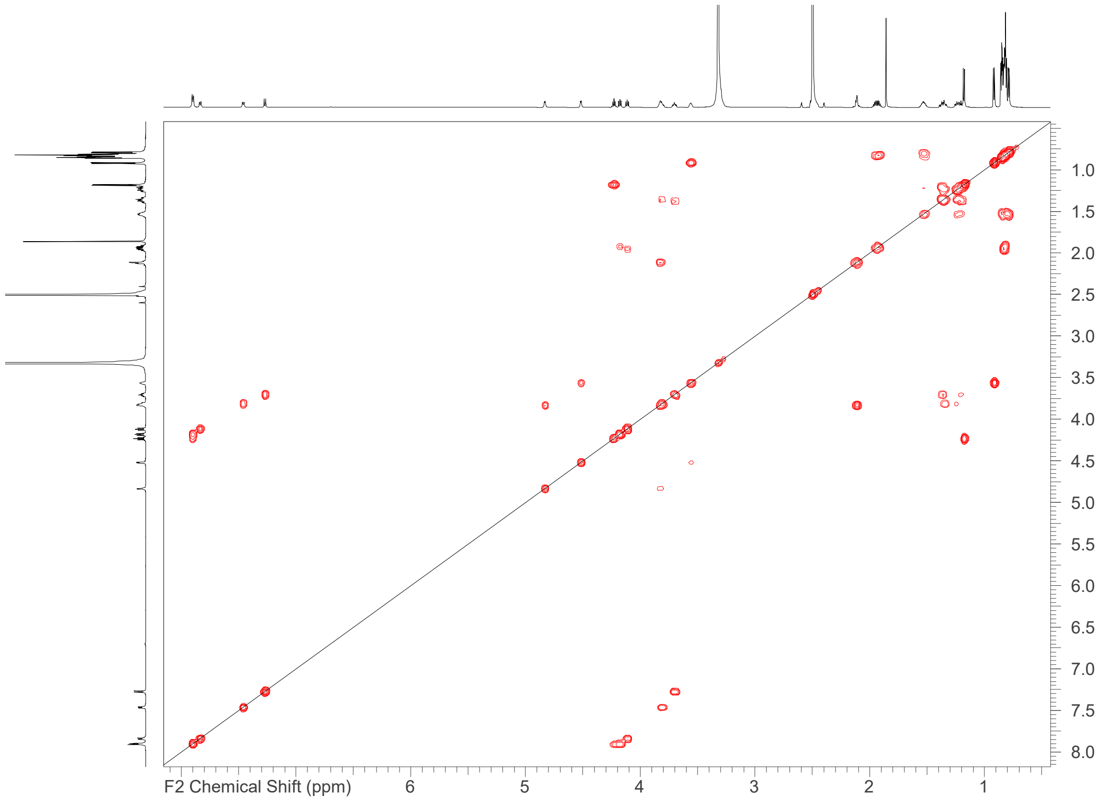

Supplementary Fig. 71. ^{1}H,^{1}H-COSY spectrum of compound **17** (700 MHz, DMSO-d_6).

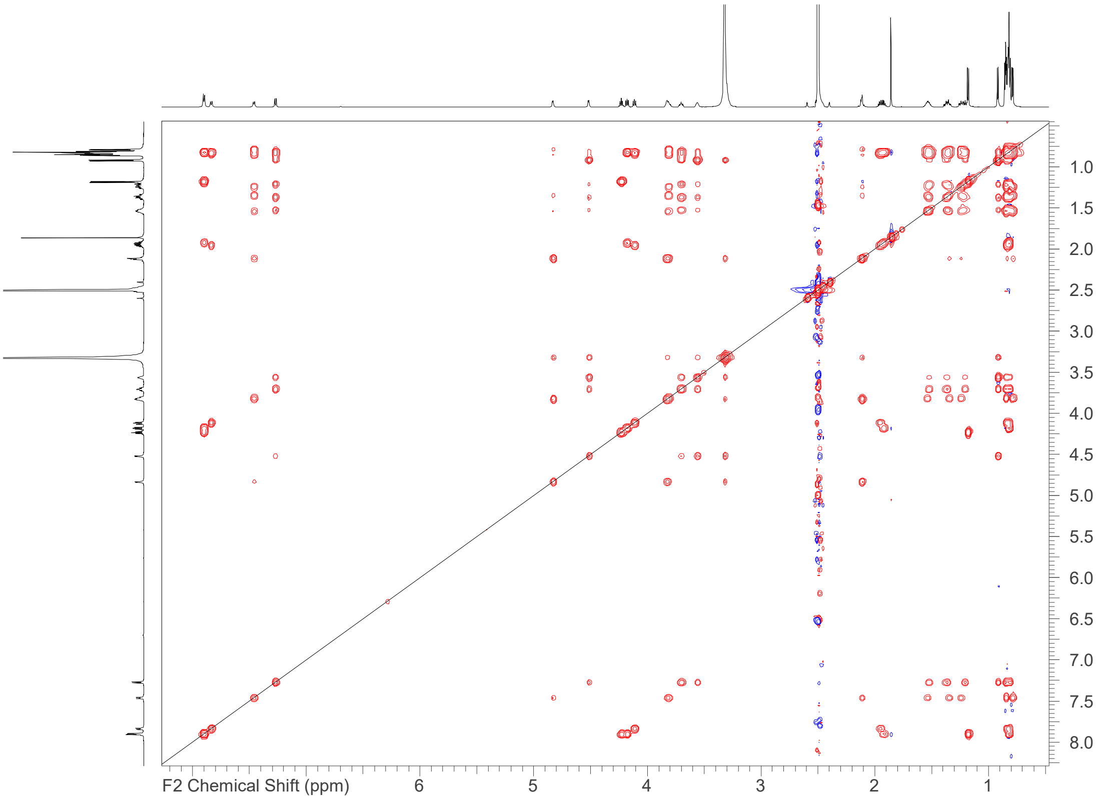

Supplementary Fig. 72. ^{1}H,^{1}H-TOCSY spectrum of compound **17** (700 MHz, DMSO-d_6).

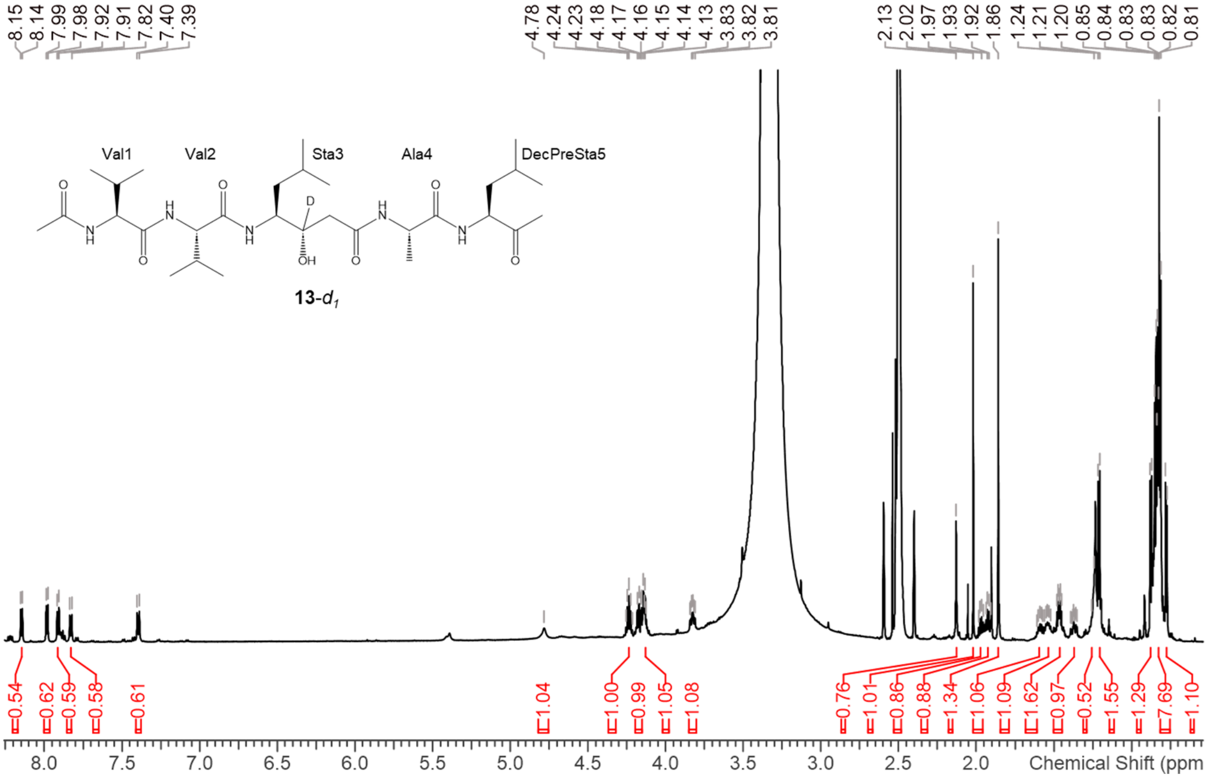

Supplementary Fig. 73. ^{1}H NMR spectrum of compound **13**-d_1 (700 MHz, DMSO-d_6).

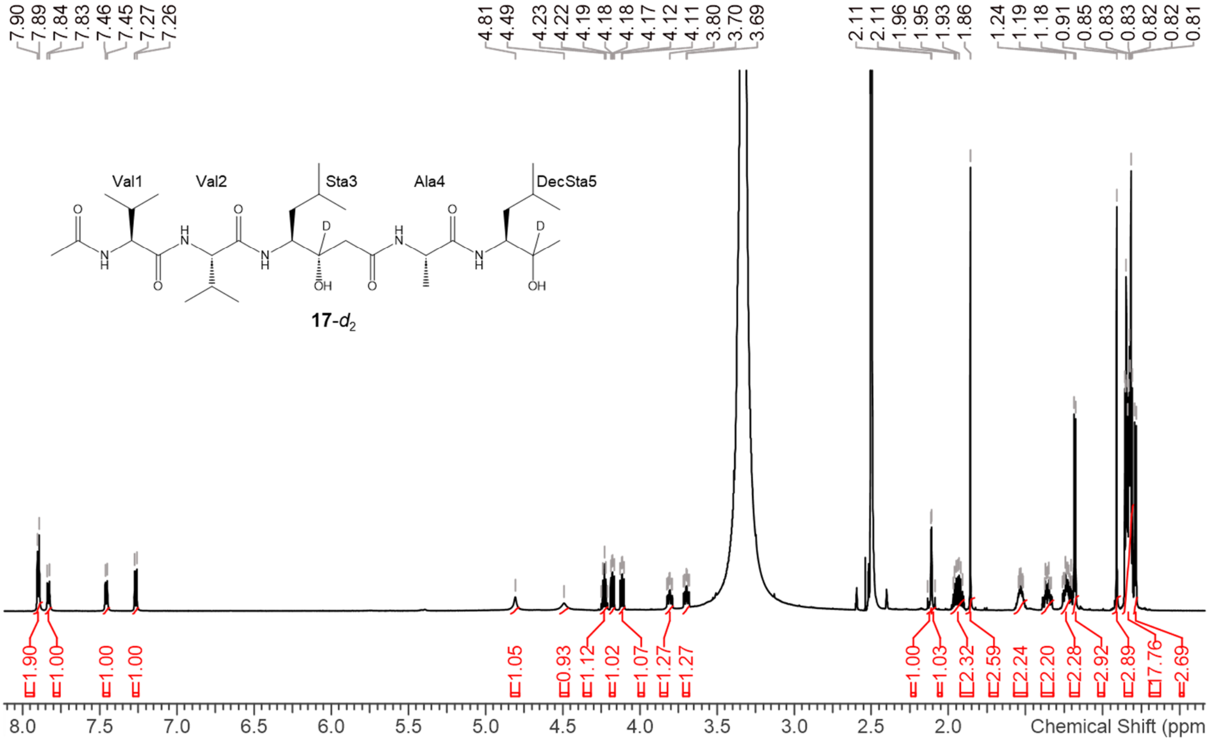

Supplementary Fig. 74. ^{1}H NMR spectrum of compound **17**-d_2 (700 MHz, DMSO-d_6).

Supplementary Tables

Supplementary Table 1. Bacterial strains used in this study**.**

Strains	Characteristic(s)	Source
E. coli		
DH10B	*F – mcrA Δ(mrr-hsdRMS-mcrBC) endA1 recA1 φ80dlacZΔM15 ΔlacX74 araD139 Δ(ara, leu)7697 galU galK rpsL (StrR) nupG λ-*<br><br>Host for general cloning	[5]
GB08-red	Host for Red-ET, *E. coli* GB2005, PBAD-αβγA, *ΔlacZ*; *redα*, *redβ*, *redγ*, and *recA* regulated by the arabinose induced<br><br>Promoter is integrated at the *ybcC* site on chromosome, and *lacZ* is knocked out	[6,7].
BL21(DE3)	*fhuA2 [lon] ompT gal (λ DE3) [dcm] ΔhsdS*<br>*λ DE3 = λ sBamHIo ΔEcoRI-B int::(lacI::PlacUV5::T7 gene1) i21 Δnin5*<br><br>For protein expression	[8]
HB101/pRK2013	Replicon: *ColE1*. Vector for *E.* coli. Carries Tn 903. Contains *RK2* transfer genes. Helper plasmid for mobilisation of non-self-transmissible plasmids.<br><br>For tri-parental conjugation	[9]
ET12567(pUZ8002)	Donor strain for conjugation (methylation deficient)	[10]
Streptomyces		
S. albus Delta14	Heterologous expression host	[11]
S. catenulae DSM40258	Pepstatin producing strain	DSM40258
S. catenulae-△pepD	*pepD* gene knock out	This study
S. albus Del14-*pep*	*pep* gene cluster	This study
S. albus Del14-*pep*-*△pepJ*	*pep* gene cluster, *pepJ* gene knock out	This study
S. albus Del14-*pep-pepJ*-act	*pep* gene cluster, *kasOp* activate *pepJ*	This study
S. albus Del14-*pep*-*△pepI*	*pep* gene cluster, *kasOp* activate *pepJ*, *pepI* knock out	This study

Supplementary Table 2. Plasmids used in this study.

Plasmids	Characteristic(s)	Source
pQS9	*ori(ColE1)*, *rep(pIJ101)*, *oriT(RK2)*, *aac(3)IV*, *E. coli-Streptomyces* shuttle vector for gene editing *Nco*I-*Xba*I sgRNA cloning cassette, ptipA, tsr, *Stu*I, *gusA*, *idgS*	[12]
pR6K-oriT-phiC31	*oriT*, integrase *phiC31* and apramycin resistance genes with a pR6k origin	
p15A-cm-tetR-tetO-hyg-ccdB	*ccdB*, chloramphenicol, hygromycin and tetracycline resistance genes<br>with a p15A origin	
pCold I	*cspA* promoter, *amp*^{r}, *ColE1* origin of replication	Takara
pET-28b	*pBR322* derivative, *T7* terminator, N-6×His tag, N-Thrombin site, N-T7 promoter, C-6×His	
psgQS9-△*pepD*	pQS9 derivative	This study
pQS9-△*pepD*	pQS9 derivative, *pepD in situ* deletion	This study
p15A-*pep*	*p15A ori, harboring pep* BGC	This study
p15A-int-*pep*	*p15A ori,* integrase *phiC31* and apramycin resistance genes, *pep* BGC heterologous expression	This study
p15A-int-*pep*-△*pepJ*	p15A-int-*pep* derivative, gene *pepJ* deletion	This study
p15A-int-*pep*-kasop-*pepJ*	p15A-int-*pep* derivative, promoter *kas*O*p* activate gene *pepJ*	This study
p15A-int-*pep*-△*pepI*	p15A-int-*pep*-kasop-*pepJ* *derivative*, *pepI* deletion	This study
pCold-pepI	pCold derivative, PepI expression plasmid	This study
pET28b-kvpepI	pET-28b derivative, kvPepI expression plasmid	This study
pET28b-kvpepI-H62A	pET-28b derivative, kvPepI mutation	This study
pET28b-kvpepI-Y122F	pET-28b derivative, kvPepI mutation	This study
pET28b-kvpepI-Y122A	pET-28b derivative, kvPepI mutation	This study
pET28b-kvpepI-Q229A	pET-28b derivative, kvPepI mutation	This study
pET28b-kvpepI-Q289A	pET-28b derivative, kvPepI mutation	This study

Supplementary Table 3. Primers used in this study. Restriction sites are underlined.

Primer	Sequence (5' to 3') [a]	Description
sgpepD-f	CATGGATACGCGGTGAGTTGTATTCGTTTTAGAG	sgRNA for cas9
sgpepD-r	CTAGCTCTAAAACGAATACAACTCACCGCGTATC	
pepD-L-f	TCTCGTCGAAGGCACTAGAGGGTGACATCTCGTACATCGAG	Homologous recombination repair template
pepD-L-r	GATGCGGTTCAGGGACCAGT	
pepD-R-f	ACTGGTCCCTGAACCGCATCGGCGACATCTTCCACCACCC	Homologous recombination repair template
pepD-R-r	GGTCGATCCCCGCATATAGGGCTCACCGCCGAAGATGACG	
pepD-check-f	CACACCGGACCTGACCGAAG	*pepD* gene knock out mutant verification
pepD-check-r	CTGTGGCCGAAGAGGACCAC	
p15A-f	CTCGAGAGATCCGAAAACCCCAAGTT	Heterologous expression plasmid backbone amplification
p15A-r	CTCGAGAGATCCTTTCTCCTCTTTAG	
pepJ-ko-f	GAGGGGGCCATTGTGCGGTTGGCGTCGTACGAGGAGATAAATTTCTGCCATTCATCCGCTT	Chloramphenicol resistance gene selection marker
pepJ-ko-r	CAGGTTGTGGCGGGCCACCAGATCGTGGATGACGCTCTTTGTTGATACCGGGAAGCCCTG	
pepJ-act-f	GTGACTGAAAACGATCATCCCGAAGACGCCATATCTCAGAATTTCTGCCATTCATCCGCTT	Promoter *kas*Op and Chloramphenicol resistance gene cassette and verification
pepJ-act-r	GTCATCGGCGAGCACGGCTACGGCAATCGAGCTTCCCAAAACTCCCCCAGTCCTGCACG	
pepJ-check-f	TACATCAGTGTGCATGAATC	*pepJ* gene knock out mutant verification
pepJ-check-r	GTGTAAGTAGACCAGAACAG	
pepI-ko-f	GAATGGACGGACAAGTGCCGTGCCGCGGAAGCGCTCGGCAATTTCTGCCATTCATCCGC	Chloramphenicol resistance gene selection marker
pepI-ko-r	GTCATCGGCGAGCACGGCTACGGCAATCGAGCTTCCCAAAACTCCCCCAGTCCTGCACG	

pepI-check-f	CATCACCTCCCTGACCTCGCC	*pepI* gene knock out mutant verification
pepI-check-r	GTGCGGTTTCTCAGGTTGTG	
pep-check-f	CACGTTGTGCCAGAACTCCTC	BGC *pep* heterologous expression mutant verification
pep-check-r	TCGTGTGGTCCAGGAGCGCG	
pepI-f	ATCATCATATCGAAGGTAGGAACGCTTTCCGTTTCGGTGT	Codon-optimized *pepI* gene amplification
pepI-r	GACTGCAGGTCGACAAGCTTGAATTCTTAACGACCAGCCAGAGCTT	
kvpepI-f	GCCTGGTGCCGCGCGGCAGCACCACCCTGCGTCCGTTCCG	*kvpepI* gene amplification
kvpepI-r	TCGAGTGCGGCCGCAAGCTTTTAACCACGAGCAGAACCCA	
H62A-r	ATGAACGGAGAGTGAACACCCAG**CGC**GTCCGGAACAGCGATAACGTCGT	H62A mutation
H62A-f	ACGACGTTATCGCTGTTCCGGAC**GCG**CTGGGTGTTCACTCTCCGTTCAT	
Y122F-r	GCGGTTTCGAATTCAGCACGAAC**AAA**ACCGGTACCCAGACCAGCTTCAA	Y122F mutation
Y122F-f	TTGAAGCTGGTCTGGGTACCGGT**TTT**GTTCGTGCTGAATTCGAAACCGC	
Y122A-r	GCGGTTTCGAATTCAGCACGAAC**CGC**ACCGGTACCCAGACCAGCTTCAA	Y122A mutation
Y122A-f	TTGAAGCTGGTCTGGGTACCGGT**GCG**GTTCGTGCTGAATTCGAAACCGC	
Q229A-r	TCGTCGGTAGCGATAACAGA**CGC**AACCAGGGTGTTACGTTCAA	Q229A mutation
Q229A-f	TTGAACGTAACACCCTGGTT**GCG**TCTGTTATCGCTACCGACGA	
pET-28b-f	TGATGTCTCTGACCAGACAC	site mutation gene expression backbone
pET-28b-r	GAAATCACCATGAGTGACGA	
Q289A-r	AACGCCGCGAGGTAACGTTC**CGC**AACGCAAACGTAAGAGAAGC	Q289A mutation
Q289A-f	GCTTCTCTTACGTTTGCGTT**GCG**GAACGTTACCTCGCGGCGTT	

[a]Gene-specific guiding sequences for CRISPR-Cas9 gene editing are double-underlined. The homologous arms for Gibson assembly or Red-ET are underlined, the mutation sites in kvPepI mutants are marked in **bold**.

Supplementary Table 4. Gene annotation of *pep* BGC.

Gene	Size (aa)	Protein homolog	Proposed function	Putative catalytic domains
pepA	95	WP_051739820.1	phosphopantetheine binding protein	PCP
pepB	1100	WP_157848057.1	NRPS	C, A, PCP
pepC	898	WP_051739811.1	Type I PKS	KS, AT, PCP
pepD	1093	WP_051739812.1	NRPS	C, A, PCP
pepE	278	WP_078654176.1	thioesterase	TE
pepF	63	WP_030286418.1	mbtH	
pepG	530	WP_051739813.1	NRPS	A
pepH	1036	WP_078654178.1	NRPS	C, PCP
pepI	295	WP_030286424.1	methylenetetrahydromethanopterin reductase	
pepJ	212	WP_051739822.1	helix-turn-helix transcriptional regulator	

Supplementary Table 5. Substrate specificity analysis of the three A domains from *pep* NRPSs.

**A domain**	**ORF**	**stachelhaus_code**[a]	**Predicted amino acid**[b]	**Biosynthesis assumed amino acid**[c]
A1	*pepB*	DAWFLGNVVK	Leu	Leu
A2	*pepD*	DVWHLSALEK	Ala	Ala
A3	*pepG*	DFWNIGMVFK	Thr	Val

[a] Residues defined according to Stachelhaus et al.[13]

[b] Predicted by antiSMASH bacteria version gene cluster analysis according to Stachelhaus prediction.[14]

[c] Amino acids incorporated in the pepstatin structures.

Supplementary Table 6. Classification of *pep*-like BGCs.

**Strain**	**BGC Type (according to the feature of PKS)**
Streptomyces catenulae DSM 40258	*cis-AT* PKS
Streptomyces sp. SF28	*cis-AT* PKS
Streptomyces sp. BHT-5-2	*cis-AT* PKS
Streptomyces sp. PTM05	*cis-AT* PKS
Streptomyces varsoviensis NRRL ISP-5346	*cis-AT* PKS
Kitasatospora kifunensis DSM 41654	*trans-AT* PKS
Kitasatospora viridis DSM 44826	*trans-AT* PKS
Streptomyces cattleya DSM 46488	*without* PKS
Streptomyces sp. ICBB 8177	*without* PKS

Supplementary Table 7. X-ray data collection and refinement statistics.

	kvPepI_apo	kvPepI_F_{420}	kvPepI^{H62A}	kvPepI^{H62A} _F_{420}	kvPepI^{Y122A} _F_{420}
**PDB ID**	9G64	9GM0	9GKH	9GND	9GNC
**Data collection**					
Space group	$P\ 2_1 2_1 2_1$	$P\ 2_1 2_1 2_1$	$P\ 2_1 2_1 2_1$	$P\ 2_1 2_1 2_1$	$P\ 2_1 2_1 2_1$
Cell dimension					
a, b, c (Å)	56.14, 94.79, 111.05	56.35, 94.27, 111.02	56.21, 94.28, 110.82	56.52, 94.27, 110.64	56.31, 94.59, 110.76
α, β, γ (°)	90.0, 90.0, 90.0	90.0, 90.0, 90.0	90.0, 90.0, 90.0	90.0, 90.0, 90.0	90.00, 90.00, 90.00
Wavelength (Å)	1.033	1.033	1.033	1.033	1.033
Resolution	48.31 – 1.65 (1.68 – 1.65)*	48.37 – 1.65 (1.68 – 1.65)*	48.28 – 1.70 (1.73 – 1.70)	48.48 – 1.80 (1.84 – 1.80)	48.39 – 1.65 (1.68 – 1.65)
R_{sym} or R_{merge}	0.123 (1.756)	0.086 (1.431)	0.084 (1.450)	0.243 (2.155)	0.154 (1.797)
R_{pim}	0.024 (0.346)	0.035 (0.587)	0.020 (0.383)	0.067 (0.585)	0.037 (0.426)
CC (1/2)	0.999 (0.830)	0.999 (0.703)	1.000 (0.890)	0.998 (0.820)	0.999 (0.847)
I / σI	19.4 (2.5)	17.1 (1.9)	28.5 (2.7)	12.3 (2.3)	21.2 (3.5)
Completeness (%)	99.5 (99.7)	100.0 (100.0)	100.0 (100.0)	99.9 (100.0)	99.5 (99.3)
Redundancy	26.8 (27.0)	13.4 (13.5)	34.3 (28.9)	26.3 (27.6)	34.3 (34.9)
Refinement					
Resolution (Å)	48.31 – 1.65	48.37 –1.65	48.28 – 1.70	48.48 – 1.80	48.39 – 1.65
No. reflection	71666 (3528)	71956 (3513)	65590 (3416)	55564 (3272)	71529 (3484)
R_{work} / R_{free}	0.175 / 0.191	0.169 / 0.188	0.181 / 0.199	0.177 / 0.209	0.169 / 0.192
No. atoms	4583	4721	4472	4602	4771
Protein	4095	4118	4079	4104	4104

Ligands	-	53	-	53	53
Solvent	488	550	393	445	611
Protein residues	539	545	538	544	545
B-factors	26.39	26.79	28.88	30.35	25.27
Protein	25.12	25.30	28.06	29.11	23.59
Ligands	-	35.24	-	66.85	36.75
Water	37.08	37.12	37.38	37.46	35.54
R. m. s deviations					
Bond length (Å)	0.008	0.009	0.011	0.010	0.009
Bond angels (°)	0.93	1.08	1.09	1.05	1.07
MolProbity score	0.96	0.96	1.03	0.97	0.84

*Values in parentheses are for highest-resolution shell

NMR Tables

Supplementary Table 8. NMR spectroscopic data of compounds **1-4** (methanol-d_4).

No.	**1**[a]		**2**[a]		**3**[b]		**4**[a]	
	δ_H (J in Hz)	δ_C	δ_H (J in Hz)	δ_C	δ_H (J in Hz)	δ_C	δ_H (J in Hz)	δ_C
Val1								
1		174.1		174.1		174.1		174.0
2	4.16 (1H, d, 7.7)	60.7	4.17 (1H, d, 7.8)	60.6	4.16 (1H, d, 8.0)	60.5	4.18 (1H, d, 8.3)	60.4
3	2.07 (1H, m)	31.6	2.07 (1H, m)	31.6	2.06 (1H, m)	31.6	2.06 (1H, m)	31.6
4	0.98 (3H, d, 6.4)	18.9	0.98 (3H, d, 6.6)	19.0	0.97 (3H, m [c])	18.9	0.97 (3H, m [c])	18.9
5	0.96 (3H, d, 6.8)	19.9	0.96 (3H, d, 6.7)	19.9	0.98 (3H, m [c])	19.9	0.98 (3H, m [c])	19.9
Val2								
1		173.8		173.8		173.8		173.8
2	4.12 (1H, d, 7.9)	61.0	4.12 (1H, d, 7.9)	60.9	4.13 (1H, d, 7.7)	60.8	4.13 (1H, d, 7.7)	60.8
3	2.07 (1H, m)	31.6	2.07 (1H, m)	31.6	2.09 (1H, m)	31.6	2.08 (1H, m)	31.6
4	0.96 (3H, d, 6.7)	19.0	0.96 (3H, d, 6.8)	19.0	0.97 (3H, m [c])	19.0	0.97 (3H, m [c])	19.0
5	0.99 (3H, d, 6.2)	20.0	0.99 (3H, d, 6.3)	20.0	0.98 (3H, m [c])	20.0	0.98 (3H, m [c])	20.0
Sta3								
1		173.8		173.8		173.8		173.7
2	2.33 (1H, m [c])	41.4	2.34 (2H, m [c])	41.4	2.35 (2H, m [c])	41.4	2.34 (2H, m [c])	41.4
	2.42 (1H, dd, 15.9, 4.3)							
3	4.01 (1H, m)	71.1	4.00 (1H, m)	71.1	4.01 (1H, m)	71.1	4.00 (1H, m)	71.3
4	3.91 (1H, m)	52.8	3.89 (1H, m)	52.9	3.89 (1H, m)	52.9	3.89 (1H, m)	52.9
5	1.34 (1H, m)	41.1	1.34 (1H, m)	41.1	1.36 (1H, m)	41.1	1.34 (1H, m)	41.1
	1.59 (1H, m)		1.57 (1H, m)		1.58 (1H, m)		1.57 (1H, m)	
6	1.63 (1H, m)	25.9	1.63 (1H, m)	25.9	1.65 (1H, m)	25.9	1.64 (1H, m)	25.8
7	0.91 (3H, d, 6.7)	23.8	0.91 (3H, d, 6.9)	23.8	0.91 (3H, d, 6.6)	23.8	0.91 (3H, m [c])	23.8
8	0.88 (3H, d, 6.4)	22.4	0.88 (3H, d, 6.5)	22.4	0.88 (3H, d, 6.5)	22.4	0.88 (3H, m [c])	22.4
Ala4								
1		175.4		175.3		175.3		175.2

2	4.33 (1H, q, 7.2)	50.9	4.33 (1H, q, 7.2)	50.8	4.33 (1H, q, 7.2)	50.9	4.33 (1H, q, 7.2)	50.8
3	1.38 (3H, d, 7.1)	18.3	1.38 (3H, d, 7.2)	18.4	1.38 (3H, d, 7.2)	18.4	1.38 (3H, d, 7.2)	18.4
Sta5								
1		175.9		177.2		177.0		177.5
2	2.31 (1H, dd, 5.6, 14.7)	40.1	2.36 (2H, m [c])	40.7	2.38 (2H, m [c])	40.5	2.33 (2H, m [c])	40.7
	2.35 (1H, m [c])							
3	4.01 (1H, m)	71.1	4.00 (1H, m)	71.3	4.01 (1H, m)	71.2	4.00 (1H, m)	71.1
4	4.00 (1H, m)	52.4	3.98 (1H, m)	52.5	3.98 (1H, m)	52.5	3.97 (1H, m)	52.5
5	1.34 (1H, m)	41.1	1.31 (1H, m)	41.4	1.35 (1H, m)	41.4	1.35 (1H, m)	41.4
	1.59 (1H, m)		1.60 (1H, m)		1.58 (1H, m)		1.58 (1H, m)	
6	1.63 (1H, m)	25.8	1.63 (1H, m)	25.8	1.62 (1H, m)	25.8	1.62 (1H, m)	25.9
7	0.93 (3H, d, 6.6)	23.8	0.93 (3H, d, 6.8)	23.8	0.93 (3H, d, 6.7)	23.7	0.93 (3H, m [c])	23.8
8	0.90 (3H, d, 6.6)	22.4	0.90 (3H, d, 6.7)	22.4	0.90 (3H, d, 6.5)	22.4	0.90 (3H, m [c])	22.4
FA								
1		173.5		169.8		180.2		179.6
2	2.00 (3H, s)	22.4	2.28 (2H, q, 7.7)	29.9	2.56 (1H, m)	36.0	2.36 (1H, m)	43.4
3			1.13 (3H, t, 7.7)	10.5	1.10 (3H, d, 6.9)	19.8	1.38 (1H, m)	28.3
							1.61 (1H, m)	
4					1.14 (3H, d, 6.9)	20.1	0.87 (3H, m [c])	12.4
5							1.11 (3H, d, 6.9)	18.2

[a]Measured at 500 MHz for ^{1}H NMR
[b]Measured at 700 MHz for ^{1}H NMR
[c]*J*-value not determined due to overlapped signals

Supplementary Table 9. NMR spectroscopic data of compounds **9-12** (^{1}H: 500 MHz, ^{13}C: 125 MHz, DMSO-d_6).

No.	**9**		**10**		**11**		**12**	
	δ_H (J in Hz)	δ_C	δ_H (J in Hz)	δ_C	δ_H (J in Hz)	δ_C	δ_H (J in Hz)	δ_C
Val1								
1		171.2		171.2		171.3		171.3
2	4.21 (1H, dd, 8.7, 6.9)	57.6	4.20 (1H, dd, 8.7, 7.1)	57.6	4.18 (1H, dd, 7.1, 8.7)	57.5	4.20 (1H, dd, 7.6, 8.7)	57.5
3	1.91 (1H, m)	30.4	1.92 (1H, m)	30.4	1.94 (1H, m)	30.2	1.92 (1H, m)	30.3
4	0.81 (3H, m [c])	18.1	0.81 (3H, m [c])	18.2	0.82 (3H, m [c])	18.2	0.83 (3H, m [c])	18.3
5	0.82 (3H, m [c])	19.2	0.82 (3H, m [c])	19.2	0.83 (3H, m [c])	19.2	0.84 (3H, m [c])	19.2
NH	7.90 (1H, d, 8.9)		7.82 (1H, d, 8.9)		7.80 (1H, d, 8.8)		7.82 (1H, d, 8.8)	
Val2								
1		171.2		171.1		171.1		171.1
2	4.12 (1H, m [c])	57.9	4.12 (1H, m [c])	57.8	4.14 (1H, m)	57.7	4.15 (1H, m)	57.8
3	1.93 (1H, m)	30.3	1.95 (1H, m)	30.3	1.95 (1H, m)	30.4	1.95 (1H, m)	30.4
4	0.84 (3H, m [c])	18.2	0.84 (3H, m [c])	18.4	0.82 (3H, m [c])	18.3	0.83 (3H, m [c])	18.3
5	0.85 (3H, m [c])	19.3	0.85 (3H, m [c])	19.3	0.84 (3H, m [c])	19.2	0.84 (3H, m [c])	19.2
NH	7.88 (1H, d, 9.0)		7.84 (1H, d, 9.0)		7.78 (1H, d, 9.0)		7.78 (1H, d, 8.9)	
PreSta3								
1		165.8		165.8		165.8		165.8
2	3.45 (1H, d, 16.0)	46.9	3.45 (1H, d, 16.0)	46.9	3.45 (1H, d, 15.9)	46.9	3.45 (1H, d, 15.9)	46.9
	3.38 (1H, d, 16.0)		3.38 (1H, d, 16.0)		3.38 (1H, d, 15.9)		3.38 (1H, d, 15.9)	
3		204.9		204.9		204.9		204.9
4	4.30 (1H, m)	56.6	4.30 (1H, m)	56.6	4.30 (1H, m)	56.6	4.30 (1H, m)	56.6
5	1.45 (2H, m)	37.8	1.45 (2H, m)	37.8	1.45 (2H, m)	37.8	1.45 (2H, m)	37.8
6	1.59 (1H, m)	24.1	1.58 (1H, m)	24.1	1.59 (1H, m)	24.1	1.59 (1H, m)	24.1
7	0.86 (3H, m [c])	23.2	0.86 (3H, m [c])	23.2	0.86 (3H, m [c])	23.2	0.86 (3H, m [c])	23.2

8	0.80 (3H, m [c])	20.9	0.80 (3H, m [c])	20.9	0.80 (3H, m [c])	20.9	0.80 (3H, m [c])	20.9
NH	8.31 (1H, d, 7.7)		8.31 (1H, d, 7.7)		8.30 (1H, d, 7.8)		8.30 (1H, d, 7.8)	
Ala4								
1		172.4		172.4		172.4		172.4
2	4.28 (1H, m)	48.3	4.28 (1H, m)	48.3	4.28 (1H, m)	48.3	4.28 (1H, m)	48.3
3	1.22 (3H, d, 7.1)	18.1	1.22 (3H, d, 7.1)	18.1	1.22 (3H, d, 7.1)	18.1	1.22 (3H, d, 7.1)	18.0
NH	8.34 (1H, d, 7.3)		8.34 (1H, d, 7.3)		8.34 (1H, d, 7.4)		8.35 (1H, d, 7.3)	
DecPreSta5								
1	2.02 (3H, s)	26.1	2.02 (3H, s)	26.1	2.02 (3H, s)	26.1	2.02 (3H, s)	26.1
2		208.1		208.1		208.1		208.0
3	4.14 (1H, m)	57.1	4.15 (1H, m)	57.0	4.14 (1H, m)	57.0	4.15 (1H, m)	57.0
4	1.45 (2H, m)	38.1	1.45 (2H, m)	38.1	1.45 (2H, m)	38.1	1.45 (2H, m)	38.1
5	1.60 (1H, m)	24.2	1.61 (1H, m)	24.2	1.60 (1H, m)	24.2	1.60 (1H, m)	24.2
6	0.88 (3H, m [c])	23.1	0.88 (3H, m [c])	23.1	0.88 (3H, m [c])	23.1	0.88 (3H, m [c])	23.1
7	0.83 (3H, m [c])	21.2	0.83 (3H, m [c])	21.2	0.83 (3H, m [c])	21.2	0.83 (3H, m [c])	21.2
NH	8.20 (1H, d, 7.6)		8.20 (1H, d, 7.6)		8.20 (1H, d, 7.6)		8.21 (1H, d, 7.6)	
FA								
1		169.3		173.1		176.3		175.6
2	1.85 (3H, s)	22.5	2.15 (2H, q, 7.5)	28.3	2.52 (1H, m)	33.6	2.32 (1H, m)	40.9
3			0.97 (3H, t, 7.6)	10.1	0.99 (3H, d, 6.8)	19.9	1.46 (1H, m)<br>1.25 (1H, m)	26.9
4					0.95 (3H, d, 6.8)	19.4	0.76 (3H, t, 7.4)	11.7
5							0.96 (3H, d, 6.8)	18.0

[c] *J*-value not determined due to overlapped signals

Supplementary Table 10. NMR spectroscopic data of compounds **13, 13-*d*$_1$, 17** and **17-*d*$_2$** (^{1}H: 700 MHz, ^{13}C: 175 MHz, DMSO-*d*$_6$).

No.	**13**		**13-*d***	**17**		**17-*d*$_2$**
	δ_H (J in Hz)	δ_C	δ_H (J in Hz)	δ_H (J in Hz)	δ_C	δ_H (J in Hz)
Val1						
1		170.9			170.8	
2	4.17 (1H, dd, 7.2, 8.8)	57.7	4.17 (1H, m)	4.18 (1H, dd, 7.1, 8.6)	57.6	4.18 (1H, dd, 7.0, 8.7)
3	1.92 (1H, m)	29.9	1.92 (1H, m)	1.92 (1H, m)	30.0	1.92 (1H, m)
4	0.83 (3H, m [c])	18.0	0.83 (3H, m [c])	0.82 (3H, m [c])	17.9	0.82 (3H, m [c])
5	0.83 (3H, m [c])	18.9	0.83 (3H, m [c])	0.82 (3H, m [c])	18.9	0.82 (3H, m [c])
NH	7.93 (1H, d, 8.7)		7.91 (1H, d, 8.7)	7.90 (1H, br d, 7.8)		7.90 (1H, d, 7.6)
Val2						
1		170.5			170.3	
2	4.14 (1H, m)	57.8	4.14 (1H, m)	4.11 (1H, dd, 7.6, 8.9)	57.8	4.11 (1H, dd, 7.5, 9.0)
3	1.97 (1H, m)	29.9	1.97 (1H, m)	1.95 (1H, m)	30.0	1.95 (1H, m)
4	0.82 (3H, m [c])	17.9	0.82 (3H, m [c])	0.84 (3H, m [c])	18.0	0.84 (3H, m [c])
5	0.84 (3H, m [c])	19.1	0.84 (3H, m [c])	0.84 (3H, m [c])	19.0	0.84 (3H, m [c])
NH	7.85 (1H, d, 9.0)		7.83 (1H, d, 9.1)	7.84 (1H, d, 8.9)		7.83 (1H, d, 9.0)
Sta3						
1		170.5			170.4	
2	2.14 (2H, m)	39.0	2.13 (2H, br s)	2.11 (2H, m)	39.0	2.10 (1H, d, 14.5)
						2.12 (1H, d, 14.5)
3	3.84 (1H, m)	68.7		3.83 (1H, m)	68.7	
4	3.84 (1H, m)	50.1	3.83 (1H, dt, 4.3, 9.8)	3.81 (1H, m)	50.4	3.81 (1H, m)
5	1.23 (1H, m)	38.8	1.24 (1H, m)	1.24 (1H, m)	38.5	1.24 (1H, m)
	1.38 (1H, m)		1.37 (1H, m)	1.35 (1H, m)		1.35 (1H, m)
6	1.54 (1H, m)	23.9	1.54 (1H, m)	1.54 (1H, m)	24.0	1.54 (1H, m)
7	0.78 (3H, d, 6.5)	21.4	0.78 (3H, d, 6.5)	0.79 (3H, d, 6.5)	21.4	0.79 (3H, d, 6.4)
8	0.85 (3H, m [c])	23.2	0.85 (3H, m [c])	0.85 (3H, m [c])	23.2	0.85 (3H, m [c])
NH	7.41 (1H, d, 9.0)		7.39 (1H, d, 9.2)	7.46 (1H, d, 8.8)		7.46 (1H, d, 9.0)
3-OH	4.82 (1H, br s)		4.78 (1H, br s)	4.83 (1H, d, 4.8)		4.81 (1H, br s)
Ala4						
1		172.4			171.8	
2	4.23 (1H, dq, 7.1)	48.1	4.23 (1H, dq, 7.1)	4.23 (1H, dq, 7.1)	48.1	4.23 (1H, dq, 7.0)
3	1.21 (3H, d, 7.1)	17.7	1.21 (3H, d, 7.1)	1.18 (3H, d, 7.1)	18.2	1.18 (3H, d, 7.0)
NH	8.00 (1H, d, 7.1)		7.98 (1H, d, 7.2)	7.90 (1H, br d, 7.8)		7.90 (1H, d, 7.6)
Dec(Pre)Sta5						

1	2.02 (3H, s)	26.0	2.02 (3H, s)	0.92 (3H, d, 6.3)	19.1	0.91 (3H, s)
2		207.9		3.56 (1H, m)	67.2	
3	4.14 (1H, m)	56.7	4.14 (1H, m)	3.70 (1H, m)	51.1	3.70 (1H, m)
4	1.47 (2H, m)	37.7	1.46 (2H, m)	1.21 (1H, m)	38.7	1.21 (1H, m)
				1.37 (1H, m)		1.37 (1H, m)
5	1.59 (1H, m)	23.9	1.59 (1H, m)	1.53 (1H, m)	24.0	1.53 (1H, m)
6	0.82 (3H, m [c])	20.9	0.82 (3H, m [c])	0.81 (3H, m [c])	21.6	0.81 (3H, m [c])
7	0.88 (3H, d, 6.7)	22.8	0.88 (3H, d, 6.7)	0.85 (3H, m [c])	23.2	0.85 (3H, m [c])
NH	8.16 (1H, d, 7.8)		8.15 (1H, d, 7.8)	7.27 (1H, d, 9.2)		7.27 (1H, d, 9.3)
2-OH				4.52 (1H, d, 4.7)		4.49 (1H, br s)
FA						
1		169.0			169.0	
2	1.86 (3H, s)	22.2	1.86 (3H, s)	1.86 (3H, s)	22.3	1.86 (3H, s)

[c] *J*-value not determined due to overlapped signals

Supplementary References

1. Świecimska, M., Golińska, P. & Goodfellow, M. Genome-based classification of *Streptomyces pinistramenti* sp. nov., a novel actinomycete isolated from a pine forest soil in Poland with a focus on its biotechnological and ecological properties. *Antonie van Leeuwenhoek* **115,** 783–800; 10.1007/s10482-022-01734-8. (2022).

2. Liu, Z. *et al. Kitasatospora viridis* sp. nov., a novel actinomycete from soil. *International Journal of Systematic and Evolutionary Microbiology* **55,** 707–711; 10.1099/ijs.0.63329-0 (2005).

3. Ayed, A. *et al.* Draft genome sequence of *Streptomyces tunisialbus* DSM 105760T. *Archives of Microbiology* **202,** 2013–2017; 10.1007/s00203-020-01913-z (2020).

4. Marfey, P. Determination of D-amino acids. II. Use of a bifunctional reagent, 1,5-difluoro-2,4-dinitrobenzene. *Carlsberg Res. Commun.* **49,** 591–596; 10.1007/BF02908688 (1984).

5. Durfee, T. *et al.* The complete genome sequence of *Escherichia coli* DH10B: insights into the biology of a laboratory workhorse. *Journal of Bacteriology* **190,** 2597–2606; 10.1128/JB.01695-07 (2008).

6. Zhang, Y., Muyrers, J. P., Testa, G. & Stewart, A. F. DNA cloning by homologous recombination in *Escherichia coli*. *Nature Biotechnology* **18,** 1314–1317; 10.1038/82449 (2000).

7. Zhang, Y., Buchholz, F., Muyrers, J. P. & Stewart, A. F. A new logic for DNA engineering using recombination in *Escherichia coli*. *Nature Genetics* **20,** 123–128; 10.1038/2417 (1998).

8. Studier, F. W. & Moffatt, B. A. Use of bacteriophage T7 RNA polymerase to direct selective high-level expression of cloned genes. *Journal of Molecular Biology* **189,** 113–130; 10.1016/0022-2836(86)90385-2 (1986).

9. Klug, G. & Drews, G. Construction of a gene bank of *Rhodopseudomonas capsulata* using a broad host range DNA cloning system. *Archives of Microbiology* **139,** 319–325; 10.1007/BF00408373 (1984).

10. Kieser, T. *Practical streptomyces genetics* (John Innes Foundation, Norwich, 2000).

11. Rodríguez Estévez, M., Myronovskyi, M., Gummerlich, N., Nadmid, S. & Luzhetskyy, A. Heterologous expression of the nybomycin gene cluster from the marine strain *Streptomyces albus* subsp. *chlorinus* NRRL B-24108. *Marine Drugs* **16**; 10.3390/md16110435 (2018).

12. Wang, Q. *et al.* Dual-function chromogenic screening-based CRISPR/Cas9 genome editing system for actinomycetes. *Applied Microbiology and Biotechnology* **104,** 225–239; 10.1007/s00253-019-10223-4 (2020).

13. Stachelhaus, T., Mootz, H. D. & Marahiel, M. A. The specificity-conferring code of adenylation domains in nonribosomal peptide synthetases. *Chemistry & Biology* **6,** 493–505; 10.1016/S1074-5521(99)80082-9 (1999).

14. Blin, K. *et al.* antiSMASH 7.0: new and improved predictions for detection, regulation, chemical structures and visualisation. *Nucleic Acids Research* **51,** W46-W50; 10.1093/nar/gkad344 (2023).

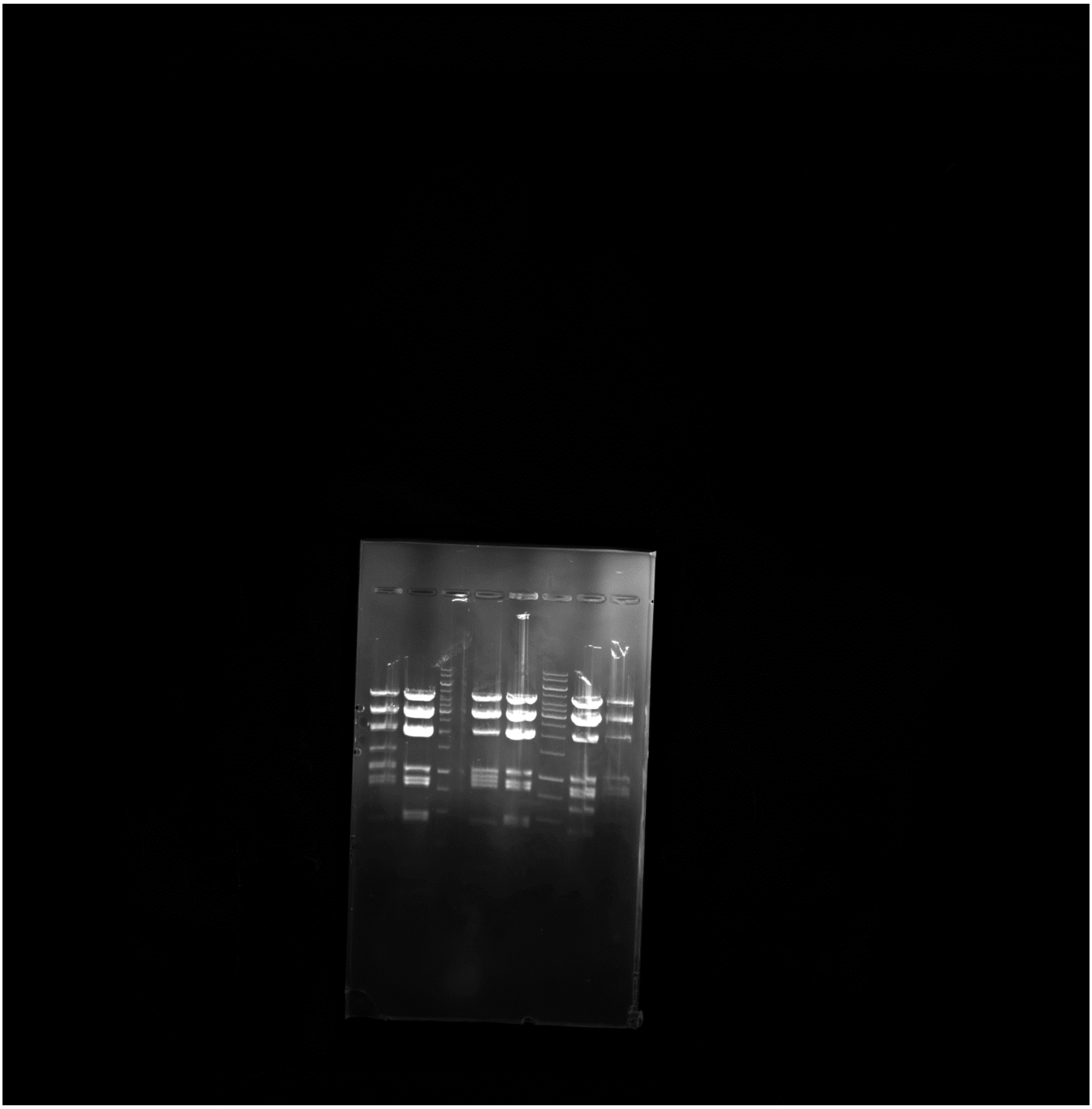

Source data of **Supplementary Fig. 3-b.** Plasmid pQS9-△*pepD* verification.

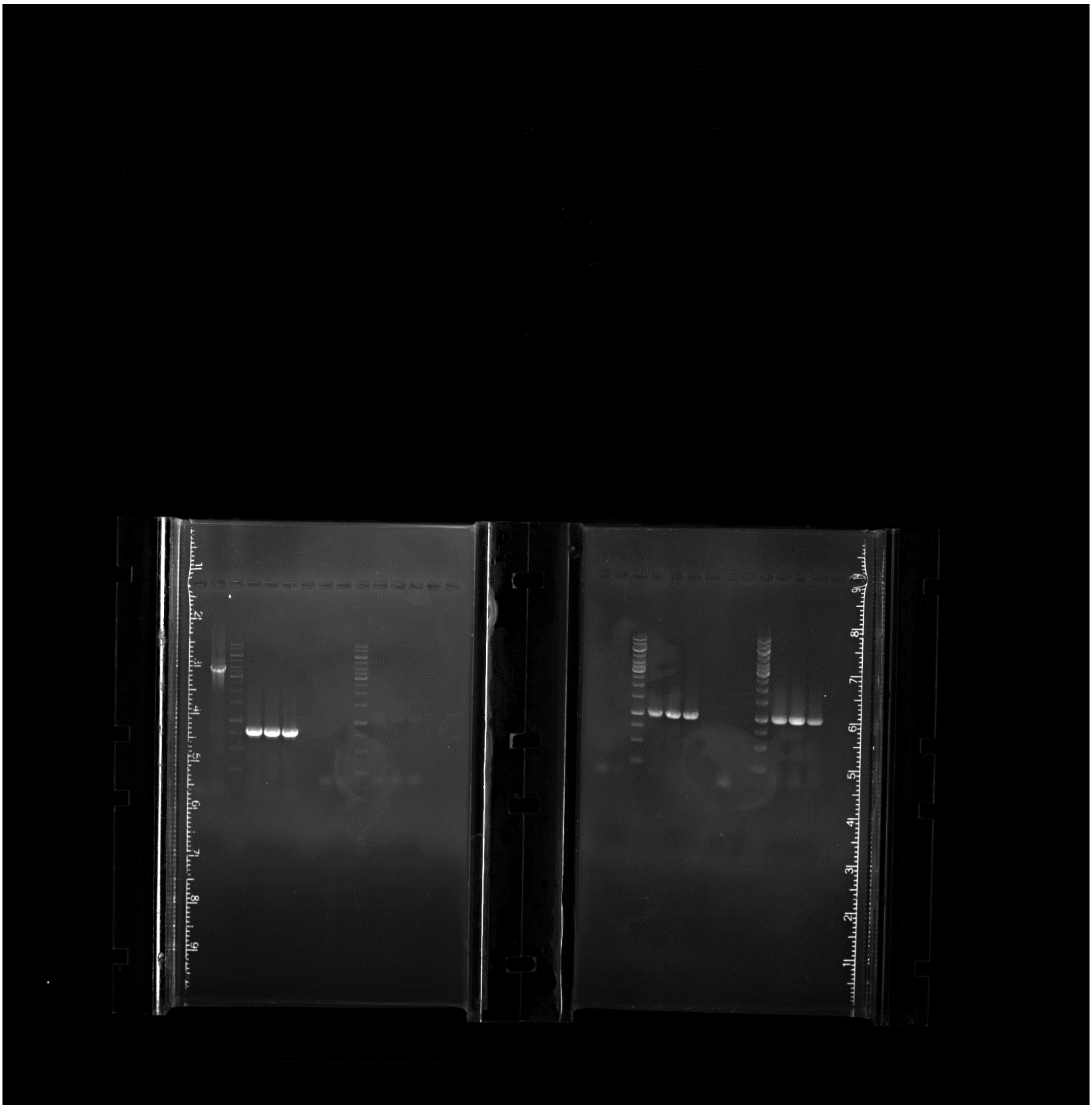

Source data of **Supplementary Fig. 3d.** Mutant *S. catenulae* -△*pepD* verification and **Supplementary Fig. 6b.** PCR verification of *pepJ* gene activation mutant.

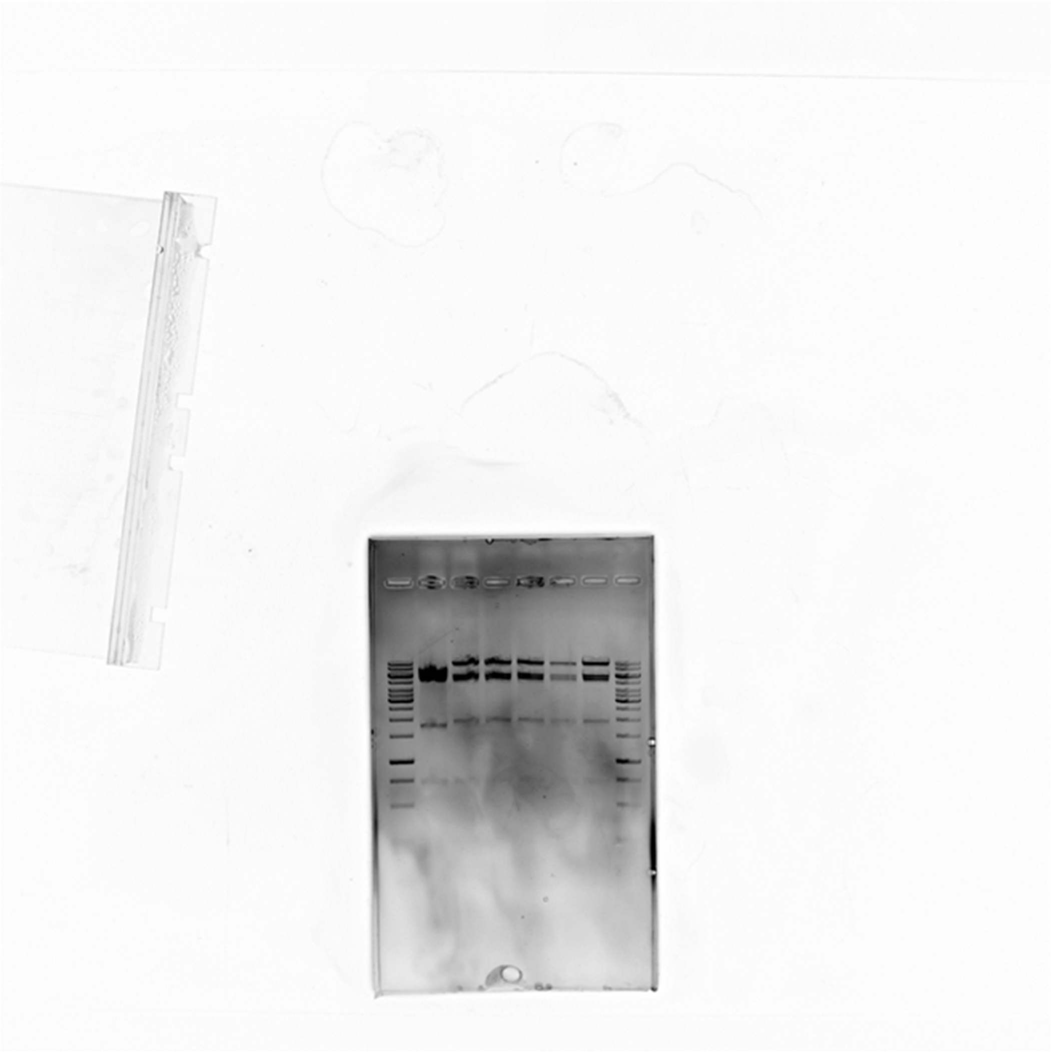

Source data of **Supplementary Fig. 4b.** Plasmid p15A-int-pep verification

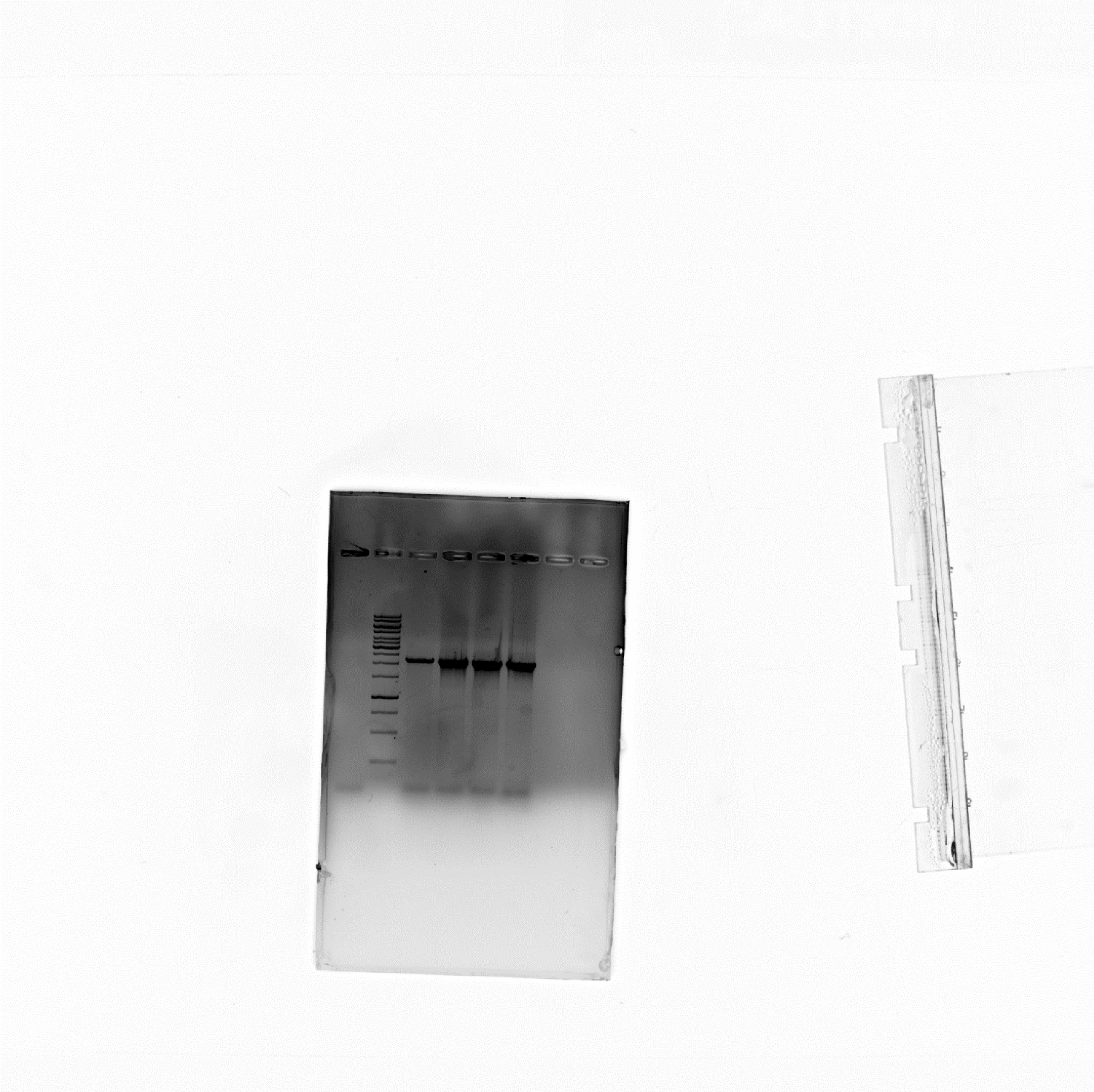

Source data of **Supplementary Fig. 4d.** Mutant *S. albus* Del14-pep PCR verification

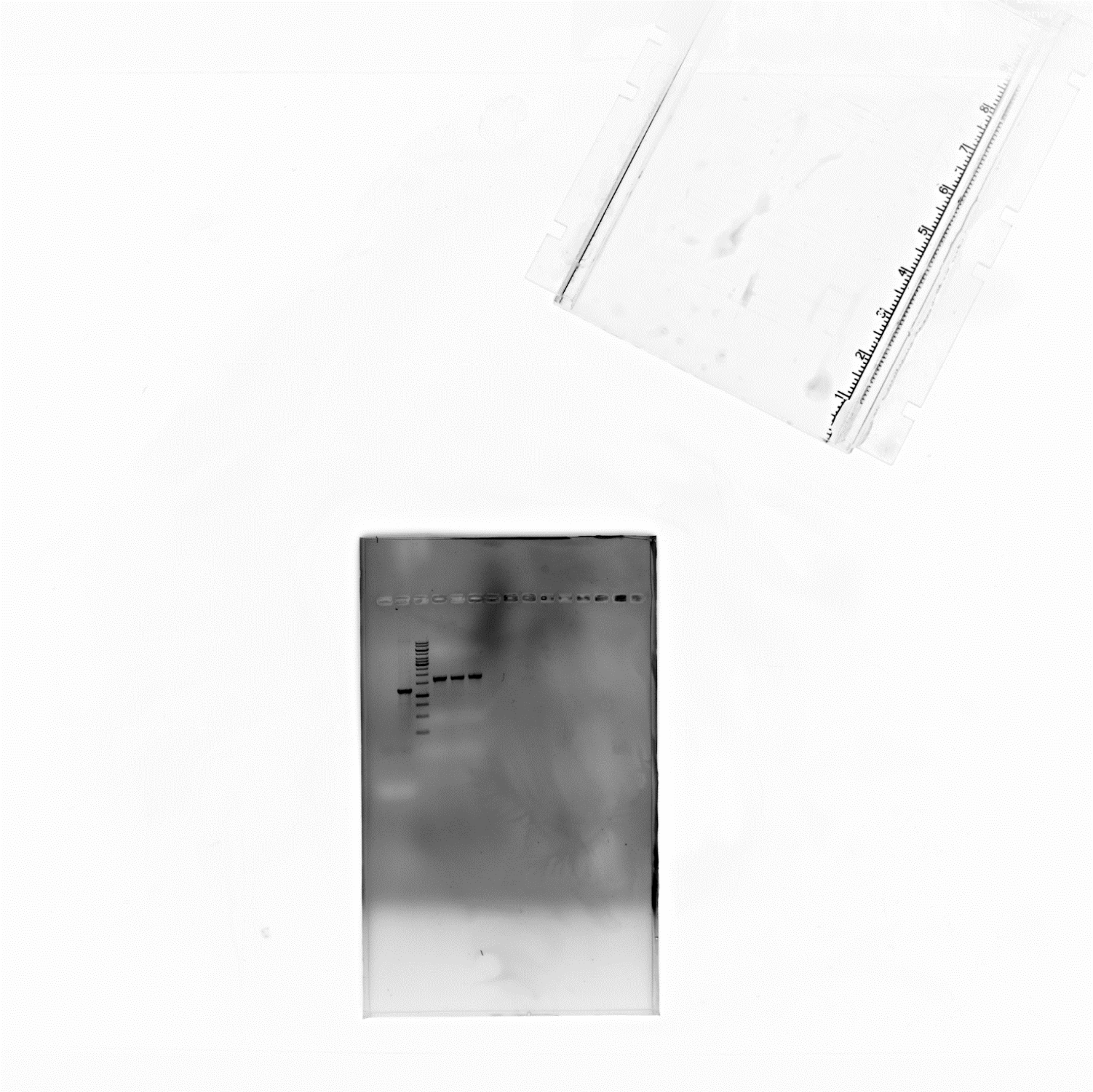

Source data of **Supplementary Fig. 5b.** PCR verification of *pepJ* gene deletion mutant.

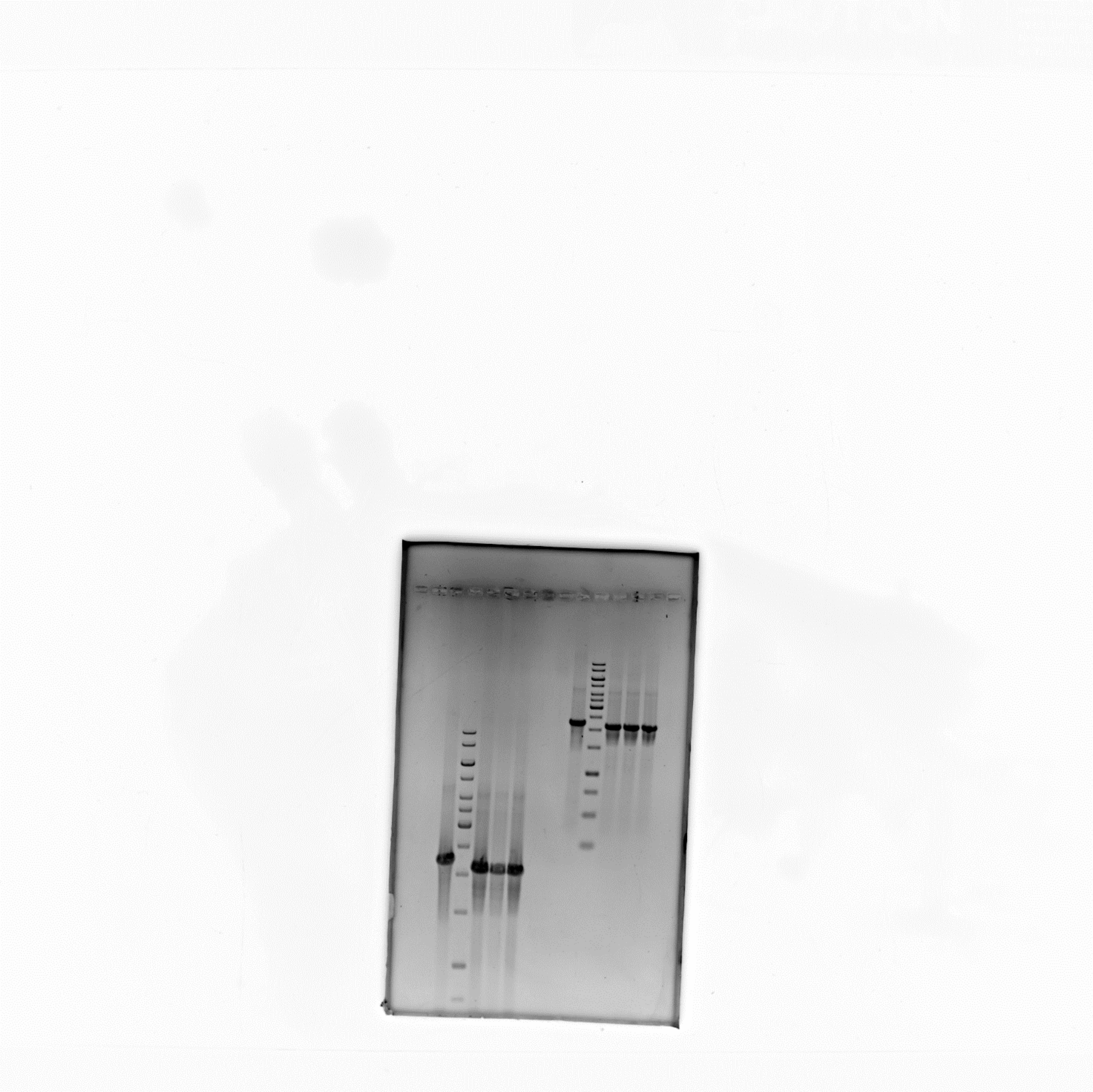

Source data of **Supplementary Fig. 8b.** PCR verification of *S. albus* Del14-*pep*-△*pepI*.

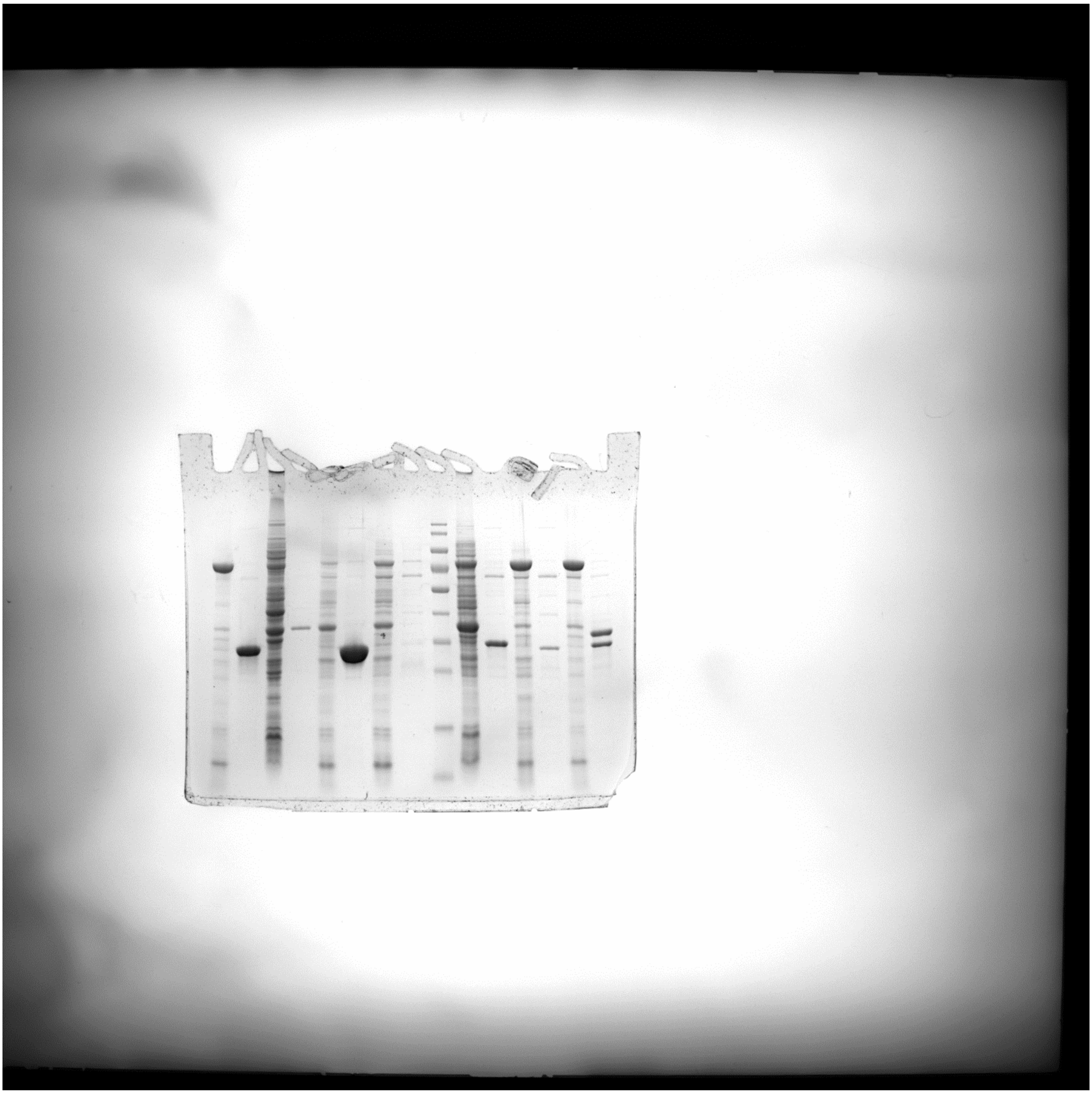

Source data of **Supplementary Fig. 11b.** SDS-PAGE analysis of PepI and PepI homologues.

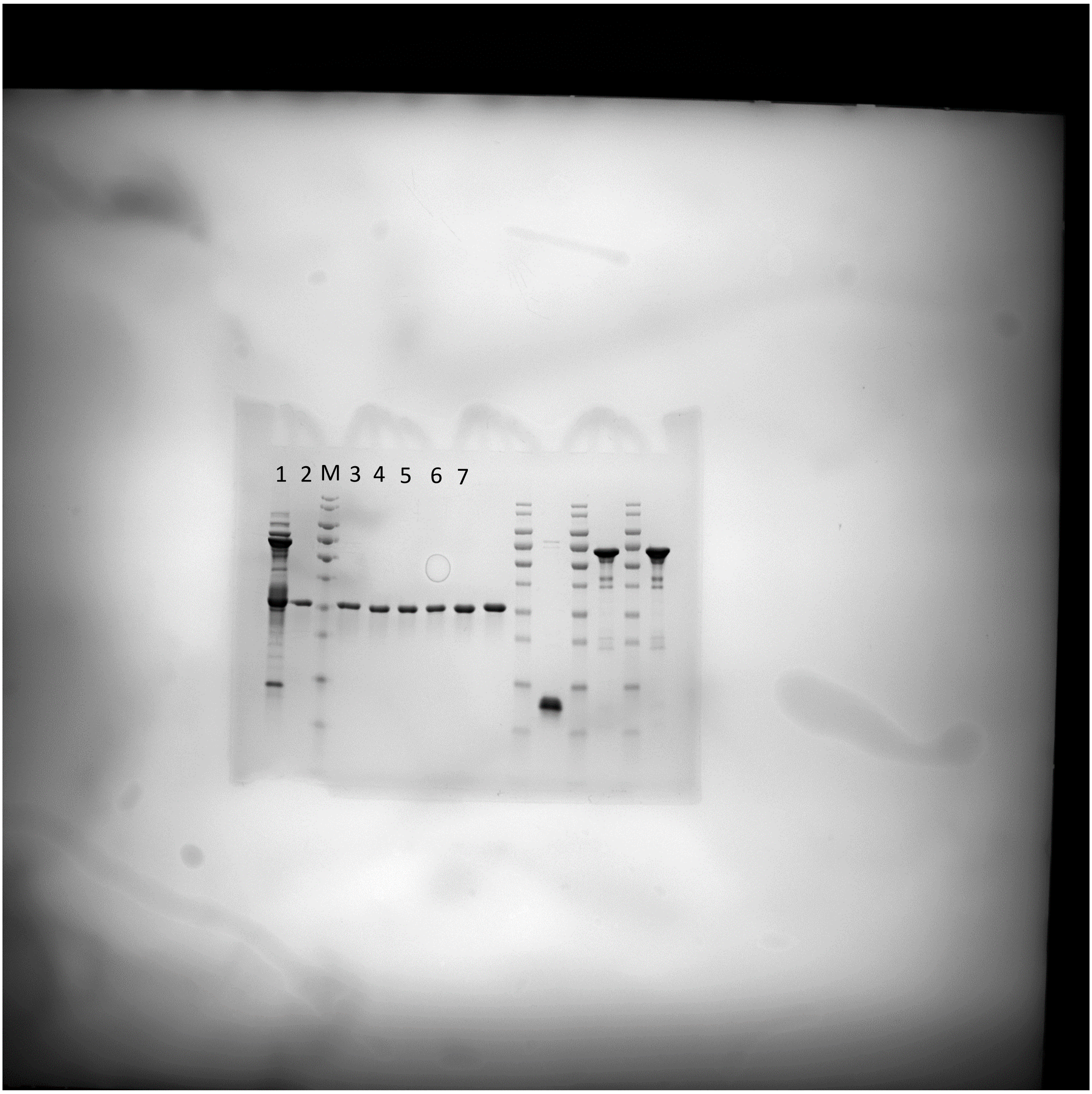

Source data of **Supplementary Fig. 17.** SDS-PAGE analysis of PepI, kvPepI and kvPepI mutants.
